# Development and Validation of a Harmonized TaqMan-Based Triplex Real-Time RT-PCR Protocol for the Quantitative Detection of Normalized Gene Expression Profiles of Seven Porcine Cytokines

**DOI:** 10.1371/journal.pone.0108910

**Published:** 2014-09-30

**Authors:** Anja Petrov, Martin Beer, Sandra Blome

**Affiliations:** Institute of Diagnostic Virology, Friedrich-Loeffler-Institut, Insel Riems, Greifswald, Germany; Institut National de la Santé et de la Recherche Médicale U 872, France

## Abstract

Dysregulation of cytokine responses plays a major role in the pathogenesis of severe and life-threatening infectious diseases like septicemia or viral hemorrhagic fevers. In pigs, diseases like African and classical swine fever are known to show exaggerated cytokine releases. To study these responses and their impact on disease severity and outcome in detail, reliable, highly specific and sensitive methods are needed. For cytokine research on the molecular level, real-time RT-PCRs have been proven to be suitable. Yet, the currently available and most commonly used SYBR Green I assays or heterogeneous gel-based RT-PCRs for swine show a significant lack of specificity and sensitivity. The latter is however absolutely essential for an accurate quantification of rare cytokine transcripts as well as for detection of small changes in gene expressions. For this reason, a harmonized TaqMan-based triplex real-time RT-PCR protocol for the quantitative detection of normalized gene expression profiles of seven porcine cytokines was designed and validated within the presented study. Cytokines were chosen to represent different immunological pathways and targets known to be involved in the pathogenesis of the above mentioned porcine diseases, namely interleukin (IL)-1β, IL-2, IL-4, IL-6, IL-8, tumor necrosis factor (TNF)-α and interferon (IFN)-α. Beta-Actin and glyceraldehyde 3-phosphate dehydrogenase (GAPDH) served as reference genes for normalization. For absolute quantification a synthetic standard plasmid was constructed comprising all target cytokines and reference genes within a single molecule allowing the generation of positive control RNA. The standard as well as positive RNAs from samples, and additionally more than 400 clinical samples, which were collected from animal trials, were included in the validation process to assess analytical sensitivity and applicability under routine conditions. The resulting assay allows the reliable assessment of gene expression profiles and provides a broad applicability to any kind of immunological research in swine.

## Introduction

Cytokines are important mediators that orchestrate cellular functions including inflammatory responses and innate immune reactions. However, excessive activation of the innate immune system in response to pathogens can lead to pathological inflammatory consequences [1], and dysregulation of cytokine responses plays a major role in the pathogenesis of severe and life-threatening infectious diseases including viral haemorrhagic fevers [2]. Another example is the “cytokine storm” that is held responsible for the exceptionally high morbidity and mortality in human highly pathogenic influenza virus infections [3]. Hence, cytokine profiles, especially when targeting a set of cytokines expressed within a certain microenvironment [4], [5], can provide important insights into the development of infectious diseases, which are characterised by an immune pathogenesis such as classical swine fever (CSF), a severe porcine infection that can be accompanied by haemorrhagic lesions. For CSF, a cytokine dysregulation is suspected to be decisive for clinical severity [6]. Similar responses are known for African swine fever (ASF) [7], a disease that recently gained importance through its introduction into several Eastern European countries [8], [9].

Cytokines can be targeted at various levels, from assessment of cellular expression profiles using mRNA detection by RT-PCR, to measurement of intracellular proteins by fluorescence-activated cell sorter staining and quantification of secreted cytokine proteins by the use of bioassays, enzyme-linked immunosorbent assays, radioactive immunosorbent assays, and microarrays [10]. For pathogenesis studies, a combination of expression and protein detection methodologies is usually advisable. However, assessment of expression profiles in pigs is so far severely hampered by the lack of fully validated and reliable diagnostic tools. While several PCR systems for porcine cytokine detection were developed during the last years, most of them either comprised non-standardised heterologous conventional RT-PCR systems [11]–[13] or were performed using intercalating fluorescent dyes such as SYBR-Green I [14]–[18]. However, these techniques have clear disadvantages compared to TaqMan based PCRs particularly with regard to the lack of sensitivity and specificity which are however essential for the accurate quantification of rare cytokine transcripts and the detection of small changes in gene expression.

With the pig as target species, the presented study reports on the design and validation of a harmonized approach for specific detection of cytokine gene expression profiles in swine. Cytokines were chosen to represent different reaction pattern of the immune system (Th1 and Th2 responses), and mediators that are known to be involved in the pathogenesis of important porcine infections such as CSF. To this means, a harmonized multiplexed one-step TaqMan 5' nuclease [19] protocol for specific detection and quantification of seven cytokines, namely interleukin (IL)-1β, IL-2, IL-4, IL-6, IL-8, tumor necrosis factor (TNF)-α and interferon (IFN)-α, was designed and validated. Two reference genes were included to allow reliable normalization.

## Materials and Methods

### 1. Selection of primers and probes

Primers and probes for seven porcine cytokines (IL-1β, IL-2, IL-4, IL-6, IL-8, IFN-α, TNF-α) and two reference genes (β-Actin, GAPDH) were either selected from previous studies [20]–[25] or designed using “Primer-BLAST” (NCBI GenBank). Sequences and corresponding references are shown in table 1. For corresponding alignments Mega 5- and BioEdit software (IBIS Biosciences Carlsbad, USA) were utilized. For all cytokines, probes were labeled with 6-Carboxyfluorecein (FAM), the β-Actin probe with hexachloro-6-carboxyfluorescein (HEX) and the GAPDH probe with Texas Red (TR). The synthesis of oligonucleotides was carried out by biomers.net (Ulm, Germany).

**Table 1:** Sequences of primers and probes.

Gen	Forward- (F) and Reverse- (R) primer	Probe (P)	Length	Reference
*IL-1β*	F-GTGCTGGCTGGCCCACA	CTCTCCACCTCCTCAAAGGG	71 bp	F/P: this study; R: Lange, 2010
	R- GAACACCACTTCTCTCTTCA			
*IL-4*	F-GTCTGCTTACTGGCATGTACCA	CCACGGACACAAGTGCGACATCACCTTAC	117 bp	F/R: Duvigneau et al., 2005
	R-GCTCCATGCACGAGTTCTTTCT			P: this study
*IL-2*	F-TGCTGATCTCTCCAGGATGC	AAGCAGGCTACAGAATTGAAACACCTT	103 bp	F: this study; R: Yang et al., 2012
	R-CCTCCAGAGCTTTGAGTTCTTCTACTA			[47]; P: Duvigneau etal. 2005 [21]
*IL-6*	F-CTGGCAGAAAACAACCTGAACC	TGGCAGAAAAAGACGGATGC	93 bp	F/R: Duvigneau et al., 2005 [21];
	R-TGATTCTCATCAAGCAGGTCTCC			P: this study
*IL-8*	F-AAGCTTGTCAATGGAAAAGAG	TCTGCCTGGACCCCAAGGAAAAGT	101 bp	F/R/P: Lange, 2010 [20]
	R-CTGTTGTTGTTGCTTCTCAG			
*IFN-α*	F-TGGTGCATGAGATGCTCCA	CAGACCTTCCAGCTCT	54 bp	F/R/P: Bautista et al., 2004 [48]
	R-GCCGAGCCCTCTGTGCT			
*TNF-α*	F-AACCTCAGATAAGCCCGTCG	CCAATGCCCTCCTGGCCAACG	128 bp	F/R/P: Lange, 2010 [20]
	R-ACCACCAGCTGGTTGTCTTT			
*β-Actin*	F-AGCGCAAGTACTCCGTGTG	TCGCTGTCCACCTTCCAGCAGATGT	105 bp	F modified/R/P:
	R-CGGACTCATCGTACTCCTGCTT			Toussaint et al., 2007 [49]
*GAPDH*	F-ACATGGCCTCCAAGGAGTAAGA	CCACCAACCCCAGCAAGAGCACGC	105 bp	F/R/P: Demissie et al., 2004 [21]
	R-GATCGAGTTGGGGCTGTGACT			

FAM 5′ modification was used for IL-1β, IL-2, IL-4, IL-6, IL-8, IFN-α, TNF-α; HEX for β-Actin; Texas Red for GAPDH. BHQ (Black-Hole-Quencher)-1 was used for 3′ modifications of IL-1β, IL-2, IL-4, IL-6, IFN-α, β-Actin and GAPDH; BHQ-2 was used for of TNF-α and IL-8. Corresponding references are given in the right column. Sequences marked with “this study” were created by the use of “Primer-BLAST” available on NCBI GenBank.

F =  Forward Primer; R =  Reverse Primer; P =  Probe; bp = base pairs.

### 2. In vitro generation of positive control RNA

#### 2.1. Generation of peripheral blood mononuclear cells (PBMCs)

Approximately 50 ml of porcine EDTA blood were overlayed with the equal amount of lymphocyte separation medium LSM 1077 (PAA Laboratories GmBH, Pasching, Austria) and a density gradient centrifugation at 580 *g* for 40 min at 20°C without brake was performed. The leucocyte phase was collected and washed with 0.8 mM EDTA solubilized in phosphate buffered saline (PBS^-^) for removal of separation medium. Thereafter, the remaining erythrocytes were removed through lysis with buffered ammonium chloride solution (containing 153 mM NH_4_Cl, 10 mM KHCO3, 1 mM EDTA to 1 l (pH 7.4)). To this means, the threefold volume of lysis buffer was added and incubated at 4°C for 15 min. The resulting PBMC suspension was washed with PBS^-^ and cultured in DMEM medium containing 10% fetal bovine serum, 20 mM HEPES including penicillin-streptomycin (“Anti-Anti (100X)”), Antibiotic-Antimycotic from GIBCO by Life Technologies, Carlsbad, California, USA) at approximately 10^7^ cells/ml. After 16 h of incubation at 37°C in a 5% CO_2_ atmosphere, non-adherent cells where removed through washing with sterile pre-warmed PBS^-^ , and the cleaned PBMC cell suspension was incubated for one to three days for maturation under the same culture conditions until exposure to different cytokine-stimulators.

#### 2.2. In-vitro stimulation of cytokines

Different mitogens and antigens were used for stimulation of the desired cytokines as previously described [26]–[29]. Details are depicted in table 2. The stimulating agents were obtained from Sigma (Sigma-Aldrich, St. Louis, Missouri, USA). After a maturation time for PBMCs of one to three days, stimulators were utilized at the following concentrations: Lipopolysaccharide (LPS) 20 µg/ml, all other stimulating agents (Peptidoglycan, PGN; Concanavalin A, ConA; phytohaemagglutinin; PHA; pokeweed mitogen, PWM) 5 µg/ml. Along with the stimulating agents, cells were incubated for approximately 18 h at 37°C with 5% CO_2_ in 6-well plates until the expected cytokine expression optimum was reached. For RNA extraction, cells were harvested and subjected to RNA extraction using the methods described below.

**Table 2:** In-vitro cytokine stimulation.

Cytokine	Stimulating agents
*IL-1β*	LPS
*IL-4*	PWM + ConA + PHA
*IL-2*	PWM + ConA + PHA
*IL-6*	PWM + ConA + PHA
*IL-8*	LPS
*IFN-α*	PGN + LPS + ConA
*TNF-α*	PGN + LPS + ConA

The stimulating agents are presented along with the corresponding target cytokines as well as background information about their functionality.

LPS = *Salmonella typhimurium* lipopolysaccharid; a component of the outer gram positive bacteria membrane, antigenic effect on PBMCs.

PGN =  *Staphylococcus aureus* peptidoglycan; a stabilizing macro molecule in the cell wall of gram positive bacteria; antigenic effect on PBMCs.

ConA =  Concanavalin A; a lectin from the jack bean, mitogenic effect (especially on T-cells).

PWM = Pokeweed mitogen; a lectin of the American pokeweed, activating effect on B- and T-cells.

PHA =  Phytohemagglutinin; a herbal lectin, mitogenic effect (especially on T-cells).

### 3. RNA isolation

RNA extraction of different sample matrices was performed using Trizol Reagent (Life Technologies) in combination with the automated MagAttract Virus Mini M48 Kit (QIAGEN GmbH, Hilden, Germany) on the King Fisher 96 Flex instrument (Thermo Scientific) as previously described [30].

### 4. Analyses of expression stability of reference genes

Confirmatory analyses of stable expressions of β-Actin and GAPDH comprised the following tests. Firstly, *in vivo* generated PBMCs (see section 2.2.1) were exposed to different stimulating agents (see section 2.2.2 and table 2) while several wells were left untreated by incubating them only with cell culture media each time. PBMC RNA was extracted in different time intervals after stimulation (after 1, 12, 18, 24, 36, 48 and 60 hours) and RT-qPCRs targeting β-Actin and GAPDH were performed comparing the quantification cycle (Cq)-values of stimulated and untreated PBMC RNA. Secondly, RNA from EDTA blood derived from pigs infected with the highly virulent CSFV-strain “Koslov” were used in RT-qPCR. Cq-values of β-Actin and GAPDH were detected prior to infection and compared to measurements at different time intervals after infection.

### 5. Construction of synthetic standard RNA

**Figure 1:**
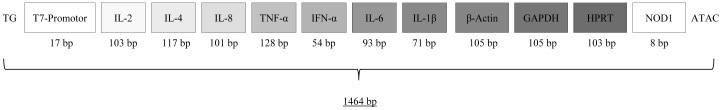
Composition of the synthetic standard gene comprising all target cytokines (IL-2, IL-4, IL-8, TNF-α, IFN-α, IL-6, IL-1β) and internal reference genes (β-Actin, GAPDH, HPRT (Hypoxanthin-Guanin-Phosphoribosyltransferase), starting with the T7-promotor sequence and concluding with the NOD1 restriction site as initial point for linearization and transformation to RNA. Each target cytokine was included with a nucleotide overhang of approximately 50 base pairs (bp) prior to forward primer sequence. In total, the synthetic standard gene comprises 1464 bp.

A synthetic gene comprising all target cytokines and reference genes (see figure 1) was constructed and synthesized by GeneArt Gene Synthesis (Life Technologies). The Kanamycin-resistant gene was transformed in corresponding resistant bacteria after permeabilization. The bacteria plasmid was purified with QIAfilter Plasmid Maxi Kit (Qiagen, Venlo, Netherlands) and the nucleic acid concentration was determined with a NanoDrop 2000c Spectrophotometer (PEQLAB Biotechnologie GmbH, Polling, Austria). To verify the transformation process the plasmid was sequenced using the Big Dye Terminator v1.1 Cycle sequencing Kit (Applied Biosystems). Nucleotide sequences were read with an automatic sequencer (3130 Genetic Analyzer, Applied Biosystems) and analyzed using the Genetics Computer Group software version 11.1 (Accelrys Inc., San Diego, USA). Thereafter, the DNA-plasmid was used for synthesis of heterologous RNA. It was cleaved at the attached NOD1-restriction site with NOD1 enzyme (New England Biolabs, Ipswitch, Massachusetts, USA) and linearized DNA strands were filtrated and eluted by using QIAquick Nucleotide Removal Kit (Qiagen). The obtained DNA was in vitro transcribed using T7 RNA Polymerase (Promega Corporation, Madison, USA). Subsequently, the DNA matrix was removed through DNase I digestion (RQ1 RNase-Free DNase, Promega). The gained RNA was visualized by 1% agarose gel electrophoresis. RNeasy Mini Kit (Qiagen) was utilized in combination with Trizol Reagent (Life Technologies) and DNA digestion (with RNase-Free DNase Set (Qiagen)) for a final RNA cleanup. Finally, RNA concentration was determined using the NanoDrop spectrophotometer (Peqlab) and the concentration was set to 2×10^9^ copies/µl. A 10-fold standard dilution series was generated in RNA-safe buffer (50 ng/µl of carrier polyA-RNA, 0.05% Tween-20, 0.05% sodium azided in RNase-free water). Dilutions from 2×10^1^ to 2×10^6^ copies/µl were employed for subsequent RT-qPCRs.

### 6. RT-qPCR

Prior to implementation in RT-qPCR systems, 10-fold dilution series of each *in-vitro* generated positive control RNA and the synthetic standard RNA were amplified in conventional RT-PCR, visualized in 3% agarose gel electrophoresis and verified by sequencing. Sequence data were obtained from the NCBI GenBank and corresponding alignments were carried out with Mega 5- and BioEdit software (IBIS Biosciences Carlsbad, USA).

In a first step, single-tube assays were designed for each cytokine or reference gene as basis for the subsequent development of the triplex RT-qPCR protocol using the AgPath-ID One-Step RT-PCR reagents (Ambion-Applied Biosystems, Thermo Fisher Scientific by Life technologies) for simultaneous detection of one target cytokine and two reference genes.

Subsequently, tests for the reduction of the total mastermix reaction volume from 25 µl to 12.5 µl were carried out. Thereby, the mastermix for a single reaction comprised 0.25 µl RNase-free water, 6.25 µl 2× RT- PCR Buffer, 0.5 µl of 25× RT- PCR Enzyme Mix, 1 µl FAM-labelled cytokine-primer-probe mix, 1 µl of each reference gen-primer-probe mix and finally 2.5 µl of template RNA.

All RT-qPCRs were performed with a Bio-Rad CFX 96 Real-Time Detection Systems (Bio-Rad, Hercules, CA, USA). Protocols for all cytokine RT-qPCRs were adjusted to the same thermal profile: reverse transcription at 45°C for 10 min, followed by PCR activation for 10 min at 95°C and 45 cycles including denaturation phase at 95°C for 15 sec, annealing at 57°C for 20 sec and elongation for 30 sec at 72°C. Data were collected during the annealing phase.

Oligonucleotide concentrations were optimized through checkerboard titrations. Furthermore, confirmatory tests for the absence of residual DNA in isolated RNA samples were performed by deployment of the one- and two-step RT-qPCR chemistry from Promega. For that purpose, RNA samples were tested using both systems under the same conditions while the two-step assay was carried out without adding the enzyme for reverse transcription.

Moreover, reproducibility of all assays was tested using the standard RNA in triplicates and deviations of Cq-value were determined.

In addition, 402samples gathered from pigs infected or vaccinated with CSFV (leucocyte samples) and infected with ASFV (EDTA blood samples) during several animal trials carried out at the Institute of Diagnostic Virology, Friedrich-Loeffler-Institut, Insel Riems, Germany (sample repository) were tested in all seven triplex RT-qPCRs (see table 3). In the experimental studies involving live animals, all applicable animal welfare regulations, including EU Directive 2010/63/EC and institutional guidelines, were taken into consideration. The animal experiments were approved by the competent German authority (Landesamt für Landwirtschaft, Lebensmittelsicherheit und Fischerei Mecklenburg-Vorpommern) under reference numbers 7221.3-1.1-015/12 and 7221.3-1.1-018/12.

**Table 3:** Samples from different animal trials (n = 402) used for assay validation.

Sample status	Sample matrix	Domestic pigs	Wild boar
*Control*	EDTA	3	3
*Control*	PBMC	49	24
*CSFV infected*	PBMC	109	45
*CSFV vaccinated*	PBMC	97	55
*ASFV infected*	EDTA	17	/

Samples were chosen to represent different pig species (wild boar, domestic pigs) and inoculation status (CSFV infection/vaccination, ASFV infection, corresponding control animals). Moreover, PBMC and EDTA blood samples were included.

## Results

### 1. Confirmation of identity and stable expression of reference genes

Identity of all cytokines and reference genes could be confirmed by alignments with sequences available through NCBI GenBank. No indications for possible cross-reactions were observed. Absence of residual DNA in extracted RNA samples was proven by tests for no-reverse transcription (RT) as described in section 2.6.

RT-qPCRs conducted for the assessment of β-Actin- and GAPDH as reference targets (see section 2.4) revealed no coherent changes in expression levels after different stimulation or infection and thus, suitability for gene expression normalization was confirmed.

### 2. Sensitivity

For first assessment of analytical sensitivity, a 10-fold dilution series of the positive control RNA derived from the synthetic plasmid was employed for testing each cytokine and reference gene. The resulting standard curves from all single tube assays are shown in figure S1, starting from 2×10^7^ copies/µl as highest standard to 2×10^1^ copies/µl as lowest. Corresponding Cq-values and efficiencies for all assays are listed in table S1. The last employed standard dilution of to 2×10^1^ copies/µl could be detected by each assay except for β-Actin which showed a detection limit of 2×10^2^ copies/µl. Cq-values of standard RNA dilutions ranged between 15 for the highest standard and 35 for the lowest (see table S1).

Sensitivity was further analyzed by testing *in vitro*-generated positive RNA in 10-fold dilutions from 10^−1^ to 10^−7^. The measured Cq-values as well as limits of detections are shown in table S1 ranging from a dilution of 10^−3^ (for IL-6 and IFN-α) to more than 10^−7^ for IL-8.

**Figure 2:**
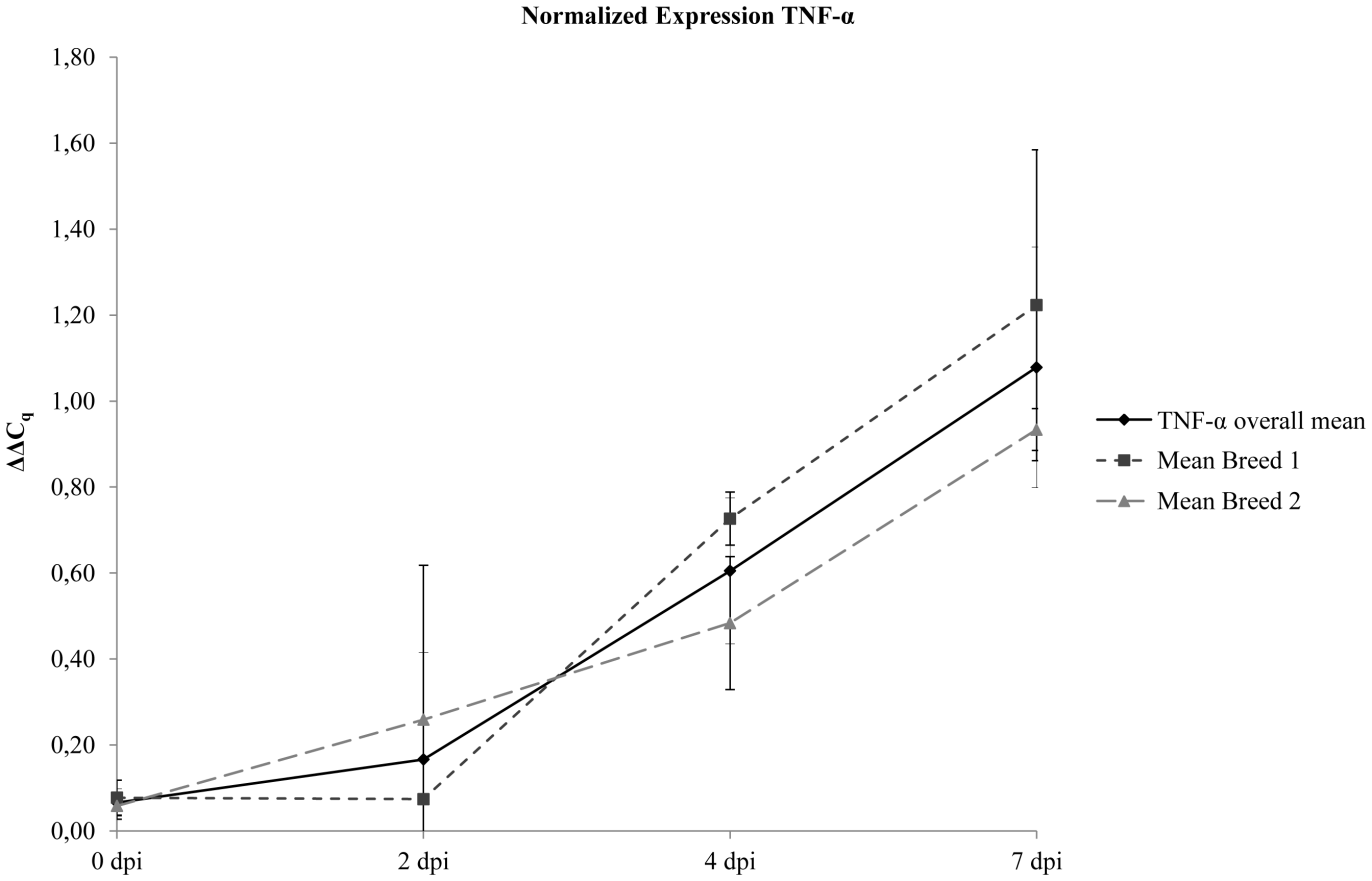
Detection of normalized TNF-α gene expression (ΔΔCq) in leukocyte samples from CSFV infected pigs. Samples were obtained from two different pig breeds at days 0, 2, 4, and 7 post infection (dpi). Results are given as mean values: in total from all animals (TNF-α overall mean) and separately for each breed (Mean Breed 1, Mean Breed 2). Bars indicate standard deviations.

**Figure 3:**
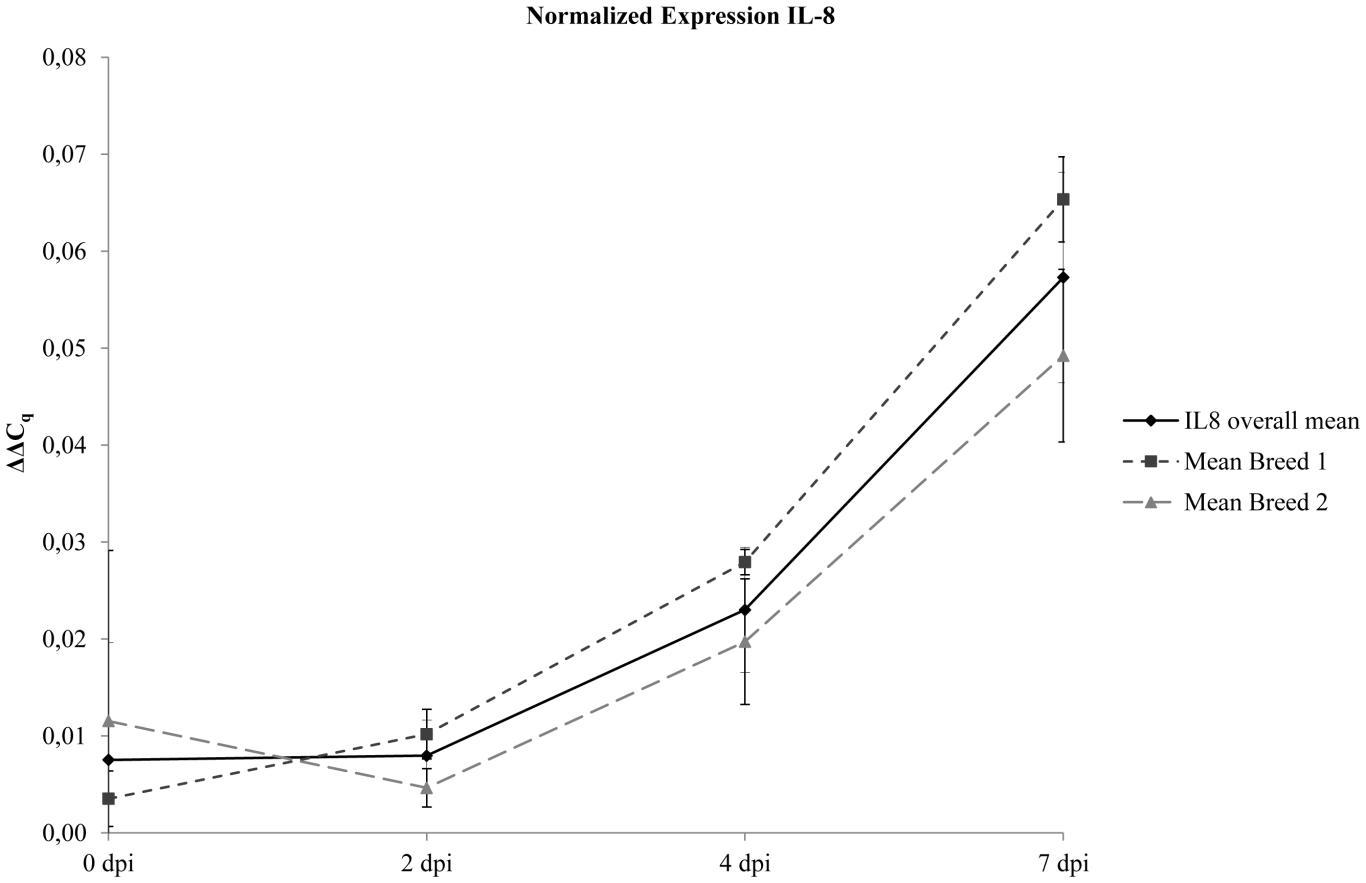
Detection of normalized IL-8 gene expression (ΔΔC_q_) in leukocyte samples from CSFV infected pigs. Samples were obtained from two different pig breeds at days 0, 2, 4, and 7 post infection (dpi). Results are given as mean values: in total from all animals (IL-8 overall mean) and separately for each breed (Mean Breed 1, Mean Breed 2). Bars indicate standard deviations.

Finally, the applicability of all assays for routine pig samples (EDTA blood and white blood cells) could be demonstrated by testing a total number of 402 samples from pigs infected with different CSFV strains, ASFV “*Armenia08*”or from CSFV vaccinated pigs. Exemplary results for normalized TNF-α and IL-8 expression (ΔΔC_q_) after infection of two different pig breeds with the highly virulent CSF virus strain “Koslov” are depicted in figures 2 and 3, respectively. Additionally, detailed information comprising the results of all seven triplex assays including Cq values and corresponding normalized gene expressions are provided in table S5. To link gene expression with protein detection, exemplary results are depicted in figure S2.

### 3. Implementation of seven cytokine triplex RT-qPCR assays

In order to detect one cytokine and two reference genes simultaneously a triplex protocol was developed (as described in section 2.6). Checkerboard titrations of all primers and probes revealed the following optimal and harmonized concentrations: for all cytokines the harmonized protocols uses 10 pmol primers, 2.5 pmol probe, and for reference genes 2.5 pmol primers, 1.25 pmol probe.

A comparison between single and triplex assays was performed for each cytokine by using the standard RNA and *in vitro*-generated positive RNA in 10-fold dilutions. The comparative results are presented in table S2.

**Figure 4:**
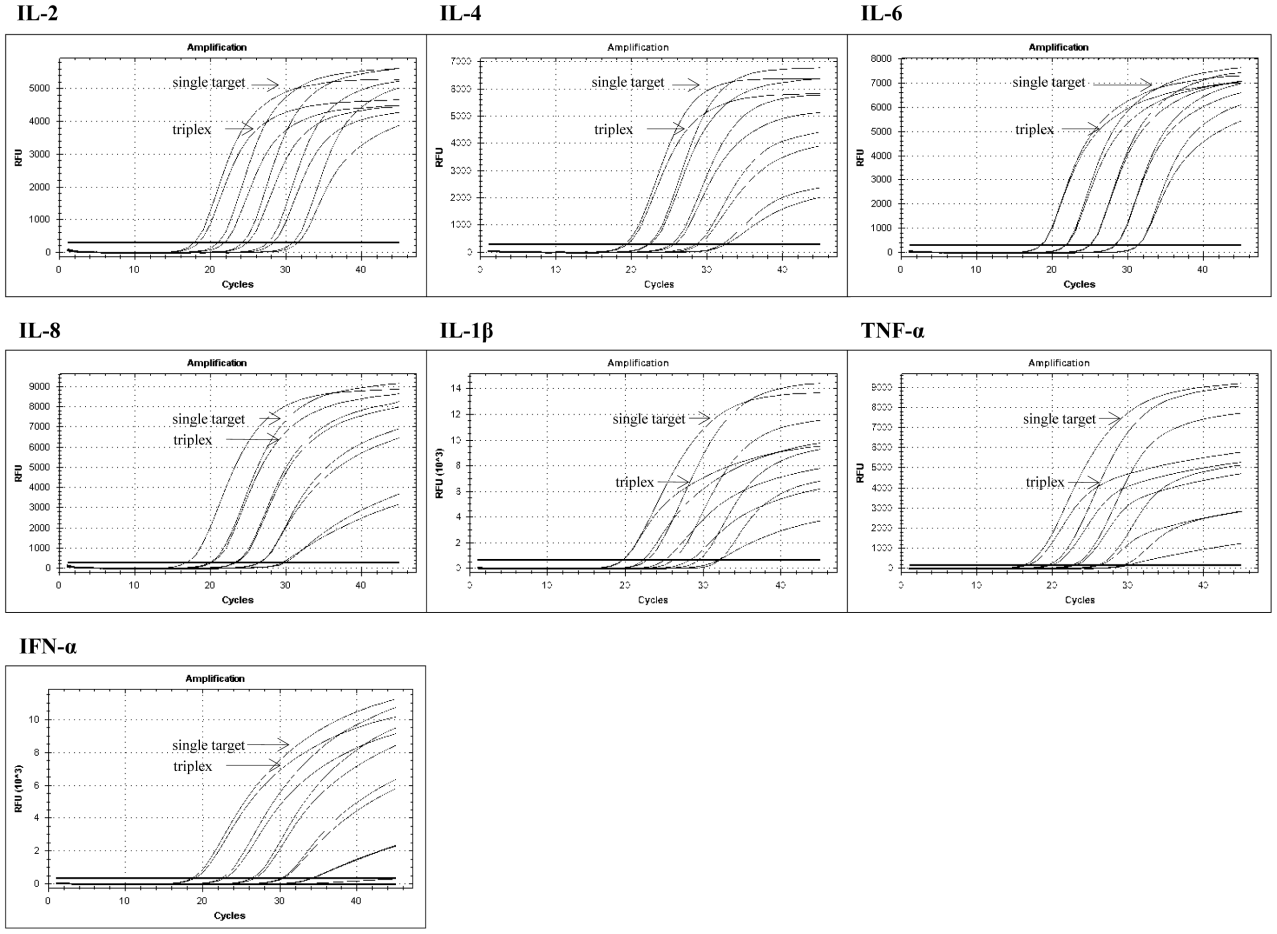
Comparative amplifications of 10-fold diluted standard RNA ranging from 2×10^2^ to 2×10^7^ copies/µl of single target and multiplex assays of all target cytokines (FAM). The vertical axis demonstrate fluorescence levels (relative fluorescence units, RFU), the horizontal axis shows the number of cycles. Comparative illustrations of end fluorescence levels of standard curves are shown illustrating higher end fluorescences in single target compared triplex assays in which target cytokines were detected simultaneously with β-Actin (HEX) and GAPDH (TR).

The analyses revealed variations of Cq-values between single and multiplex assays of less than 2 Cq-values in all cases apart from two exceptions, in which higher deviations were found within the last dilution steps of positive RNA (IFN-α, IL-1β). Each single and triplex assay was able to detect the lowest deployed standard (see table S2). Furthermore, losses of end fluorescence levels (End RFUs) did not obviously influence final results (see figure 4).

All validation experiments of the triplex RT-qPCR protocol were performed with a total mastermix reaction volume of 12.5 µl including 2.5 µl RNA-template. Comparative analyses of full (25 µl) and halved (12.5 µl) approaches revealed no notable differences of Cq-values, detected genome copies and fluorescence levels (RFUs) as shown in table S3. Cq-losses higher than 3 were only observed in some of the lowermost dilution steps within the standard- and positive RNA 10-fold dilution series. For example, the lowest standard of 2×10^1^ copies/µl revealed Cq-losses in assays targeting IL-2, IL-8 and IL-1β (FAM) or by targeting β-Actin (HEX) in the IFN-α triplex assay and GAPDH (TR) in the IL-2, TNF-α and IFN-α triplex assays respectively. Apart from that, no notable differences of Cq-values or absolute quantities were measured. End fluorescence levels revealed differences between approximately 500 to maximum deviations of 3000 (see table S3).

Finally, reproducibility was tested as described in section 2.6 and could be confirmed by showing no notable differences in Cq-values between the standard RNA triplicates in all assays (see table S4). Cytokine Cq-deviations were below 1 in the majority of cases which corresponds to a less than with a less than 3-fold deviation. In general, variation was mainly observed in higher dilutions (lowest target concentrations, see table S4).

## Discussion

Cytokines are powerful mediators of the immune system and have a key role in the selection of immunological pathways and link innate and adaptive immune responses. To date, many basic immune pathological mechanisms e.g. for haemorrhagic diseases like CSF and ASF have not been clearly defined showing the need for reliable detection tools in order to characterize beneficial or detrimental reaction patterns. The selection of target cytokines for this study pursued the objective of covering a preferably wide range of immunological events in swine. While IL-2 can be regarded as indicator for the Th1 pathway, IL-4 is indicative for the Th2 response respectively [31]. The endogenous pyrogen TNF-α is of great importance as it can provoke shock symptoms upon systemically release. Yet, it also has beneficial abilities through a local restrictive effect after infection [31]. Especially in the context of CSF pathogenesis, it has been proven one of the most crucial cytokines [32]. In this context, inclusion of the TNF-α induced proinflammatory cytokines IL-1β and IL-6 seems reasonable for their potential of acting either pyrogenic or activating monocytes and natural killer cells [31]. In contrast, IL-8 which can be produced e.g. by T-helper cells can be indicative for the Th2 pathway possessing the abilities of attracting neutrophil granulocytes, lymphocytes and of contributing to angiogenesis [31]. An IL-8 dysregulation is suspected to be involved in CSF development [20]. Finally, as an effective mediator of antiviral resistance, IFN-α is particularly involved in important mechanisms of innate immunity [33] and was therefore included in the established assay. Taken together, the selected cytokines represent valuable immunological markers by giving information about complex immunological responses.

Among the most suitable techniques for molecular cytokine research is the highly sensitive one-step RT-qPCR system [34] which allows quantitative analyses as well as multiplexing. As already stated by Huggett et al., the enhanced specificity of TaqMan-based real-time assays is greatly advantageous for immunological research since many cytokines appear in such low abundances that detection of their mRNA by real-time RT-PCR represents the only method which is sensitive enough for reliably measuring their expression *in vivo* [24], [35]. So far, gel-based PCR systems have been applied widely for cytokine detection [11], [13] despite their disadvantage of being not truly quantitative and often leading to an underestimation of total mRNA levels because of common depletion of reagents during the reaction [36]. Consequently, PCR products are not proportional to the amount of initial target when visualised on a gel [37]. The most widely used SYBR-Green I assays [38]–[42] have the disadvantages of potentially generating primer-dimers, the indiscriminately binding to all double-stranded DNA which might lead to a formation of secondary structures, and to a possibly limiting primer-concentration as well as to an overestimation of target-DNA [43]. To overcome these problems and to add specificity, a fluorogenic probe based approach was chosen in the presented study. The probe detection system in TaqMan PCRs make those assays clearly advantageous in comparison to SYBR Green and conventional PCR methods as they provide a high level of target specificity [19]. Specificity is particularly difficult to prove in immunological assays as truly negative biological samples are difficult to obtain (e.g. stress reactions or previous pathogen contact). However, “negative” control samples were involved in the establish procedure either originating from *in vitro* generated PMBCs or from pigs of untreated control groups. To prove the “diagnostic” performance of the established assay more than 400 samples were collected during several animal trials including CSFV vaccination and infection as well as ASFV infection and were tested in all seven triplex assays. However, due to the above mentioned reasons, comparative evaluations of true “positive” and “negative” pigs concerning specific cytokine gene expressions was problematic. During the development procedure, the “Assay validation pathway” [44] was implemented as far as for this purpose possible by detecting analytical performance characteristics. The assessment of repeatability revealed a high level of agreement between triplicates of synthetic standard RNA by showing only minor deviations while increased variations were exclusively found in dilution steps with lowest concentrations as shown in table S4. Furthermore, limits of detections were assessed for determining the analytical sensitivity for each, single-target test (see table S1), comparative analyses between single target and multiplex assays (see table S2) as well as the comparison between a full mastermix reaction volume of 25 µl and a halved volume of 12.5 µl (see table S3). Indeed detection limits partly showed decreases in triplex assays compared to single target PCRs and also in the halve approach compared to the full, but these were measured in negligible amounts or exclusively within the least dilutions of the standard RNA or biological control RNA. Thereby, it could be proven that RT-qPCR chemistry as well as the sample volume could be successfully halved making the assay much more cost-effective and that simultaneous detection of one target cytokine and two reference genes is possible which allows an accurate determination of gene expression profiles by normalization. Another advantage of inclusion of two reference genes is to control varying amounts of input RNA used in the reverse transcription step [34]. This is particularly useful regarding the high variability of biological sample material. Different stimulation and infection experiments were successfully conducted for further confirmation of stable β-Actin and GAPDH expressions [45] despite this was already shown by preliminary studies [46].

First implementation of the assays in different animal trials (see examples in table S5) showed that the choice of sample matrices and sample handling (especially leukocyte preparation and freeze/thawing) had a strong impact on the detection of cytokine gene expression. While *in vitro* stimulations proved that the respective cytokine mRNAs were reliably detected (see results for positive RNAs), diagnostic samples often resulted in negative results (if normalized gene expression was assessed). In this context, further validation and optimization for sample transport (direct cooling), preparation (avoiding freeze/thawing) and extraction (direct sample suspension in Trizol or equivalents) is clearly needed.

## Conclusions

A one-step TaqMan-based triplex RT-qPCR protocol was established and validated for the accurate and reliable detection and quantification of seven porcine cytokines (IL-1β, IL-2, IL-4, IL-6, IL-8, TNF-α, IFN-α) representing immunological markers by covering a broad range of host responses. These real-time assays were successfully harmonized by using a unique RT-qPCR protocol along with the same chemistry, temperature profile and synthetic standard resulting in a simple, cost-efficient, specific and highly sensitive assessment of normalized gene expression profiles. This novel and versatile tool will aid not only studies of swine fever pathogenesis but also swine immunology in general.

## Supporting Information

Figure S1 **Amplifications of all single assays of 10-fold diluted standard RNA ranging from 2×10^1^ to 2×10^7^ copies/µl.**
(TIF)

Figure S2 **Exemplary comparison of gene expression and cytokine protein detection using ELISA systems (IL-6 and IL-8).**
(TIF)

Table S1 **RT-qPCR results of all single target assays.**
(DOC)

Table S2 **Comparison of single target and triplex RT-qPCR results.**
(DOC)

Table S3 **Comparison of RT-qPCRs between a total mastermix reaction volume of 25 µl and the halved volume of 12.5 µl.**
(DOC)

Table S4 **Assessment of reproducibility by the use of a synthetic standard RNA dilution series as triplicates.**
(DOC)

Table S5 **Testing of experimental samples.**
(PDF)
