# Supplementary figures and images for: Development and Validation of a Harmonized TaqMan-Based Triplex Real-Time RT-PCR Protocol for the Quantitative Detection of Normalized Gene Expression Profiles of Seven Porcine Cytokines
Source: PLoS One. 2014 Sep 30;9(9):e108910. doi: 10.1371/journal.pone.0108910 (PMC4182501; doi:10.1371/journal.pone.0108910)

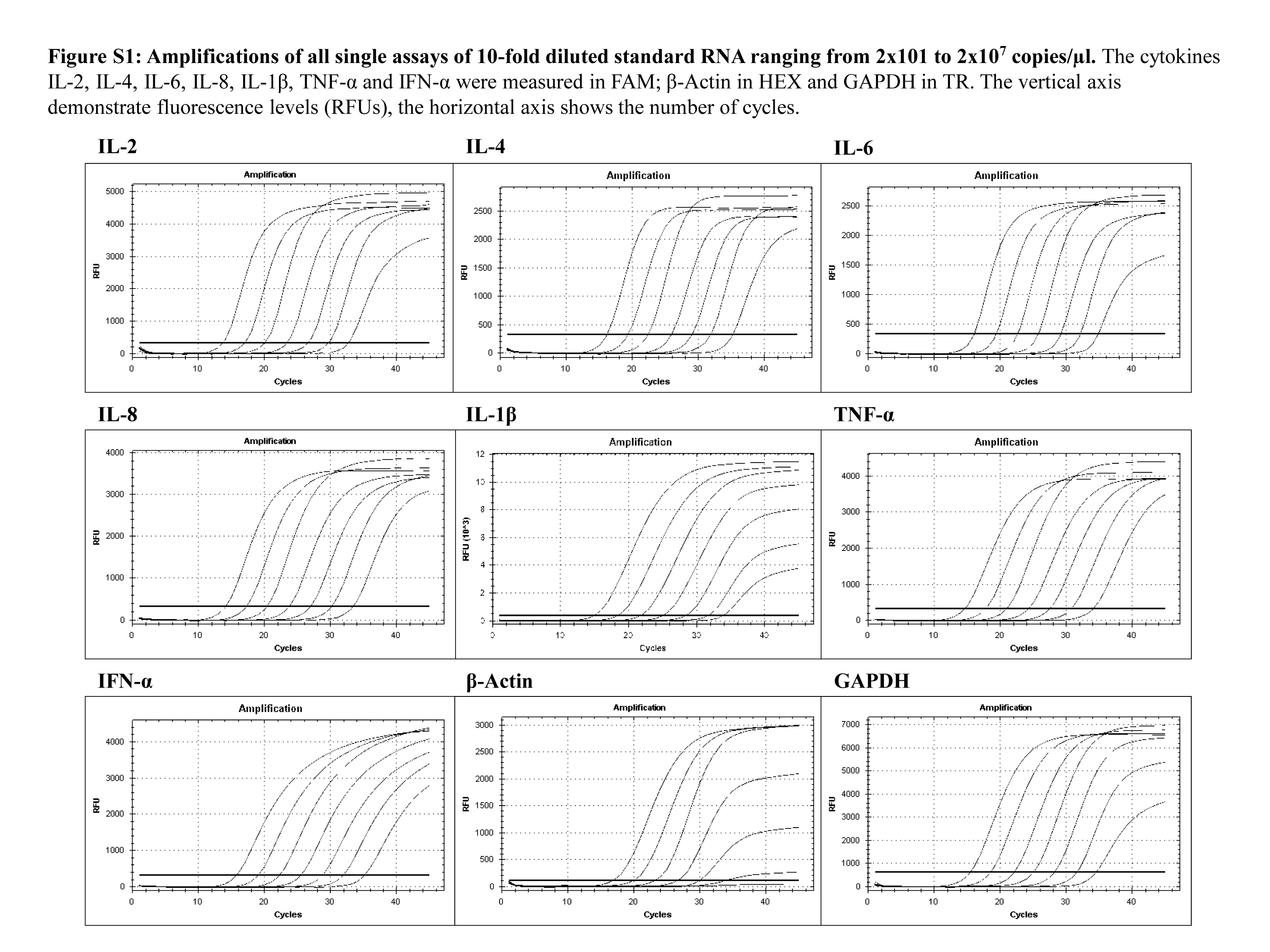

Supplement: Figure S1 — Amplifications of all single assays of 10-fold diluted standard RNA ranging from 2×101 to 2×10^7^ copies/µl. (TIF) [file pone.0108910.s001.tif]

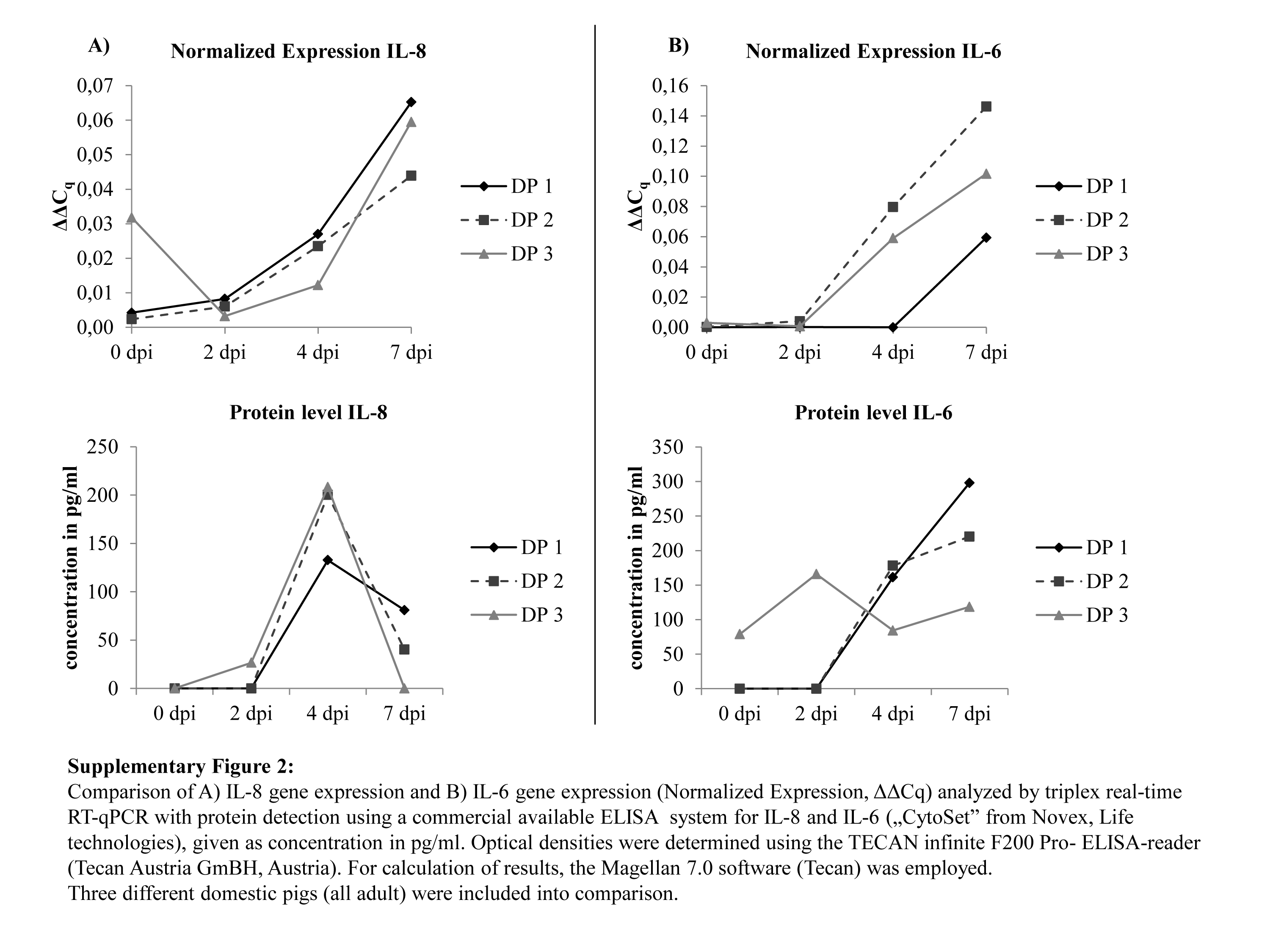

Supplement: Figure S2 — Exemplary comparison of gene expression and cytokine protein detection using ELISA systems (IL-6 and IL-8). (TIF) [file pone.0108910.s002.tif]
