# Supplementary material for: Development and Validation of a Harmonized TaqMan-Based Triplex Real-Time RT-PCR Protocol for the Quantitative Detection of Normalized Gene Expression Profiles of Seven Porcine Cytokines
Source: PLoS One. 2014 Sep 30;9(9):e108910. doi: 10.1371/journal.pone.0108910 (PMC4182501; doi:10.1371/journal.pone.0108910)
Supplement: Table S1 — RT-qPCR results of all single target assays. (DOC) [file pone.0108910.s003.doc]

**Table S1: RT-qPCR results of all single target assays.** Results were obtained including the 10-fold dilution series of the synthetic standard RNA and *in vitro* generated positive (pos.) RNA in all assays. The standard RNA dilution is given as copies/µl, ranging from 2x10^1^ to 2x10^7^*. In vitro* generated positive RNA obtained through specific stimulation of PBMCs is declared as “positive RNA” and given as 10-fold dilution steps ranging from 10^-1^ to 10^-7^. The “Limit of detection” describes the last dilution step with a positive RT-qPCR result.

| **Target gene** | **IL-2** | **IL-4** | **IL-6** | **IL-8** | **IL-1β** | **TNF-α** | **IFN-α** | **β-Actin** | **GAPDH** |
| --- | --- | --- | --- | --- | --- | --- | --- | --- | --- |
| **Efficiency in %** | 106.1 | 106.4 | 107.1 | 103.9 | 90.1 | 103.3 | 103.9 | 97.9 | 103.8 |
| **Standard RNA copies** | *Cq-values* | | | | | | | | |
| *2x10^1^* | 32.94 | 35.47 | 35.09 | 33.74 | 35.91 | 34.39 | 35.46 | N/A | 35.65 |
| *2x10^2^* | 30.00 | 32.15 | 32.32 | 30.44 | 33.46 | 31.07 | 32.24 | 33.46 | 31.88 |
| *2x10^3^* | 26.84 | 29.31 | 29.36 | 27.27 | 29.87 | 27.80 | 29.13 | 29.69 | 28.94 |
| *2x10^4^* | 23.59 | 26.20 | 26.01 | 24.07 | 25.98 | 24.57 | 25.82 | 26.36 | 25.41 |
| *2x10^5^* | 20.23 | 22.65 | 22.85 | 20.65 | 22.32 | 21.25 | 22.56 | 23.00 | 22.09 |
| *2x10^6^* | 17.24 | 19.60 | 19.58 | 17.53 | 18.45 | 18.10 | 19.35 | 19.70 | 18.97 |
| *2x10^7^* | 13.93 | 16.39 | 16.23 | 14.39 | 14.98 | 14.94 | 16.09 | 16.51 | 16.36 |
| **Limit of detection pos. RNA** | 10^-5^ | 10^-4^ | 10^-3^ | > 10^-7^ | 10^-6^ | 10^-4^ | 10^-3^ |  |  |
| **Pos. RNA dilution** | *Cq-values* | | | | | | |  |  |
| *10^-1^* | 22.85 | 30.27 | 29.07 | 21.13 | 23.29 | 28.81 | 31.75 |  |  |
| *10^-2^* | 26.26 | 33.19 | 32.21 | 24.42 | 27.1 | 32.27 | 35.27 |  |  |
| *10^-3^* | 29.61 | 36.19 | 34.83 | 27.83 | 30.15 | 36.17 | 39.05 |  |  |
| *10^-4^* | 32.95 | 40.14 | N/A | 31.1 | 32.84 | 39.92 | N/A |  |  |
| *10^-5^* | 36.2 | N/A | N/A | 34.25 | 35.07 | N/A | N/A |  |  |
| *10^-6^* | N/A | N/A | N/A | 37.09 | 39.09 | N/A | N/A |  |  |
| *10^-7^* | N/A | N/A | N/A | 38.15 | N/A | N/A | N/A |  |  |
