# Supplementary material for: Development and Validation of a Harmonized TaqMan-Based Triplex Real-Time RT-PCR Protocol for the Quantitative Detection of Normalized Gene Expression Profiles of Seven Porcine Cytokines
Source: PLoS One. 2014 Sep 30;9(9):e108910. doi: 10.1371/journal.pone.0108910 (PMC4182501; doi:10.1371/journal.pone.0108910)
Supplement: Table S2 — Comparison of single target and triplex RT-qPCR results. (DOC) [file pone.0108910.s004.doc]

**Table S2: Comparison of single target and triplex RT-qPCR results.** Cq-values and total amounts (given as copies/µl) of target cytokines (IL-2, IL-4, IL-6, IL-8, IL-1β, TNF-α and IFN-α) from the 10-fold diluted standard RNA series ranging from 2x10^2^ to 2x10^6^ copies/µl and of positive RNA dilutions of 10^-1^ to 10^-5^ are comparatively illustrated between single target and triplex assays (FAM). Corresponding deviations between Cq-values of single and multiplex assay as well as PCR-Efficiencies (E) and Baseline Thresholds (Thresholds) are additionally provided.

| **Dilutions** | **IL-2** |  |  |  |  | **IL-4** |  |  |  |  |
| --- | --- | --- | --- | --- | --- | --- | --- | --- | --- | --- |
|  | Single Target | Multiplex |  | Single Target | Multiplex | Single Target | Multiplex |  | Single Target | Multiplex |
|  | Cq-values | Cq-values | *Deviations* | Copies/µl | Copies/µl | Cq-values | Cq-values | *Deviations* | Copies/µl | Copies/µl |
| *PC RNAx10^-1^* | 22.71 | 23.14 | *-0.43* | 5.81E+04 | 6.94E+04 | 29.07 | 29.29 | *-0.02* | 1.42E+03 | 1.68E+03 |
| *PC RNAx10^-2^* | 26.21 | 26.41 | *-0.20* | 4.93E+03 | 6.97E+03 | 32.62 | 33.39 | *-0.77* | 1.12E+02 | 9.50E+01 |
| *PC RNAx10^-3^* | 29.44 | 29.78 | *-0.34* | 5.12E+02 | 6.46E+02 | 36.59 | 42.62 | *-6.03* | 6.53E+00 | 1.48E-01 |
| *PC RNAx10^-4^* | 32.64 | 33.05 | *-0.41* | 5.36E+01 | 6.48E+01 | N/A | N/A | */* | N/A | N/A |
| *PC RNAx10^-5^* | 35.99 | 37.80 | *-1.81* | 5.09E+00 | 2.29E+00 | N/A | N/A | */* | N/A | N/A |
| *Std 2x10^2^* | 30.83 | 31.45 | *-0.62* | 2.00E+02 | 2.00E+02 | 31.22 | 32.45 | *-1.23* | 2.00E+02 | 2.00E+02 |
| *Std 2x10^3^* | 27.47 | 28.21 | *-0.74* | 2.00E+03 | 2.00E+03 | 27.85 | 29.01 | *-1.16* | 2.00E+03 | 2.00E+03 |
| *Std 2x10^4^* | 24.15 | 24.80 | *-0.65* | 2.00E+04 | 2.00E+04 | 24.63 | 25.65 | *-1.02* | 2.00E+04 | 2.00E+04 |
| *Std 2x10^5^* | 20.93 | 21.77 | *-0.84* | 2.00E+05 | 2.00E+05 | 21.56 | 22.32 | *-0.76* | 2.00E+05 | 2.00E+05 |
| *Std 2x10^6^* | 17.73 | 18.32 | *-0.59* | 2.00E+06 | 2.00E+06 | 18.47 | 19.36 | *-0.89* | 2.00E+06 | 2.00E+06 |
| E in % | 102.1 % | 102.2 % |  |  |  | 104.5 % | 101.5 % |  |  |  |
| NTC | N/A | N/A |  |  |  | N/A | N/A |  |  |  |
| Threshold: | 300 | 300 |  |  |  | 300 | 300 |  |  |  |
|  | **IL-6** |  |  |  |  | **IL-8** |  |  |  |  |
|  | Single Target | Multiplex |  | Single Target | Multiplex | Single Target | Multiplex |  | Single Target | Multiplex |
|  | Cq-values | Cq-values | *Deviations* | Copies/µl | Copies/µl | Cq-values | Cq-values | *Deviations* | Copies/µl | Copies/µl |
| *PC RNAx10^-1^* | 27.33 | 26.74 | *0.59* | 3.53E+03 | 5.35E+03 | 19.38 | 19.56 | *-0.18* | 3.13E+05 | 3.14E+05 |
| *PC RNAx10^-2^* | 30.40 | 30.07 | *0.33* | 3.94E+02 | 4.94E+02 | 22.86 | 22.65 | *0.21* | 2.53E+04 | 3.40E+04 |
| *PC RNAx10^-3^* | 33.36 | 33.04 | *0.32* | 4.80E+01 | 5.96E+01 | 26.32 | 26.26 | *0.06* | 2.07E+03 | 2.53E+03 |
| *PC RNAx10^-4^* | 36.21 | 35.46 | *0.75* | 6.29E+00 | 1.06E+01 | 33.52 | 32.53 | *0.99* | 1.13E+01 | 2.78E+01 |
| *PC RNAx10^-5^* | N/A | N/A | */* | N/A | N/A | 34.66 | 33.98 | *0.68* | 4.97E+00 | 9.79E+00 |
| *Std 2x10^2^* | 31.27 | 31.26 | *0.01* | 2.00E+02 | 2.00E+02 | 29.52 | 29.88 | *-0.36* | 2.00E+02 | 2.00E+02 |
| *Std 2x10^3^* | 28.23 | 28.22 | *0.01* | 2.00E+03 | 2.00E+03 | 26.42 | 26.49 | *-0.07* | 2.00E+03 | 2.00E+03 |
| *Std 2x10^4^* | 24.92 | 24.88 | *0.04* | 2.00E+04 | 2.00E+04 | 23.21 | 23.37 | *-0.16* | 2.00E+04 | 2.00E+04 |
| *Std 2x10^5^* | 21.64 | 21.70 | *-0.06* | 2.00E+05 | 2.00E+05 | 19.9 | 20.14 | *-0.24* | 2.00E+05 | 2.00E+05 |
| *Std 2x10^6^* | 18.41 | 18.38 | *0.03* | 2.00E+06 | 2.00E+06 | 16.86 | 17.06 | *-0.20* | 2.00E+06 | 2.00E+06 |
| E in % | 104.0 % | 104.1 % |  |  |  | 106.1 % | 105.4 % |  |  |  |
| NTC | N/A | N/A |  |  |  | N/A | N/A |  |  |  |
| Threshold: | 300 | 300 |  |  |  | 300 | 300 |  |  |  |
|  | **IL-1β** |  |  |  |  | **TNF-α** |  |  |  |  |
|  | Single Target | Multiplex |  | Single Target | Multiplex | Single Target | Multiplex |  | Single Target | Multiplex |
|  | Cq-values | Cq-values | *Deviations* | Copies/µl | Copies/µl | Cq-values | Cq-values | *Deviations* | Copies/µl | Copies/µl |
| *PC RNAx10^-1^* | 25.54 | 26.45 | *-0.91* | 1.12E+04 | 1.94E+04 | 27.72 | 26.96 | *0.76* | 1.23E+03 | 1.26E+03 |
| *PC RNAx10^-2^* | 28.79 | 29.85 | *-1.06* | 1.08E+03 | 1.70E+03 | 30.88 | 31.19 | *-0.31* | 1.35E+02 | 6.72E+01 |
| *PC RNAx10^-3^* | 31.78 | 34.27 | *-2.49* | 1.24E+02 | 7.19E+01 | 35.55 | 36.81 | *-1.26* | 6.18E+00 | 1.38E+00 |
| *PC RNAx10^-4^* | N/A | N/A | */* | N/A | N/A | N/A | N/A | */* | 2.30E-02 | N/A |
| *PC RNAx10^-5^* | 40.34 | N/A | *-4.66* | 2.58E-01 | N/A | N/A | N/A | */* | N/A | N/A |
| *Std 2x10^2^* | 30.91 | 33.32 | *-2.41* | 2.00E+02 | 2.00E+02 | 30.38 | 29.87 | *0.51* | 2.00E+02 | 2.00E+02 |
| *Std 2x10^3^* | 28.13 | 29.34 | *-1.10* | 2.00E+03 | 2.00E+03 | 26.97 | 26.06 | *0.91* | 2.00E+03 | 2.00E+03 |
| *Std 2x10^4^* | 24.90 | 25.94 | *-1.04* | 2.00E+04 | 2.00E+04 | 23.55 | 22.80 | *0.75* | 2.00E+04 | 2.00E+04 |
| *Std 2x10^5^* | 21.47 | 23.03 | *-1.56* | 2.00E+05 | 2.00E+05 | 20.3 | 19.62 | *0.68* | 2.00E+05 | 2.00E+05 |
| *Std 2x10^6^* | 18.28 | 20.40 | *-2.12* | 2.00E+06 | 2.00E+06 | 17.16 | 16.46 | *0.70* | 2.00E+06 | 2.00E+06 |
| E in % | 105.7 % | 104.6 % |  |  |  | 100.4 % | 99.8 % |  |  |  |
| NTC | N/A | N/A |  |  |  | N/A | N/A |  |  |  |
| Threshold: | 250 | 450 |  |  |  | 350 | 150 |  |  |  |
|  | **IFN-α** |  |  |  |  |  | | | | |
|  | Single Target | Multiplex |  | Single Target | Multiplex |  | | | | |
|  | Cq-values | Cq-values | *Deviations* | Copies/µl | Copies/µl |  | | | | |
| *PC RNA 10^-1^* | 26.66 | 25.05 | *1.61* | 1.11E+03 | 3.50E+03 |  | | | | |
| *PC RNA 10^-2^* | 30.02 | 30.16 | *-0.14* | 1.16E+02 | 1.06E+02 |  | | | | |
| *PC RNA 10^-3^* | 32.46 | N/A | *-12.54* | 2.26E+01 | N/A |  | | | | |
| *PC RNA 10^-4^* | N/A | N/A | */* | N/A | N/A |  | | | | |
| *PC RNA 10^-5^* | 43.87 | N/A | *-1.13* | 1.05E-02 | N/A |  | | | | |
| *Std 2x10^2^* | 28.97 | 28.98 | *-0.01* | 2.00E+02 | 2.00E+02 |  | | | | |
| *Std 2x10^3^* | 26.08 | 25.78 | *0.30* | 2.00E+03 | 2.00E+03 |  | | | | |
| *Std 2x10^4^* | 22.35 | 23.15 | *-0.80* | 2.00E+04 | 2.00E+04 |  | | | | |
| *Std 2x10^5^* | 19.09 | 19.13 | *-0.04* | 2.00E+05 | 2.00E+05 |  | | | | |
| *Std 2x10^6^* | 15.34 | 15.46 | *-0.12* | 2.00E+06 | 2.00E+06 |  | | | | |
| E in % | 95.9 % | 98.0 % |  |  |  |  | | | | |
| NTC | N/A | N/A |  |  |  |  | | | | |
| Threshold: | 150 | 150 |  |  |  |  | | | | |

PC RNA= *in vitro* generated positive RNA; Std=Standard; E= Efficiency of RT-qPCR; NTC= H_2_O used as negative control; N/A=no Cq-value detectable
