# Supplementary material for: Development and Validation of a Harmonized TaqMan-Based Triplex Real-Time RT-PCR Protocol for the Quantitative Detection of Normalized Gene Expression Profiles of Seven Porcine Cytokines
Source: PLoS One. 2014 Sep 30;9(9):e108910. doi: 10.1371/journal.pone.0108910 (PMC4182501; doi:10.1371/journal.pone.0108910)
Supplement: Table S3 — Comparison of RT-qPCRs between a total mastermix reaction volume of 25 µl and the halved volume of 12.5 µl. (DOC) [file pone.0108910.s005.doc]

**Table S3: Comparison of RT-qPCRs between a total mastermix reaction volume of 25 µl and the halved volume of 12.5 µl.**

10-fold dilution series of synthetic standard RNA (“std. dilutions”) ranging from 2x10^1^ to 2x10^6^ copies/µl and *in vitro* generated positive RNA (“pos. RNA”) including the dilutions steps 10^-1^ to 10^-5^ were used for assessment of the applicability of a halved mastermix reaction volume of 12.5 µl instead of 25 µl. Cq-values, total amounts (in copies/µl) as well as end fluorescence levels (End RFUs) for each triplex assay are comparatively provided for each channel (target cytokines IL-2, IL-4, IL-6, IL-8, IL-1β, TNF-α , IFN-α in FAM; β-Actin in Hex; GAPDH in Texas Red). Fat letters indicate deviations of more than three Cq-values.

Std=Standard; PC RNA=*in vitro* generated positive RNA; N/A=no Cq-value detectable; V-MM=total mastermix reaction volume including RNA-template

| **IL-2 triplex assay** | | | | | | | | | | | | | | | | | | |
| --- | --- | --- | --- | --- | --- | --- | --- | --- | --- | --- | --- | --- | --- | --- | --- | --- | --- | --- |
| ***Pos. RNA and std. dilutions***  **V-MM in µl** | **IL-2 *FAM*** | | | | | | **β-Actin *HEX*** | | | | | | **GAPDH *Texas Red*** | | | | | |
|  | **Cq-values** | | **Copies/µl** | | **End RFUs** | | **Cq-values** | | **Copies/µl** | | **End RFUs** | | **Cq-values** | | **Copies/µl** | | **End RFUs** | |
|  | **25** | **12.5** | **25** | **12.5** | **25** | **12.5** | **25** | **12.5** | **25** | **12.5** | **25** | **12.5** | **25** | **12.5** | **25** | **12.5** | **25** | **12.5** |
| ***PC RNA 10^-1^*** | 21.30 | 21.31 | 5.49E+04 | 7.23E+04 | 7075 | 5966 | 21.16 | 21.56 | 2.86E+05 | 3.21E+05 | 5014 | 3397 | 20.64 | 21.04 | 6.24E+05 | 7.50E+05 | 10413 | 6370 |
| ***PC RNA 10^-2^*** | 24.72 | 25.01 | 4.56E+03 | 5.06E+03 | 5856 | 4286 | 24.37 | 25.07 | 3.13E+04 | 2.88E+04 | 4196 | 3171 | 23.78 | 24.4 | 7.19E+04 | 7.26E+04 | 9158 | 6208 |
| ***PC RNA 10^-3^*** | 28.03 | 28.11 | 4.14E+02 | 5.42E+02 | 3523 | 2259 | 27.57 | 27.83 | 3.47E+03 | 4.33E+03 | 2685 | 1911 | 27.1 | 27.57 | 7.30E+03 | 7.97E+03 | 6710 | 4019 |
| ***PC RNA 10^-4^*** | 31.46 | 31.87 | 3.42E+01 | 3.62E+01 | 844 | 572 | 30.54 | 31.53 | 4.49E+02 | 3.41E+02 | 1029 | 691 | 30.14 | 30.82 | 9.03E+02 | 8.31E+02 | 3432 | 2131 |
| ***PC RNA 10^-5^*** | N/A | N/A | N/A | N/A | 33.2 | 21.1 | **35.9** | **N/A** | 1.13E+01 | N/A | 196 | 70.4 | 36.98 | 38.33 | 8.14E+00 | 4.45E+00 | 415 | 302 |
| ***Std 2x10^1^*** | **32.49** | **40.68** | 2.00E+01 | 2.00E+01 | 551 | 229 | 37.54 | 34.34 | 2.00E+01 | 2.00E+01 | 143 | 167 | **36.09** | **N/A** | 2.00E+01 | 2.00E+01 | 482 | 23.5 |
| ***Std 2x10^2^*** | 28.81 | 29.48 | 2.00E+02 | 2.00E+02 | 2652 | 1465 | 31.65 | 32.28 | 2.00E+02 | 2.00E+02 | 628 | 455 | 31.83 | 32.96 | 2.00E+02 | 2.00E+02 | 1835 | 909 |
| ***Std 2x10^3^*** | 26.06 | 26.27 | 2.00E+03 | 2.00E+03 | 5305 | 3528 | 28.51 | 29.03 | 2.00E+03 | 2.00E+03 | 1863 | 1268 | 29.08 | 29.44 | 2.00E+03 | 2.00E+03 | 4075 | 2463 |
| ***Std 2x10^4^*** | 22.88 | 23.20 | 2.00E+04 | 2.00E+04 | 6891 | 4694 | 25.05 | 25.53 | 2.00E+04 | 2.00E+04 | 3554 | 2432 | 25.5 | 26.17 | 2.00E+04 | 2.00E+04 | 7014 | 4273 |
| ***Std 2x10^5^*** | 19.39 | 19.87 | 2.00E+05 | 2.00E+05 | 7071 | 5022 | 21.51 | 22.21 | 2.00E+05 | 2.00E+05 | 4387 | 3220 | 22.24 | 23.05 | 2.00E+05 | 2.00E+05 | 8443 | 5391 |
| ***Std 2x10^6^*** | 16.3 | 16.69 | 2.00E+06 | 2.00E+06 | 7294 | 5104 | 18.41 | 18.93 | 2.00E+06 | 2.00E+06 | 4819 | 3570 | 19.14 | 19.62 | 2.00E+06 | 2.00E+06 | 9243 | 6001 |
| **IL-4 triplex assay** | | | | | | | | | | | | | | | | | | |
| ***Pos. RNA and std. dilutions***  **V-MM in µl** | **IL-4 *FAM*** | | | | | | **β-Actin *HEX*** | | | | | | **GAPDH *Texas Red*** | | | | | |
|  | **Cq-values** | | **Copies/µl** | | **End RFUs** | | **Cq-values** | | **Copies/µl** | | **End RFUs** | | **Cq-values** | | **Copies/µl** | | **End RFUs** | |
|  | **25** | **12.5** | **25** | **12.5** | **25** | **12.5** | **25** | **12.5** | **25** | **12.5** | **25** | **12.5** | **25** | **12.5** | **25** | **12.5** | **25** | **12.5** |
| ***PC RNA 10^-1^*** | 27.66 | 28.20 | 2.40E+03 | 2.24E+03 | 9498 | 6133 | 21.76 | 23.00 | 3.19E+05 | 2.55E+05 | 4306 | 2399 | 20.41 | 21.06 | 5.89E+05 | 5.65E+05 | 9186 | 5964 |
| ***PC RNA 10^-2^*** | 31.09 | 31.57 | 2.08E+02 | 2.08E+02 | 5714 | 3765 | 25.22 | 26.17 | 2.86E+04 | 3.32E+04 | 3532 | 2025 | 23.85 | 24.23 | 5.86E+04 | 6.10E+04 | 8205 | 5604 |
| ***PC RNA 10^-3^*** | 36.28 | 36.95 | 5.10E+00 | 5.10E+00 | 1382 | 1211 | 28.36 | 29.36 | 3.21E+03 | 4.28E+03 | 2403 | 1413 | 26.99 | 27.38 | 7.15E+03 | 6.68E+03 | 6491 | 4891 |
| ***PC RNA 10^-4^*** | 44.79 | N/A | 1.19E-02 | N/A | 309 | 27.9 | 31.26 | 32.45 | 4.24E+02 | 5.86E+02 | 1101 | 621 | 31.00 | 30.59 | 4.88E+02 | 6.94E+02 | 3092 | 2746 |
| ***PC RNA 10^-5^*** | N/A | N/A | N/A | N/A | 26.6 | 23 | **36.37** | **N/A** | 1.20E+01 | N/A | 267 | 124 | 33.42 | 32.54 | 9.62E+01 | 1.77E+02 | 1503 | 1641 |
| ***Std 2x10^1^*** | 34.82 | 34.85 | 2.00E+01 | 2.00E+01 | 2507 | 2083 | 44.87 | N/A | 2.00E+01 | 2.00E+01 | 142 | 140 | 39.66 | 38.13 | 2.00E+01 | 2.00E+01 | 310 | 375 |
| ***Std 2x10^2^*** | 31.08 | 31.73 | 2.00E+02 | 2.00E+02 | 6475 | 4015 | 32.29 | 34.58 | 2.00E+02 | 2.00E+02 | 659 | 317 | 32.59 | 32.35 | 2.00E+02 | 2.00E+02 | 1693 | 1362 |
| ***Std 2x10^3^*** | 27.98 | 28.21 | 2.00E+03 | 2.00E+03 | 10606 | 7445 | 29.13 | 30.08 | 2.00E+03 | 2.00E+03 | 1845 | 1056 | 28.68 | 29.08 | 2.00E+03 | 2.00E+03 | 4635 | 3361 |
| ***Std 2x10^4^*** | 24.65 | 25.12 | 2.00E+04 | 2.00E+04 | 13346 | 8699 | 25.6 | 26.81 | 2.00E+04 | 2.00E+04 | 3531 | 1945 | 25.18 | 25.86 | 2.00E+04 | 2.00E+04 | 7807 | 5079 |
| ***Std 2x10^5^*** | 21.58 | 21.83 | 2.00E+05 | 2.00E+05 | 13488 | 9773 | 22.63 | 23.24 | 2.00E+05 | 2.00E+05 | 4264 | 2791 | 22.16 | 22.52 | 2.00E+05 | 2.00E+05 | 9061 | 6704 |
| ***Std 2x10^6^*** | 18.14 | 18.62 | 2.00E+06 | 2.00E+06 | 13527 | 9775 | 19.02 | 20.09 | 2.00E+06 | 2.00E+06 | 4686 | 3046 | 18.67 | 19.26 | 2.00E+06 | 2.00E+06 | 9543 | 7334 |
| **IL-6 triplex assay** | | | | | | | | | | | | | | | | | | |
| ***Pos. RNA and std. dilutions***  **V-MM in µl** | **IL-6 *FAM*** | | | | | | **β-Actin *HEX*** | | | | | | **GAPDH *Texas Red*** | | | | | |
|  | **Cq-values** | | **Copies/µl** | | **End RFUs** | | **Cq-values** | | **Copies/µl** | | **End RFUs** | | **Cq-values** | | **Copies/µl** | | **End RFUs** | |
|  | **25** | **12.5** | **25** | **12.5** | **25** | **12.5** | **25** | **12.5** | **25** | **12.5** | **25** | **12.5** | **25** | **12.5** | **25** | **12.5** | **25** | **12.5** |
| ***PC RNA 10^-1^*** | 25.96 | 26.32 | 5.41E+03 | 5.69E+03 | 7286 | 6193 | 21.83 | 22.27 | 2.62E+05 | 2.79E+05 | 3645 | 3020 | 20.89 | 21.25 | 5.27E+05 | 7.01E+05 | 7334 | 5988 |
| ***PC RNA 10^-2^*** | 29.26 | 29.74 | 4.67E+02 | 4.66E+02 | 5613 | 4929 | 25.20 | 25.98 | 2.81E+04 | 2.29E+04 | 3124 | 2603 | 24.22 | 24.78 | 5.38E+04 | 6.00E+04 | 6897 | 5614 |
| ***PC RNA 10^-3^*** | 32.88 | 33.51 | 3.16E+01 | 2.95E+01 | 2507 | 2218 | 28.38 | 28.96 | 3.42E+03 | 3.06E+03 | 2145 | 1859 | 27.36 | 27.94 | 6.25E+03 | 6.68E+03 | 5597 | 4620 |
| ***PC RNA 10^-4^*** | 42.02 | 41.59 | 3.57E-02 | 7.93E-02 | 445 | 466 | 31.42 | 32.19 | 4.59E+02 | 3.46E+02 | 985 | 812 | 30.64 | 31.09 | 6.62E+02 | 7.43E+02 | 3345 | 2781 |
| ***PC RNA 10^-5^*** | N/A | N/A | N/A | N/A | 10.5 | 7.35 | 36.02 | 38.2 | 2.17E+01 | 6.01E+00 | 278 | 221 | 34.59 | 34.69 | 4.41E+01 | 6.07E+01 | 1068 | 1047 |
| ***Std 2x10^1^*** | 33.37 | 35.16 | 2.00E+01 | 2.00E+01 | 2366 | 1545 | N/A | N/A | 2.00E+01 | 2.00E+01 | 111 | 102 | **36.66** | **N/A** | 2.00E+01 | 2.00E+01 | 609 | 200 |
| ***Std 2x10^2^*** | 30.26 | 30.62 | 2.00E+02 | 2.00E+02 | 5476 | 4920 | 32.84 | 32.98 | 2.00E+02 | 2.00E+02 | 602 | 569 | 32.41 | 32.86 | 2.00E+02 | 2.00E+02 | 1820 | 1497 |
| ***Std 2x10^3^*** | 27.58 | 28.04 | 2.00E+03 | 2.00E+03 | 7324 | 6791 | 29.02 | 29.71 | 2.00E+03 | 2.00E+03 | 1601 | 1340 | 29.09 | 29.92 | 2.00E+03 | 2.00E+03 | 3668 | 2832 |
| ***Std 2x10^4^*** | 24.34 | 24.69 | 2.00E+04 | 2.00E+04 | 7714 | 8032 | 25.65 | 26.09 | 2.00E+04 | 2.00E+04 | 2390 | 2390 | 25.52 | 26.20 | 2.00E+04 | 2.00E+04 | 4981 | 4670 |
| ***Std 2x10^5^*** | 21.16 | 21.47 | 2.00E+05 | 2.00E+05 | 9291 | 8358 | 22.22 | 22.70 | 2.00E+05 | 2.00E+05 | 3313 | 2918 | 22.3 | 23.10 | 2.00E+05 | 2.00E+05 | 6635 | 5459 |
| ***Std 2x10^6^*** | 17.78 | 18.18 | 2.00E+06 | 2.00E+06 | 9202 | 8472 | 18.85 | 19.42 | 2.00E+06 | 2.00E+06 | 3546 | 3180 | 19.01 | 19.74 | 2.00E+06 | 2.00E+06 | 6966 | 5926 |
| **IL-8 triplex assay** | | | | | | | | | | | | | | | | | | |
| ***Pos. RNA and std. dilutions***  **V-MM in µl** | **IL-8 *FAM*** | | | | | | **β-Actin *HEX*** | | | | | | **GAPDH *Texas Red*** | | | | | |
|  | **Cq-values** | | **Copies/µl** | | **End RFUs** | | **Cq-values** | | **Copies/µl** | | **End RFUs** | | **Cq-values** | | **Copies/µl** | | **End RFUs** | |
|  | **25** | **12.5** | **25** | **12.5** | **25** | **12.5** | **25** | **12.5** | **25** | **12.5** | **25** | **12.5** | **25** | **12.5** | **25** | **12.5** | **25** | **12.5** |
| ***PC RNA 10^-1^*** | 19.24 | 19.80 | 2.67E+05 | 2.68E+05 | 10266 | 8673 | 26.38 | 26.19 | 2.54E+04 | 2.12E+04 | 2400 | 2250 | 24.67 | 24.99 | 5.73E+04 | 5.56E+04 | 5079 | 4673 |
| ***PC RNA 10^-2^*** | 22.61 | 23.10 | 2.42E+04 | 2.68E+04 | 10114 | 8570 | 30.68 | 29.67 | 1.70E+03 | 1.87E+03 | 1326 | 1361 | 28.45 | 28.42 | 4.47E+03 | 5.24E+03 | 3866 | 3470 |
| ***PC RNA 10^-3^*** | 26.16 | 26.49 | 1.95E+03 | 2.51E+03 | 9416 | 8001 | 34.90 | 33.54 | 1.19E+02 | 1.27E+02 | 458 | 522 | 32.07 | 32.16 | 3.91E+02 | 3.99E+02 | 2190 | 1759 |
| ***PC RNA 10^-4^*** | 30.31 | 30.80 | 1.02E+02 | 1.25E+02 | 6355 | 4637 | N/A | N/A | N/A | N/A | 10.1 | 5.26 | 43.21 | N/A | 2.13E-01 | N/A | 248 | 4.58 |
| ***PC RNA 10^-5^*** | 32.32 | 33.08 | 2.44E+01 | 2.54E+01 | 3397 | 2226 | N/A | N/A | N/A | N/A | 3.65 | 62.6 | 39.37 | 41.11 | 2.85E+00 | 8.44E-01 | 382 | 302 |
| ***Std 2x10^1^*** | **32.25** | **36.55** | 2.00E+01 | 2.00E+01 | 3568 | 1016 | 41.61 | 43.00 | 2.00E+01 | 2.00E+01 | 159 | 152 | **38.18** | **N/A** | 2.00E+01 | 2.00E+01 | 460 | 208 |
| ***Std 2x10^2^*** | 29.66 | 30.07 | 2.00E+02 | 2.00E+02 | 6818 | 5307 | 34.41 | 32.74 | 2.00E+02 | 2.00E+02 | 472 | 653 | 33.06 | 33.17 | 2.00E+02 | 2.00E+02 | 1677 | 1364 |
| ***Std 2x10^3^*** | 26.27 | 26.92 | 2.00E+03 | 2.00E+03 | 9762 | 8296 | 30.03 | 29.82 | 2.00E+03 | 2.00E+03 | 1571 | 1465 | 29.63 | 29.90 | 2.00E+03 | 2.00E+03 | 3591 | 3081 |
| ***Std 2x10^4^*** | 22.96 | 23.54 | 2.00E+04 | 2.00E+04 | 10497 | 9354 | 26.63 | 26.31 | 2.00E+04 | 2.00E+04 | 2520 | 2571 | 26.23 | 26.41 | 2.00E+04 | 2.00E+04 | 5122 | 4870 |
| ***Std 2x10^5^*** | 19.57 | 20.09 | 2.00E+05 | 2.00E+05 | 11554 | 11194 | 23.13 | 22.77 | 2.00E+05 | 2.00E+05 | 3248 | 3616 | 22.86 | 23.02 | 2.00E+05 | 2.00E+05 | 6253 | 6762 |
| ***Std 2x10^6^*** | 16.29 | 16.99 | 2.00E+06 | 2.00E+06 | 11002 | 10190 | 19.58 | 19.72 | 2.00E+06 | 2.00E+06 | 3303 | 3571 | 19.37 | 19.88 | 2.00E+06 | 2.00E+06 | 6352 | 6599 |
| **IL-1β triplex assay** | | | | | | | | | | | | | | | | | | |
| ***Pos. RNA and std. dilutions***  **V-MM in µl** | **IL-1β *FAM*** | | | | | | **β-Actin *HEX*** | | | | | | **GAPDH *Texas Red*** | | | | | |
|  | **Cq-values** | | **Copies/µl** | | **End RFUs** | | **Cq-values** | | **Copies/µl** | | **End RFUs** | | **Cq-values** | | **Copies/µl** | | **End RFUs** | |
|  | **25** | **12.5** | **25** | **12.5** | **25** | **12.5** | **25** | **12.5** | **25** | **12.5** | **25** | **12.5** | **25** | **12.5** | **25** | **12.5** | **25** | **12.5** |
| ***PC RNA 10^-1^*** | 24.07 | 24.83 | 3.84E+05 | 2.97E+05 | 1896 | 1577 | 27.07 | 27.72 | 1.68E+04 | 1.16E+04 | 4159 | 2990 | 26.71 | 27.76 | 3.33E+04 | 1.51E+04 | 7785 | 5294 |
| ***PC RNA 10^-2^*** | 27.40 | 27.54 | 5.66E+04 | 6.94E+04 | 1133 | 959 | 30.51 | 30.65 | 1.54E+03 | 1.37E+03 | 2502 | 1889 | 30.12 | 30.63 | 3.08E+03 | 1.80E+03 | 5471 | 3859 |
| ***PC RNA 10^-3^*** | 30.97 | 31.55 | 7.31E+03 | 8.10E+03 | 406 | 326 | 33.64 | 33.37 | 1.75E+02 | 1.88E+02 | 897 | 807 | 33.32 | 35.62 | 3.30E+02 | 4.50E+01 | 2481 | 1027 |
| ***PC RNA 10^-4^*** | N/A | N/A | N/A | N/A | 12.9 | 12.2 | N/A | N/A | N/A | 2.21E+02 | 86.8 | 823 | N/A | N/A | N/A | N/A | -43.4 | 4.29 |
| ***PC RNA 10^-5^*** | N/A | N/A | N/A | N/A | 10.5 | 7.89 | N/A | N/A | N/A | 1.40E+02 | 3.55 | 643 | N/A | N/A | N/A | N/A | -61.5 | 5.23 |
| ***Std 2x10^1^*** | N/A | N/A | 2.00E+01 | 2.00E+01 | 11.9 | 11.6 | **39.50** | **35.83** | 2.00E+01 | 2.00E+01 | 208 | 386 | **N/A** | **37.98** | 2.00E+01 | 2.00E+01 | 5.42 | 709 |
| ***Std 2x10^2^*** | N/A | N/A | 2.00E+02 | 2.00E+02 | 30.5 | 26.7 | 33.33 | 33.17 | 2.00E+02 | 2.00E+02 | 1005 | 925 | 34.08 | 33.52 | 2.00E+02 | 2.00E+02 | 2276 | 2229 |
| ***Std 2x10^3^*** | 33.49 | 34.78 | 2.00E+03 | 2.00E+03 | 257 | 185 | 30.18 | 30.17 | 2.00E+03 | 2.00E+03 | 2838 | 2176 | 30.62 | 30.54 | 2.00E+03 | 2.00E+03 | 5306 | 4428 |
| ***Std 2x10^4^*** | 28.87 | 29.14 | 2.00E+04 | 2.00E+04 | 925 | 670 | 27.01 | 27.10 | 2.00E+04 | 2.00E+04 | 4562 | 3354 | 27.52 | 27.49 | 2.00E+04 | 2.00E+04 | 7687 | 6117 |
| ***Std 2x10^5^*** | 25.11 | 25.15 | 2.00E+05 | 2.00E+05 | 1693 | 1444 | 23.46 | 23.85 | 2.00E+05 | 2.00E+05 | 5503 | 4557 | 24.16 | 24.23 | 2.00E+05 | 2.00E+05 | 8765 | 7970 |
| ***Std 2x10^6^*** | 21.38 | 21.78 | 2.00E+06 | 2.00E+06 | 2138 | 1637 | 20.12 | 20.52 | 2.00E+06 | 2.00E+06 | 5816 | 4394 | 20.84 | 21.10 | 2.00E+06 | 2.00E+06 | 9275 | 7427 |
| **TNF-α triplex assay** | | | | | | | | | | | | | | | | | | |
| ***Pos. RNA and std. dilutions***  **V-MM in µl** | **TNF-α *FAM*** | | | | | | **β-Actin *HEX*** | | | | | | **GAPDH *Texas Red*** | | | | | |
|  | **Cq-values** | | **Copies/µl** | | **End RFUs** | | **Cq-values** | | **Copies/µl** | | **End RFUs** | | **Cq-values** | | **Copies/µl** | | **End RFUs** | |
|  | **25** | **12.5** | **25** | **12.5** | **25** | **12.5** | **25** | **12.5** | **25** | **12.5** | **25** | **12.5** | **25** | **12.5** | **25** | **12.5** | **25** | **12.5** |
| ***PC RNA 10^-1^*** | 27.16 | 27.33 | 1.29E+03 | 1.13E+03 | 4057 | 3296 | 26.38 | 27.05 | 1.04E+04 | 1.05E+04 | 4248 | 2486 | 26.13 | 26.03 | 2.77E+04 | 2.70E+04 | 8996 | 6823 |
| ***PC RNA 10^-2^*** | 30.57 | 30.71 | 1.21E+02 | 1.05E+02 | 1856 | 1692 | 29.75 | 29.80 | 8.80E+02 | 1.41E+03 | 2447 | 1482 | 29.47 | 29.24 | 2.53E+03 | 2.82E+03 | 6342 | 5056 |
| ***PC RNA 10^-3^*** | **42.03** | **38.82** | 4.19E-02 | 3.49E-01 | 325 | 532 | 31.07 | 32.88 | 3.31E+02 | 1.48E+02 | 993 | 548 | 33.27 | 32.80 | 1.66E+02 | 2.32E+02 | 2883 | 2562 |
| ***PC RNA 10^-4^*** | N/A Cq | N/A | N/A | N/A | -6.66 | 3.38 | **30.29** | **34.57** | 1.56E+01 | 4.30E+01 | 621 | 309 | **39.57** | **N/A** | 1.82E+00 | N/A | 680 | 5 |
| ***PC RNA 10^-5^*** | N/A Cq | N/A | N/A | N/A | 4.14 | 4.58 | 34.24 | 34.51 | 3.22E+01 | 4.49E+01 | 483 | 293 | N/A | N/A | N/A | N/A | 2.26 | 1.86 |
| ***Std 2x10^1^*** | 36.74 | 34.36 | 2.00E+01 | 2.00E+01 | 614 | 961 | 33.99 | 32.97 | 2.00E+01 | 2.00E+01 | 619 | 439 | **38.93** | **43.27** | 2.00E+01 | 2.00E+01 | 734 | 388 |
| ***Std 2x10^2^*** | 30.00 | 29.96 | 2.00E+02 | 2.00E+02 | 2372 | 2113 | 31.53 | 32.25 | 2.00E+02 | 2.00E+02 | 1319 | 602 | 33.01 | 33.21 | 2.00E+02 | 2.00E+02 | 2905 | 2153 |
| ***Std 2x10^3^*** | 26.35 | 26.35 | 2.00E+03 | 2.00E+03 | 5488 | 4615 | 28.78 | 29.55 | 2.00E+03 | 2.00E+03 | 2920 | 1666 | 29.79 | 29.53 | 2.00E+03 | 2.00E+03 | 5799 | 4812 |
| ***Std 2x10^4^*** | 23.15 | 23.18 | 2.00E+04 | 2.00E+04 | 8864 | 6514 | 25.72 | 26.28 | 2.00E+04 | 2.00E+04 | 4693 | 2698 | 26.59 | 26.35 | 2.00E+04 | 2.00E+04 | 8735 | 6459 |
| ***Std 2x10^5^*** | 19.92 | 20.02 | 2.00E+05 | 2.00E+05 | 9890 | 7939 | 22.37 | 23.01 | 2.00E+05 | 2.00E+05 | 5370 | 3421 | 23.38 | 23.19 | 2.00E+05 | 2.00E+05 | 9375 | 7516 |
| ***Std 2x10^6^*** | 16.64 | 16.76 | 2.00E+06 | 2.00E+06 | 10544 | 7974 | 19.08 | 19.78 | 2.00E+06 | 2.00E+06 | 5705 | 3403 | 20.14 | 20.01 | 2.00E+06 | 2.00E+06 | 9841 | 7365 |
| **IFN-α triplex assay** | | | | | | | | | | | | | | | | | | |
| ***Pos. RNA and std. dilutions***  **V-MM in µl** | **IFN-α *FAM*** | | | | | | **β-Actin *HEX*** | | | | | | **GAPDH *Texas Red*** | | | | | |
|  | **Cq-values** | | **Copies/µl** | | **End RFUs** | | **Cq-values** | | **Copies/µl** | | **End RFUs** | | **Cq-values** | | **Copies/µl** | | **End RFUs** | |
|  | **25** | **12.5** | **25** | **12.5** | **25** | **12.5** | **25** | **12.5** | **25** | **12.5** | **25** | **12.5** | **25** | **12.5** | **25** | **12.5** | **25** | **12.5** |
| ***PC RNA 10^-1^*** | 28.70 | 29.12 | 3.86E+02 | 3.28E+02 | 5112 | 3778 | 26.40 | 26.77 | 1.62E+04 | 9.03E+03 | 3224 | 2521 | 25.22 | 26.05 | 3.31E+04 | 2.84E+04 | 10948 | 5972 |
| ***PC RNA 10^-2^*** | 33.33 | 35.69 | 1.26E+01 | 2.87E+00 | 866 | 518 | 29.65 | 29.43 | 2.63E+03 | 1.44E+03 | 1287 | 1325 | 28.64 | 29.24 | 2.71E+03 | 2.94E+03 | 6050 | 3819 |
| ***PC RNA 10^-3^*** | **40.28** | **N/A** | 7.47E-02 | N/A | 263 | 92.8 | 33.80 | 34.89 | 2.55E+02 | 3.36E+01 | 335 | 249 | 31.28 | 32.79 | 3.94E+02 | 2.35E+02 | 3407 | 1591 |
| ***PC RNA 10^-4^*** | N/A | N/A | N/A | N/A | 10.6 | 6.42 | N/A | N/A | N/A | N/A | 9.86 | 39 | 37.03 | 38.02 | 5.91E+00 | 5.74E+00 | 573 | 412 |
| ***PC RNA 10^-5^*** | N/A | N/A | N/A | N/A | 120 | 3.06 | N/A | N/A | N/A | N/A | 1.01 | 0.8 | 42.08 | 42.51 | 1.47E-01 | 2.38E-01 | 222 | 209 |
| ***Std 2x10^1^*** | 32.95 | 33.36 | 2.00E+01 | 2.00E+01 | 1101 | 944 | **41.20** | **N/A** | 2.00E+01 | 2.00E+01 | 44.9 | 34.9 | **34.91** | **N/A** | 2.00E+01 | 2.00E+01 | 1035 | 4.79 |
| ***Std 2x10^2^*** | 29.29 | 29.42 | 2.00E+02 | 2.00E+02 | 5006 | 4057 | 32.08 | 32.37 | 2.00E+02 | 2.00E+02 | 587 | 528 | 32.55 | 34.92 | 2.00E+02 | 2.00E+02 | 2275 | 822 |
| ***Std 2x10^3^*** | 26.38 | 26.49 | 2.00E+03 | 2.00E+03 | 9063 | 7534 | 28.52 | 29.00 | 2.00E+03 | 2.00E+03 | 1795 | 1504 | 29.28 | 29.89 | 2.00E+03 | 2.00E+03 | 4972 | 3037 |
| ***Std 2x10^4^*** | 23.46 | 23.46 | 2.00E+04 | 2.00E+04 | 11364 | 9274 | 25.50 | 25.41 | 2.00E+04 | 2.00E+04 | 3145 | 2683 | 26.06 | 26.44 | 2.00E+04 | 2.00E+04 | 8219 | 4945 |
| ***Std 2x10^5^*** | 20.27 | 20.24 | 2.00E+05 | 2.00E+05 | 12809 | 9514 | 22.17 | 22.28 | 2.00E+05 | 2.00E+05 | 4142 | 3165 | 22.64 | 23.16 | 2.00E+05 | 2.00E+05 | 10561 | 5610 |
| ***Std 2x10^6^*** | 17.11 | 17.13 | 2.00E+06 | 2.00E+06 | 13043 | 10420 | 18.99 | 19.02 | 2.00E+06 | 2.00E+06 | 4499 | 3688 | 19.44 | 20.18 | 2.00E+06 | 2.00E+06 | 11520 | 6293 |
