# Supplementary material for: Development and Validation of a Harmonized TaqMan-Based Triplex Real-Time RT-PCR Protocol for the Quantitative Detection of Normalized Gene Expression Profiles of Seven Porcine Cytokines
Source: PLoS One. 2014 Sep 30;9(9):e108910. doi: 10.1371/journal.pone.0108910 (PMC4182501; doi:10.1371/journal.pone.0108910)
Supplement: Table S4 — Assessment of reproducibility by the use of a synthetic standard RNA dilution series as triplicates. (DOC) [file pone.0108910.s006.doc]

**Table S4: Assessment of reproducibility by the use of a synthetic standard RNA dilution series as triplicates.** Mean Cq-values of the 10-fold standard RNA dilution series ranging from 2x10^1^ to 2x10^6^ copies/µl are given for each channel (FAM, HEX, TR) for all cytokine triplex real-time RT-qPCR assays (IL-2, IL-4, IL-6, IL-8, IL-1β, TNF-α , IFN-α, β-Actin, GAPDH) along with corresponding Cq-deviations between the triplicates.

Std=Standard; „/“=no Cq-value detectable

| **IL-2 triplex assay** | | | | | | | **IL-1β triplex assay** | | | | | | |
| --- | --- | --- | --- | --- | --- | --- | --- | --- | --- | --- | --- | --- | --- |
| ***Standard dilution series*** | **IL-2 *FAM*** | | **β-Actin *HEX*** | | **GAPDH *TR*** | | ***Standard dilution series*** | **IL-1β *FAM*** | | **β-Actin *HEX*** | | **GAPDH *TR*** | |
|  | Cq mean | *Cq std. deviation* | Cq mean | *Cq std. deviation* | Cq mean | *Cq std.*  *deviation* |  | Cq mean | *Cq std. deviation* | Cq mean | *Cq std. deviation* | Cq mean | *Cq std. deviation* |
|  |  |  |  |  |  |  |  |  |  |  |  |  |  |
| *Std 2x10^1^* | 34.20 | *0.09* | 37.00 | *0.53* | 35.99 | *0.94* | *Std 2x10^1^* | 34.62 | *0.12* | 36.58 | *0.26* | 34.53 | *0.26* |
| *Std 2x10^2^* | 31.29 | *0.05* | 34.67 | *0.20* | 33.55 | *0.31* | *Std 2x10^2^* | 32.28 | *0.12* | 36.24 | *0.55* | 32.34 | *0.16* |
| *Std 2x10^3^* | 28.01 | *0.07* | 30.94 | *0.16* | 30.03 | *0.11* | *Std 2x10^3^* | 29.22 | *0.06* | 32.01 | *0.12* | 29.04 | *0.05* |
| *Std 2x10^4^* | 24.69 | *0.06* | 27.59 | *0.24* | 26.78 | *0.12* | *Std 2x10^4^* | 26.08 | *0.04* | 28.46 | *0.06* | 25.80 | *0.06* |
| *Std 2x10^5^* | 21.45 | *0.12* | 24.31 | *0.40* | 23.64 | *0.39* | *Std 2x10^5^* | 22.76 | *0.08* | 24.87 | *0.17* | 22.43 | *0.06* |
| *Std 2x10^6^* | 18.14 | *0.01* | 20.65 | *0.14* | 20.15 | *0.03* | *Std 2x10^6^* | 19.41 | *0.05* | 21.48 | *0.08* | 19.17 | *0.06* |
| **IL-4 triplex assay** | | | | | | | **TNF-α triplex assay** | | | | | | |
|  | **IL-2 *FAM*** | | **β-Actin *HEX*** | | **GAPDH *TR*** | |  | **IL-1β *FAM*** | | **β-Actin *HEX*** | | **GAPDH *TR*** | |
|  | Cq mean | *Cq std. deviation* | Cq mean | *Cq std. deviation* | Cq mean | *Cq std.*  *deviation* |  | Cq mean | *Cq std. deviation* | Cq mean | *Cq std. deviation* | Cq mean | *Cq std. deviation* |
|  |  |  |  |  |  |  |  |  |  |  |  |  |  |
| *Std 2x10^1^* | 36.36 | *0.50* | 35.71 | *0.50* | 35.30 | *1.41* | *Std 2x10^1^* | 34.28 | *1.23* | 35.96 | *0.70* | 37.14 | *0.05* |
| *Std 2x10^2^* | 32.18 | *0.41* | 34.96 | *1.66* | 31.88 | *0.55* | *Std 2x10^2^* | 29.47 | *0.08* | 33.65 | *0.26* | 32.25 | *0.42* |
| *Std 2x10^3^* | 28.84 | *0.08* | 30.45 | *0.21* | 28.06 | *0.05* | *Std 2x10^3^* | 26.24 | *0.03* | 30.78 | *0.06* | 29.17 | *0.09* |
| *Std 2x10^4^* | 25.65 | *0.16* | 27.04 | *0.32* | 24.92 | *0.17* | *Std 2x10^4^* | 23.04 | *0.07* | 27.44 | *0.11* | 25.78 | *0.25* |
| *Std 2x10^5^* | 22.44 | *0.04* | 23.77 | *0.15* | 21.61 | *0.06* | *Std 2x10^5^* | 19.68 | *0.02* | 24.14 | *0.01* | 22.49 | *0.04* |
| *Std 2x10^6^* | 19.24 | *0.15* | 20.35 | *0.24* | 18.43 | *0.10* | *Std 2x10^6^* | 16.44 | *0.05* | 20.88 | *0.07* | 19.27 | *0.11* |
| **IL-6 triplex assay** | | | | | | | **IFN-α triplex assay** | | | | | | |
|  | **IL-2 *FAM*** | | **β-Actin *HEX*** | | **GAPDH *TR*** | |  | **IL-1β *FAM*** | | **β-Actin *HEX*** | | **GAPDH *TR*** | |
|  | Cq mean | *Cq std. deviation* | Cq mean | *Cq std. deviation* | Cq mean | *Cq std.*  *deviation* |  | Cq mean | *Cq std. deviation* | Cq mean | *Cq std. deviation* | Cq mean | *Cq std. deviation* |
|  |  |  |  |  |  |  |  |  |  |  |  |  |  |
| *Std 2x10^1^* | 34.12 | *0.80* | / | / | / | / | *Std 2x10^1^* | 34.55 | *0.77* | 36.57 | *1.02* | 35.86 | *1.00* |
| *Std 2x10^2^* | 30.50 | *0.10* | 32.62 | *0.29* | 34.21 | *0.49* | *Std 2x10^2^* | 31.69 | *0.13* | 35.14 | *0.19* | 32.73 | *0.35* |
| *Std 2x10^3^* | 28.02 | *0.01* | 29.05 | *0.19* | 29.94 | *0.16* | *Std 2x10^3^* | 28.30 | *0.17* | 30.65 | *0.44* | 29.35 | *0.19* |
| *Std 2x10^4^* | 24.95 | *0.05* | 25.72 | *0.17* | 26.53 | *0.08* | *Std 2x10^4^* | 25.23 | *0.09* | 27.21 | *0.06* | 26.07 | *0.07* |
| *Std 2x10^5^* | 21.59 | *0.11* | 22.49 | *0.13* | 23.29 | *0.13* | *Std 2x10^5^* | 22.02 | *0.05* | 23.95 | *0.20* | 22.85 | *0.01* |
| *Std 2x10^6^* | 18.33 | *0.02* | 19.21 | *0.07* | 20.13 | *0.04* | *Std 2x10^6^* | 18.56 | *0.03* | 20.33 | *0.17* | 19.33 | *0.08* |
| **IL-8 triplex assay** | | | | | | |  | | | | | | |
| ***Standard dilution series*** | **IL-2 *FAM*** | | **β-Actin *HEX*** | | **GAPDH *TR*** | |  | | | | | | |
|  | Cq mean | *Cq std. deviation* | Cq mean | *Cq std. deviation* | Cq mean | *Cq std.*  *deviation* |  | | | | | | |
|  |  |  |  |  |  |  |  |  |  |  |  |  |  |
| *Std 2x10^1^* | 34.31 | *0.76* | / | / | / | / |  | | | | | | |
| *Std 2x10^2^* | 29.19 | *0.15* | 33.10 | *0.41* | / | / |  | | | | | | |
| *Std 2x10^3^* | 26.06 | *0.10* | 29.51 | *0.13* | 30.89 | *0.31* |  | | | | | | |
| *Std 2x10^4^* | 22.79 | *0.14* | 25.90 | *0.37* | 27.55 | *0.14* |  | | | | | | |
| *Std 2x10^5^* | 19.50 | *0.10* | 22.74 | *0.03* | 24.31 | *0.06* |  | | | | | | |
| *Std 2x10^6^* | 16.40 | *0.02* | 19.62 | *0.09* | 21.15 | *0.02* |  | | | | | | |
