# Supplementary material for: Development and Validation of a Harmonized TaqMan-Based Triplex Real-Time RT-PCR Protocol for the Quantitative Detection of Normalized Gene Expression Profiles of Seven Porcine Cytokines
Source: PLoS One. 2014 Sep 30;9(9):e108910. doi: 10.1371/journal.pone.0108910 (PMC4182501; doi:10.1371/journal.pone.0108910)
Supplement: Table S5 — Testing of experimental samples. (PDF) [file pone.0108910.s007.pdf]

Table S5: Testing of experimental samples

All seven triplex RT-qPCRs were performed with different sample matrices (leukocytes, EDTA-blood) in order to prove suitability for field samples. The following sample amounts were included in all assays:
45 wb and 85 dp infected with CSFV strain "Roesrath" along with corresponding negative controls (neg.ctr.): 24 wb, 49 dp;
24 samples from CSFV strain "Koslov" infected pigs; 17 samples from ASFV strain "Armenia" infected pigs with additional neg. ctr. (3 wb; 3 dp);
83 samples from pigs vaccinated against CSFV with "C-strain" vaccine (31 wb; 52 dp);
69 samples from pigs vaccinated against CSFV with the marker vaccine candidate "CP7_E2alf" (24 wb; 45 dp);
Relevant data from PCR assays are listed below:
Cq-values for target cytokines (IL-6, IL-8, TNF-α, IFN-α, IL-1β, IL-2, IL-4; FAM labled), for reference gene 1 (β-Actin; Ref 1) using HEX, for reference gene 2 (GAPDH; Ref 2) Texas Red (TR) labeled;
along with corresponding gene expression values of each target cytokine as ΔΔCq. Animal identities (Animal ID) are given as ear tag numbers (ET).

wb = wild boar; dp = domestic pig; DPI = days post inoculation; Vacc. = Vaccine

Animal ID	Breed	Age	Inoculum	DPI	IL-6	Ref 1	Ref 2	$\Delta\Delta C_q$	IL-8	Ref 1	Ref 2	ΔΔCq	TNF-α	Ref 1	Ref 2	ΔΔCq	IFN-α	Ref 1	Ref 2	ΔΔCq	IL-1β	Ref 1	Ref 2	ΔΔCq	IL-2	Ref 1	Ref 2	ΔΔCq	IL-4	Ref 1	Ref 2	ΔΔCq
ET 08	wb	weaner	CSFV "*Roesrath* "	0	36.95	28.20	29.13	0.0009	37.75	30.21	30.51	0.0003	33.30	29.84	30.46	0.0472	31.37	30.64	30.21	0.3347	32.34	30.66	31.21	0.1031	38.94	30.47	30.33	0.0017	N/A	28.41	30.92	N/A
				3	26.94	17.81	20.08	0.0061	24.49	22.38	21.47	0.0206	26.61	23.14	21.81	0.0285	26.91	23.44	21.73	0.0358	26.65	23.64	23.12	0.0362	N/A	23.54	21.81	N/A	N/A	23.02	22.51	N/A
				7	27.11	21.08	21.47	0.0108	20.10	21.91	21.17	1.7518	22.79	22.73	21.59	0.4470	23.34	21.43	22.78	0.4208	25.89	21.89	22.18	0.0122	31.57	21.46	21.76	0.0001	35.03	22.77	20.48	0.0000
				10	30.55	22.11	23.37	0.0026	25.29	22.59	22.76	0.1025	24.38	24.40	23.80	0.5529	23.03	22.33	24.20	1.1592	31.34	23.25	23.89	0.0006	32.31	22.95	23.59	0.0003	34.73	24.02	22.18	0.0001
				15	28.77	21.46	20.53	0.0004	20.02	21.58	20.80	0.3488	25.31	22.09	21.21	0.0410	28.49	22.70	21.08	0.0075	24.10	22.44	22.13	0.1084	N/A	22.77	21.17	N/A	N/A	21.85	22.08	N/A
ET 09	wb	weaner	CSFV "*Roesrath* "	0	40.42	29.41	30.18	0.0002	N/A	33.28	34.51	N/A	38.49	31.74	32.41	0.0044	32.71	33.41	31.74	0.5704	33.28	32.80	32.95	0.1946	38.75	32.48	32.20	0.0076	N/A	31.54	33.93	N/A
				2	34.39	27.94	28.73	0.0042	30.23	27.97	27.66	0.1306	29.69	30.05	28.24	0.4236	N/A	29.89	29.26	N/A	31.68	28.20	29.00	0.0089	N/A	28.46	27.20	N/A	N/A	28.94	28.68	N/A
				3	32.05	24.16	25.34	0.0036	23.14	23.98	24.71	1.4027	29.20	25.71	25.31	0.0512	36.07	24.22	26.54	0.0006	29.61	25.10	26.10	0.0085	30.80	25.06	25.47	0.0031	34.61	25.75	24.05	0.0003
				7	35.75	23.98	24.78	0.0002	25.16	24.42	24.37	0.3579	26.86	26.13	25.10	0.2759	27.18	24.36	26.20	0.2653	31.57	25.10	25.54	0.0016	N/A	24.55	25.26	N/A	31.21	25.72	23.75	0.0029
				10	31.30	22.70	22.95	0.0017	25.28	22.64	22.57	0.0986	24.67	24.08	23.35	0.3469	23.27	22.36	23.91	0.8997	32.44	23.24	23.54	0.0002	N/A	22.53	23.41	N/A	N/A	23.74	21.87	N/A
				14	35.45	25.64	25.89	0.0007	27.04	25.92	25.24	0.2163	29.80	27.62	26.26	0.0882	N/A	26.40	27.27	N/A	33.21	26.97	26.92	0.0014	22.82	25.93	26.02	0.3926	N/A	27.05	24.30	N/A
				21	41.37	27.18	27.00	0.0000	27.24	26.51	26.13	0.3888	30.62	28.55	26.32	0.0660	N/A	28.12	27.56	N/A	37.19	27.19	27.55	0.0001	N/A	27.00	25.12	N/A	N/A	27.96	27.30	N/A
				23	31.51	24.13	23.88	0.0017	26.27	23.59	22.96	0.0916	26.82	25.65	23.28	0.1251	N/A	24.32	24.05	N/A	29.91	24.08	24.26	0.0017	38.38	23.63	22.45	0.0000	N/A	24.78	24.17	N/A
ET 10	wb	weaner	CSFV "*Roesrath* "	0	42.55	29.18	29.16	0.0000	37.99	32.31	32.57	0.0009	34.30	30.20	30.78	0.0294	31.39	31.79	30.47	0.5342	32.30	31.51	31.88	0.1771	N/A	32.07	30.81	N/A	N/A	31.51	31.87	N/A
				1	34.06	26.25	27.82	0.0022	30.81	25.67	27.10	0.0317	29.87	27.80	27.40	0.1278	29.81	26.26	28.64	0.1763	31.01	26.10	28.05	0.0053	N/A	25.78	26.51	N/A	30.13	26.83	28.03	0.0100
				2	38.71	30.51	30.75	0.0009	33.64	30.04	29.83	0.0511	33.60	33.35	30.73	0.1955	N/A	33.03	32.28	N/A	33.27	30.20	30.96	0.0102	N/A	30.56	29.10	N/A	N/A	31.27	31.05	N/A
				3	28.00	21.48	20.91	0.0055	20.81	21.52	20.46	0.7373	24.57	23.26	21.17	0.1357	23.59	22.05	22.20	0.3580	25.83	22.24	21.66	0.0120	30.78	21.76	21.26	0.0002	35.50	23.16	19.86	0.0000
				7	32.76	23.45	24.32	0.0012	25.25	23.89	23.96	0.2444	26.02	25.34	24.63	0.3217	24.91	23.63	25.35	0.7408	32.84	24.47	25.00	0.0004	N/A	24.52	24.82	N/A	34.07	25.02	23.25	0.0002
				10	39.18	29.66	29.78	0.0007	30.66	30.73	29.23	0.3396	33.76	31.88	30.12	0.0884	N/A	32.75	33.11	N/A	41.70	31.17	30.81	0.0000	N/A	30.07	30.05	N/A	40.16	31.34	28.41	0.0001
				14	33.90	25.81	26.13	0.0023	28.33	26.47	26.01	0.1378	30.03	27.66	26.48	0.0822	N/A	26.82	27.83	N/A	34.23	27.08	27.34	0.0008	32.91	26.76	26.39	0.0015	31.82	27.22	25.05	0.0048
				17	30.32	22.82	23.49	0.0023	24.14	22.59	22.29	0.2323	25.67	24.69	23.12	0.1928	23.79	22.83	23.15	0.5269	29.06	23.10	23.42	0.0017	N/A	22.65	22.38	N/A	N/A	23.57	23.82	N/A
ET 13	wb	weaner	CSFV "*Roesrath* "	0	35.91	27.04	27.49	0.0007	31.12	28.06	28.74	0.0106	31.18	27.85	28.45	0.0542	30.24	28.73	28.30	0.2012	31.05	28.72	29.56	0.0775	42.85	28.94	28.46	0.0000	N/A	27.84	29.23	N/A
				1	37.23	31.16	31.92	0.0047	39.88	31.36	30.51	0.0012	38.82	34.94	31.13	0.0095	N/A	36.00	35.69	N/A	36.34	31.44	32.39	0.0024	N/A	31.29	29.82	N/A	N/A	32.74	32.17	N/A
				2	33.98	27.95	28.45	0.0050	30.23	28.11	27.57	0.1333	29.47	30.34	28.21	0.5409	N/A	29.77	29.38	N/A	31.13	28.24	28.91	0.0134	N/A	28.27	27.08	N/A	N/A	28.65	28.52	N/A
				3	N/A	27.53	27.57	N/A	26.80	28.24	28.44	0.2477	39.61	28.11	28.36	0.0002	34.35	29.07	28.20	0.0126	33.00	29.10	29.80	0.0234	40.61	29.22	28.72	0.0002	N/A	28.52	29.62	N/A
				7	25.56	19.06	20.21	0.0105	18.57	19.02	19.70	1.1648	20.84	21.00	20.48	0.6624	19.66	19.48	21.11	1.5411	24.64	20.08	20.60	0.0097	31.75	19.32	20.55	0.0000	34.93	20.73	19.22	0.0000
				10	30.14	21.93	23.22	0.0031	25.48	22.45	22.61	0.0815	24.64	24.16	23.34	0.3633	22.79	22.30	24.03	1.2794	31.74	23.43	24.01	0.0005	N/A	22.99	23.52	N/A	N/A	23.95	21.96	N/A
				14	34.02	24.38	24.35	0.0007	26.22	25.11	24.02	0.1932	28.49	26.25	24.67	0.0801	44.21	24.87	25.76	0.0000	31.96	25.77	25.67	0.0016	N/A	24.73	24.64	N/A	27.86	26.02	23.36	0.0320
				21	39.40	29.45	29.45	0.0002	27.93	28.82	28.65	1.2890	29.53	31.34	29.13	1.0060	N/A	32.20	31.22	N/A	33.06	29.39	29.90	0.0063	N/A	28.96	27.43	N/A	N/A	29.97	29.44	N/A
				24	29.64	20.93	21.44	0.0010	24.02	21.01	20.38	0.0742	23.02	22.78	20.96	0.3078	22.10	20.98	21.34	0.4791	27.99	21.23	22.00	0.0013	N/A	20.36	20.00	N/A	27.03	21.43	21.53	0.0018
ET 15	wb	weaner	CSFV "*Roesrath* "	0	32.22	26.27	26.29	0.0051	27.63	26.23	26.52	0.0375	29.18	27.01	27.22	0.1105	28.52	27.36	26.97	0.2653	28.64	27.49	28.07	0.1739	37.52	27.43	27.07	0.0005	N/A	27.01	28.15	N/A
				1	39.26	30.36	30.84	0.0006	N/A	29.82	29.46	N/A	34.72	32.68	30.21	0.0580	N/A	34.13	33.98	N/A	35.17	30.15	30.97	0.0024	N/A	30.76	29.20	N/A	N/A	30.79	31.10	N/A
				2	33.28	27.02	27.95	0.0052	29.97	27.21	26.73	0.0869	29.09	28.72	27.34	0.2986	33.36	27.97	28.50	0.0257	30.97	27.06	28.08	0.0076	N/A	27.49	26.64	N/A	26.32	27.29	28.26	0.2571
				3	33.15	25.36	25.97	0.0031	24.96	25.46	25.61	0.8839	30.62	27.38	26.28	0.0464	N/A	25.99	27.54	N/A	31.47	26.42	26.74	0.0041	N/A	25.68	26.26	N/A	N/A	27.14	24.97	N/A
				10	37.66	26.76	26.83	0.0003	27.68	27.09	26.29	0.2924	29.42	28.68	27.24	0.2279	N/A	28.11	28.75	N/A	35.89	27.80	27.73	0.0003	N/A	27.39	27.17	N/A	28.93	28.39	25.67	0.0728
				7	28.42	20.93	22.62	0.0060	24.05	21.78	22.24	0.1549	23.47	23.41	23.00	0.5648	22.05	21.60	23.46	1.3782	28.55	22.62	23.20	0.0030	N/A	22.31	23.11	N/A	27.01	23.22	21.63	0.0126
				14	37.04	25.42	25.91	0.0001	27.53	25.50	26.45	0.0315	31.13	25.38	26.16	0.0111	33.16	27.02	26.17	0.0072	32.15	26.43	27.71	0.0086	N/A	26.40	26.47	N/A	N/A	25.32	27.39	N/A
				21	29.33	23.02	23.52	0.0051	26.07	22.15	22.51	0.0542	24.15	24.35	23.15	0.5098	22.64	22.44	23.38	1.1066	29.88	22.22	23.30	0.0007	N/A	22.91	21.93	N/A	N/A	23.33	23.82	N/A
				28	29.50	22.73	22.29	0.0027	26.28	21.41	21.22	0.0228	24.27	23.85	21.74	0.2402	24.58	22.79	22.36	0.2277	29.22	21.87	22.41	0.0007	N/A	22.22	20.90	N/A	N/A	23.03	22.52	N/A
ET 16	wb	weaner	CSFV "*Roesrath* "	0	37.48	25.51	26.51	0.0001	31.10	26.23	27.16	0.0036	31.10	26.57	27.26	0.0248	29.34	27.27	27.28	0.1617	30.29	27.25	28.33	0.0538	N/A	27.16	27.26	N/A	N/A	25.87	28.09	N/A
				3	34.54	27.54	27.19	0.0020	27.59	28.83	28.06	0.1449	37.17	27.85	27.78	0.0006	34.31	28.96	27.76	0.0108	32.30	29.21	29.26	0.0332	N/A	29.22	28.23	N/A	N/A	28.62	29.65	N/A

				7	33.38	23.98	24.61	0.0010	26.04	24.19	24.24	0.1722	25.73	25.68	25.05	0.5085	24.74	24.09	25.69	1.0996	35.27	25.29	25.71	0.0001	35.30	23.98	24.89	0.0001	24.22	25.37	23.50	0.3837
				10	37.39	24.33	24.97	0.0001	27.25	24.87	24.40	0.0987	27.41	26.51	25.28	0.2276	26.37	24.54	25.92	0.4490	33.65	25.39	25.50	0.0004	N/A	24.46	25.16	N/A	N/A	25.88	23.67	N/A
				14	34.79	24.04	24.04	0.0003	25.84	24.28	23.41	0.1540	27.68	25.62	24.15	0.0960	32.62	24.65	25.17	0.0047	32.39	25.55	25.35	0.0009	N/A	24.17	24.09	N/A	17.33	25.39	22.57	1.5980
				15	32.52	23.45	24.07	0.0013	27.39	23.75	23.57	0.0465	25.99	25.36	24.29	0.2960	24.34	23.48	25.11	0.9596	33.65	24.64	24.75	0.0002	37.05	23.79	24.27	0.0000	31.18	25.09	22.97	0.0018
ET 07	wb	weaner	neg.ctr."*Roesrath*"	0	40.05	30.51	30.23	0.0003	35.65	33.42	31.71	0.0053	36.00	31.24	31.72	0.0172	30.99	31.94	30.67	0.7928	32.64	32.07	32.63	0.2169	N/A	32.99	31.31	N/A	N/A	32.69	32.72	N/A
				1	N/A	32.79	32.86	N/A	N/A	31.96	31.42	N/A	38.98	37.14	32.37	0.0277	N/A	38.76	38.06	N/A	42.19	32.05	33.18	0.0000	N/A	31.33	30.44	N/A	N/A	32.41	33.48	N/A
				2	N/A	30.85	29.76	N/A	N/A	30.59	29.50	N/A	N/A	29.97	29.13	N/A	N/A	30.58	29.35	N/A	29.71	21.74	22.83	0.0005	N/A	31.24	30.20	N/A	N/A	30.68	29.76	N/A
				3	34.09	25.93	25.92	0.0019	26.46	26.03	25.78	0.4006	30.19	27.57	26.25	0.0661	35.72	26.17	27.64	0.0022	30.34	26.57	26.67	0.0099	N/A	26.31	26.23	N/A	32.07	27.35	24.81	0.0038
				7	24.41	18.33	19.08	0.0125	18.02	18.52	18.71	1.0336	21.05	20.17	19.38	0.2967	19.83	18.92	20.18	0.8183	22.92	19.29	19.64	0.0196	30.90	18.64	19.27	0.0000	35.40	20.02	18.14	0.0000
				10	35.80	24.18	24.69	0.0002	25.80	24.03	24.14	0.1855	26.41	26.10	25.28	0.3963	25.65	24.42	26.06	0.7436	33.17	25.15	25.64	0.0005	N/A	24.51	25.10	N/A	N/A	25.53	23.40	N/A
				14	32.92	23.42	24.62	0.0012	23.68	23.64	24.11	0.6992	26.15	25.61	24.89	0.3516	26.54	24.41	25.90	0.3766	29.58	24.72	25.43	0.0061	31.50	24.27	25.04	0.0012	40.55	25.14	23.50	0.0000
				28	30.93	22.15	23.29	0.0011	25.52	22.04	22.20	0.0694	23.61	24.05	22.84	0.6102	22.61	21.95	22.89	0.8074	N/A	44.64	41.44	N/A	N/A	22.39	21.47	N/A	N/A	23.17	23.48	N/A
ET 12	wb	weaner	neg.ctr."*Roesrath*"	0	38.86	29.63	28.65	0.0004	37.98	31.96	31.04	0.0005	34.16	29.74	30.30	0.0174	32.31	30.11	30.69	0.3519	32.89	31.82	31.73	0.1218	39.05	31.68	30.80	0.0029	24.22	30.72	31.84	1.2600
				1	33.50	26.49	27.87	0.0036	31.65	25.92	27.06	0.0188	31.02	27.78	27.41	0.0561	29.93	26.71	28.79	0.1992	32.69	26.63	28.28	0.0019	N/A	26.26	26.50	N/A	N/A	27.05	28.21	N/A
				2	38.91	29.10	30.57	0.0005	33.82	28.63	29.75	0.0266	32.80	30.66	29.65	0.0934	37.81	30.82	32.09	0.0109	33.23	28.59	30.62	0.0054	N/A	28.50	29.12	N/A	32.59	29.47	30.59	0.0088
				3	28.27	19.49	20.56	0.0021	19.35	19.48	20.20	0.9359	23.09	21.43	20.86	0.1830	22.64	20.20	21.96	0.3344	25.33	20.84	21.47	0.0101	33.12	20.35	21.01	0.0000	29.27	21.21	19.58	0.0006
				7	26.65	19.19	20.55	0.0057	22.08	19.53	20.17	0.1417	21.19	21.29	20.97	0.6766	19.52	19.49	21.27	1.7976	27.39	20.25	20.81	0.0014	N/A	19.76	21.03	N/A	17.91	21.02	19.50	1.8960
				10	29.95	21.58	22.68	0.0026	23.44	21.95	22.37	0.2625	23.29	23.46	23.06	0.6654	22.57	22.07	23.49	1.1456	28.11	22.67	23.26	0.0043	N/A	22.24	23.11	N/A	25.00	23.36	21.61	0.0572
				14	30.14	21.43	22.54	0.0021	21.66	21.94	22.06	0.8064	23.20	23.56	22.82	0.6761	22.73	21.90	23.24	0.8866	28.16	22.76	23.05	0.0040	N/A	22.19	22.80	N/A	33.72	23.21	21.29	0.0001
				21	27.26	19.14	20.57	0.0022	19.89	18.81	19.35	0.4550	22.36	20.85	20.02	0.1832	20.84	19.95	20.66	0.6372	24.40	18.98	20.41	0.0054	31.52	18.86	18.95	0.0000	37.12	19.97	20.78	0.0000
				28	31.60	23.17	24.16	0.0013	25.80	23.16	23.15	0.1168	24.75	25.06	23.84	0.5449	23.65	23.26	23.92	0.8747	30.12	23.06	23.90	0.0009	N/A	22.76	22.33	N/A	N/A	24.00	24.24	N/A
ET 14	wb	weaner	neg.ctr."*Roesrath*"	0	39.42	28.87	29.29	0.0002	32.28	29.45	29.40	0.0087	29.87	26.61	29.43	0.0558	30.26	27.61	29.64	0.4978	32.36	30.57	30.48	0.0762	N/A	30.61	29.81	N/A	N/A	29.59	30.81	N/A
				3	N/A	27.85	30.35	N/A	N/A	32.39	40.00	N/A	34.09	30.43	27.04	0.0311	29.58	32.22	26.80	0.1303	30.20	27.60	28.16	0.0602	N/A	27.60	26.98	N/A	33.08	26.22	27.72	0.0015
				7	25.86	18.57	19.71	0.0061	18.39	19.00	19.19	1.1068	21.10	20.40	20.00	0.3835	19.38	19.15	20.74	1.4713	24.52	19.57	20.19	0.0077	27.57	19.03	20.05	0.0007	N/A	20.22	18.79	N/A
				10	29.60	21.33	22.57	0.0030	23.16	21.46	22.18	0.2537	23.42	23.41	23.05	0.5960	22.30	21.65	23.34	1.1329	28.14	22.28	22.90	0.0033	32.40	21.96	22.93	0.0001	35.09	23.12	21.45	0.0000
				14	29.28	21.47	22.42	0.0037	23.64	21.91	21.87	0.1904	23.44	23.52	22.77	0.5528	22.45	22.09	23.34	1.1900	28.37	22.52	23.02	0.0031	N/A	22.15	22.83	N/A	27.62	23.11	21.33	0.0070
				21	26.12	19.57	21.11	0.0071	20.43	19.87	20.07	0.5777	22.54	21.65	20.54	0.2544	21.46	20.50	21.18	0.5993	26.03	20.18	21.29	0.0031	24.27	20.32	19.42	0.0152	N/A	21.00	21.28	N/A
				23	29.51	21.29	22.34	0.0017	25.49	21.16	21.52	0.0410	22.28	23.18	22.01	0.8637	21.90	21.22	22.04	0.7632	30.87	21.45	22.37	0.0002	N/A	20.89	20.75	N/A	N/A	22.16	22.56	N/A
ET 49	dp	weaner	CSFV "*Roesrath*"	0	37.80	26.21	26.56	0.0001	30.11	26.35	26.63	0.0065	29.92	27.00	27.36	0.0681	28.26	27.54	26.92	0.3297	29.28	27.39	28.02	0.1041	41.19	27.72	27.32	0.0001	N/A	26.28	28.03	N/A
				1	N/A	31.14	32.45	N/A	N/A	30.07	30.77	N/A	44.12	33.15	32.07	0.0002	N/A	35.24	37.03	N/A	39.60	30.42	32.12	0.0001	N/A	30.21	30.54	N/A	N/A	31.55	32.50	N/A
				2	36.96	31.54	32.10	0.0069	N/A	31.18	30.40	N/A	36.30	35.59	31.17	0.0720	N/A	36.02	36.56	N/A	37.61	30.60	32.92	0.0008	N/A	30.62	29.53	N/A	39.28	31.80	32.31	0.0002
				3	31.53	21.71	21.36	0.0003	21.89	22.41	22.19	0.1834	26.34	22.19	22.25	0.0294	29.03	23.11	22.03	0.0082	28.06	23.47	23.28	0.0131	38.41	23.11	22.19	0.0000	34.10	22.32	23.09	0.0030
				7	35.10	23.95	25.27	0.0004	26.41	24.29	24.79	0.1657	26.27	25.83	25.65	0.4512	26.17	24.32	26.40	0.5618	34.78	25.03	25.95	0.0002	27.13	24.30	25.57	0.0379	21.94	25.36	24.10	1.8500
				10	30.51	22.26	23.51	0.0030	25.57	22.62	23.19	0.0988	25.09	24.45	24.16	0.3883	23.42	23.05	25.04	1.5266	32.53	23.38	24.11	0.0003	N/A	22.46	23.79	N/A	23.72	23.88	22.28	0.2193
				14	N/A	31.01	31.51	N/A	27.18	25.53	26.05	0.2265	30.58	28.36	27.13	0.0884	N/A	26.99	28.62	N/A	32.53	27.98	28.15	0.0051	N/A	26.49	26.72	N/A	25.09	27.63	25.51	0.8732
				21	33.37	27.13	26.78	0.0033	27.94	26.98	25.55	0.2263	30.00	28.94	26.66	0.1323	N/A	27.99	27.09	N/A	33.46	27.32	26.83	0.0008	N/A	26.80	25.04	N/A	N/A	27.53	26.69	N/A
				24	30.30	22.65	23.24	0.0020	25.57	22.22	22.12	0.0693	24.27	24.45	22.82	0.4336	N/A	26.59	23.38	N/A	35.64	26.16	26.91	0.0001	N/A	22.55	21.76	N/A	N/A	23.58	23.44	N/A
ET 50	dp	weaner	CSFV "*Roesrath*"	0	35.53	26.13	26.81	0.0005	32.75	26.54	27.27	0.0012	30.26	27.20	27.93	0.0700	28.84	28.02	27.63	0.3326	30.21	28.05	28.77	0.0860	N/A	28.01	27.69	N/A	33.56	26.83	28.57	0.0024
				1	38.11	27.21	28.91	0.0002	35.73	26.59	27.89	0.0017	33.07	29.11	28.48	0.0301	32.12	28.41	29.83	0.1121	32.70	27.18	29.17	0.0031	N/A	27.45	27.53	N/A	N/A	28.00	29.04	N/A
				2	N/A	30.46	30.58	N/A	35.99	29.85	30.03	0.0095	31.68	31.90	30.07	0.3689	N/A	32.62	32.25	N/A	33.23	29.92	30.70	0.0088	N/A	29.48	29.02	N/A	N/A	30.56	30.83	N/A
				3	N/A	27.53	28.74	N/A	26.48	28.62	29.44	0.4967	37.13	29.09	29.54	0.0018	34.42	29.57	29.27	0.0204	33.50	29.25	30.45	0.0216	N/A	29.49	29.57	N/A	N/A	27.92	30.34	N/A
				7	39.05	26.09	27.28	0.0001	26.83	26.64	27.22	0.6209	29.40	28.30	27.76	0.2430	38.40	27.12	29.35	0.0009	39.27	27.55	28.40	0.0000	N/A	26.67	27.96	N/A	33.61	28.08	26.52	0.0029
				10	30.07	21.75	23.24	0.0031	24.84	22.32	22.58	0.1206	23.63	23.53	23.30	0.5848	22.60	22.20	23.80	1.3060	29.78	22.74	23.60	0.0014	N/A	22.37	23.35	N/A	29.44	23.37	21.92	0.0025
				14	36.68	26.48	28.02	0.0008	24.63	24.94	25.56	0.9218	27.50	26.78	26.17	0.3168	N/A	25.85	27.72	N/A	30.26	26.19	27.10	0.0107	33.77	25.56	26.23	0.0005	24.64	26.67	24.60	0.6421
				18	30.62	23.37	24.45	0.0031	26.58	23.19	23.35	0.0728	24.65	25.26	24.03	0.6674	24.44	23.33	24.24	0.5812	30.98	23.43	24.51	0.0006	28.23	23.26	22.79	0.0073	N/A	24.32	24.66	N/A
ET 51	dp	weaner	CSFV "*Roesrath*"	0	N/A	29.75	30.15	N/A	N/A	30.38	30.65	N/A	36.45	30.04	30.47	0.0055	31.16	31.07	30.75	0.5398	33.80	31.25	31.79	0.0537	44.64	31.09	30.41	0.0000	N/A	30.06	31.74	N/A
				2	34.75	27.40	28.43	0.0024	35.23	30.17	31.13	0.0272	29.19	29.04	28.08	0.4031	28.64	28.05	29.28	0.9123	31.11	27.00	28.70	0.0083	N/A	27.49	27.24	N/A	N/A	28.15	28.62	N/A
				3	28.47	18.92	20.48	0.0014	20.14	19.50	20.26	0.5566	22.23	21.34	21.10	0.3485	22.37	20.22	22.10	0.4268	25.48	20.75	21.88	0.0101	22.61	20.07	21.13	0.0577	35.38	21.02	19.66	0.0000
				10	32.28	23.22	24.11	0.0015	26.05	23.06	23.61	0.0947	24.91	25.00	24.59	0.6104	23.83	22.95	24.85	1.0416	32.53	24.10	24.58	0.0004	N/A	23.47	24.36	N/A	N/A	24.62	22.98	N/A
				14	30.34	21.43	22.75	0.0019	23.63	21.71	22.15	0.1969	23.15	23.32	22.95	0.6761	22.35	21.59	23.38	1.0843	29.50	22.70	23.42	0.0016	N/A	21.39	22.97	N/A	33.84	23.13	21.47	0.0001
				17	30.86	21.26	23.20	0.0009	25.75	21.63	21.83	0.0445	23.24	23.47	22.55	0.5836	21.99	21.61	22.36	0.9133	29.86	21.46	22.44	0.0004	N/A	21.78	21.38	N/A	N/A	22.78	23.38	N/A
ET 52	dp	weaner	CSFV "*Roesrath*"	0	N/A	26.49	28.13	N/A	42.63	26.46	27.93	0.0000	33.21	27.35	28.60	0.0115	29.10	28.19	28.32	0.3700	32.28	27.83	29.26	0.0215	N/A	28.38	28.82	N/A	N/A	26.28	29.40	N/A
				1	39.19	29.49	30.25	0.0004	34.14	29.48	29.18	0.0234	33.50	31.14	29.68	0.0680	N/A	30.92	31.10	N/A	34.15	29.00	29.90	0.0024	N/A	29.20	28.58	N/A	N/A	30.00	30.31	N/A
				2	31.83	24.85	26.18	0.0039	31.64	27.20	27.72	0.0376	26.89	26.24	25.46	0.3160	26.13	25.11	26.04	0.6168	29.94	24.62	26.26	0.0039	N/A	24.53	24.63	N/A	19.04	25.21	26.30	1.4201
				3	N/A	28.28	29.34	N/A	28.24	29.33	30.68	0.2573	N/A	28.60	30.03	N/A	N/A	29.75	29.41	N/A	36.90	29.78	31.05	0.0028	N/A	29.82	30.10	N/A	N/A	28.61	31.18	N/A
				7	28.94	20.05	21.18	0.0019	21.69	20.35	20.58	0.2810	21.65	22.15	21.43	0.7659	21.34	20.30	21.97	0.8604	27.82	21.09	21.56	0.0017	13.74	20.68	21.52	0.0670	22.28	21.85	20.15	0.1508
				10	31.48	23.19	23.90	0.0024	25.16	22.90	23.40	0.1551	24.66	24.77	24.21	0.5912	23.56	23.05	24.67	1.2146	31.86	23.71	24.34	0.0005	24.14	23.34	24.16	0.1565	N/A	24.34	22.76	N/A
				14	35.53	25.03	26.26	0.0006	25.21	25.32	26.03	0.8195	27.81	27.06	26.53	0.3163	40.28	25.79	27.76	0.0001	32.17	26.21	27.20	0.0026	24.72	25.76	26.48	0.5061	N/A	26.55	25.11	N/A

ET 53	dp	weaner	CSFV "*Roesrath*"	0	39.17	27.19	28.40	0.0001	35.30	27.60	28.46	0.0004	33.72	27.68	29.02	0.0103	29.74	28.83	28.91	0.3606	31.57	29.08	30.14	0.0735	N/A	29.10	29.35	N/A	N/A	28.50	30.31	N/A
				1	N/A	28.79	30.84	N/A	38.08	29.04	29.51	0.0014	36.09	30.45	30.30	0.0106	38.99	29.94	32.59	0.0042	33.62	29.02	30.72	0.0048	N/A	28.69	29.06	N/A	N/A	29.46	30.76	N/A
				2	37.60	28.85	29.53	0.0007	31.48	28.51	28.86	0.0994	30.17	31.00	29.18	0.5768	41.76	30.28	30.58	0.0004	32.14	28.94	29.49	0.0095	N/A	28.36	28.06	N/A	N/A	29.50	30.04	N/A
				21	32.20	25.23	25.31	0.0025	27.39	25.04	24.20	0.1053	27.32	27.01	24.82	0.2400	31.61	25.52	25.40	0.0128	32.06	25.31	25.64	0.0008	N/A	24.69	23.57	N/A	N/A	25.70	25.46	N/A
ET 54	dp	weaner	CSFV "*Roesrath*"	0	39.67	26.13	27.31	0.0000	31.43	26.41	27.58	0.0034	30.35	26.57	27.75	0.0498	29.74	27.91	27.81	0.1809	30.30	27.59	28.76	0.0693	39.45	27.57	27.72	0.0002	40.07	26.07	28.52	0.0000
				2	37.15	28.14	29.13	0.0007	31.51	27.71	28.07	0.0554	29.03	29.52	28.50	0.6163	37.73	29.56	29.85	0.0034	31.87	28.18	29.34	0.0086	N/A	27.91	27.46	N/A	N/A	29.87	29.36	N/A
				3	N/A	27.12	28.11	N/A	26.98	27.95	27.93	1.1005	30.89	29.59	28.74	0.1874	N/A	28.38	30.64	N/A	34.72	28.75	29.21	0.0018	35.06	27.95	28.74	0.0010	36.23	29.06	27.19	0.0007
				7	29.69	21.58	23.06	0.0036	25.03	22.01	22.46	0.0910	23.46	23.62	23.30	0.6802	22.18	21.78	23.52	1.3625	31.22	22.47	23.30	0.0004	35.00	22.58	23.26	0.0000	N/A	23.43	21.91	N/A
				10	29.44	21.35	22.45	0.0032	25.14	21.71	22.14	0.0689	23.47	23.54	23.07	0.6049	21.99	21.25	23.18	1.1569	31.81	22.27	22.79	0.0002	37.24	21.75	22.82	0.0000	N/A	22.97	21.33	N/A
				20	26.49	21.32	22.30	0.0145	22.08	21.19	21.28	0.4312	25.49	23.40	21.82	0.0890	25.37	22.42	22.61	0.1265	28.16	21.78	22.56	0.0016	N/A	21.87	20.68	N/A	N/A	22.50	22.62	N/A
ET 55	dp	weaner	CSFV "*Roesrath*"	0	33.30	24.91	26.48	0.0029	28.97	25.78	26.36	0.0786	27.23	27.05	26.83	0.5248	23.96	25.34	28.18	6.8720	29.61	26.23	27.57	0.0209	N/A	25.64	26.74	N/A	30.00	26.75	25.42	0.0175
				1	31.46	24.05	25.47	0.0031	29.26	23.80	24.51	0.0201	27.58	25.65	25.07	0.1376	23.95	23.76	25.18	1.3112	28.60	23.31	25.15	0.0048	29.24	23.43	24.02	0.0057	37.27	24.35	25.51	0.0000
				2	31.82	25.92	27.02	0.0074	29.76	25.50	25.84	0.0406	27.50	27.32	26.52	0.4290	28.74	26.36	27.28	0.2379	29.84	25.10	27.18	0.0068	N/A	25.78	25.59	N/A	N/A	28.35	28.66	N/A
				3	35.81	24.83	25.74	0.0004	26.48	25.01	25.28	0.2381	26.93	26.78	26.04	0.4504	26.12	24.99	26.79	0.8416	32.24	25.73	26.31	0.0016	34.91	25.25	25.91	0.0002	25.26	26.14	24.60	0.3417
				7	28.44	21.33	22.25	0.0060	24.05	21.71	21.92	0.1358	22.97	23.16	22.60	0.6402	21.41	21.11	22.99	1.5427	29.63	22.08	22.63	0.0009	24.37	21.26	22.54	0.0377	24.44	22.71	21.21	0.0602
				10	35.49	24.62	25.47	0.0004	27.85	25.04	25.13	0.0880	26.62	26.54	25.99	0.5067	25.87	24.75	26.52	0.8399	36.14	25.61	26.10	0.0001	N/A	24.85	25.92	N/A	27.79	26.09	24.47	0.0504
				11	38.60	26.85	24.96	0.0000	28.00	27.37	25.58	0.0291	30.86	27.03	25.40	0.0180	30.13	28.30	25.34	0.0686	31.34	28.11	26.83	0.0196	N/A	28.15	25.58	N/A	N/A	26.58	26.41	N/A
ET 56	dp	weaner	CSFV "*Roesrath*"	0	38.27	28.43	29.13	0.0004	39.68	29.69	29.57	0.0000	32.13	28.93	29.40	0.0551	29.92	29.89	29.31	0.5250	31.20	30.05	30.27	0.1381	N/A	30.16	29.30	N/A	N/A	29.41	30.40	N/A
				1	N/A	29.47	31.90	N/A	N/A	28.93	30.93	N/A	42.69	32.12	31.54	0.0003	N/A	32.22	34.38	N/A	37.49	29.69	32.09	0.0005	N/A	30.13	30.60	N/A	N/A	30.59	31.33	N/A
				2	37.19	31.34	31.99	0.0053	38.57	30.91	30.99	0.0031	32.33	34.16	30.93	0.6777	N/A	38.47	34.23	N/A	33.87	31.08	31.75	0.0114	N/A	31.04	29.79	N/A	N/A	30.95	31.26	N/A
				3	31.66	24.56	24.57	0.0042	24.60	24.87	24.18	0.5764	26.06	26.21	24.86	0.4547	N/A	25.34	26.62	N/A	28.92	25.49	25.63	0.0139	N/A	24.84	24.68	N/A	32.06	25.97	23.51	0.0016
				7	28.36	20.77	22.05	0.0049	25.08	21.02	21.55	0.0463	23.14	22.94	22.41	0.4975	21.13	21.07	22.67	1.6576	30.62	21.78	22.28	0.0003	34.51	21.23	22.35	0.0000	N/A	22.55	21.02	N/A
				10	28.99	21.44	22.49	0.0046	26.02	22.06	22.02	0.0401	23.32	23.30	22.73	0.5504	21.92	21.34	23.12	1.2319	30.39	22.37	22.87	0.0006	N/A	21.63	22.76	N/A	25.05	23.09	21.27	0.0446
				14	34.87	26.37	26.51	0.0016	27.33	26.67	26.19	0.3124	28.78	28.16	26.85	0.2611	N/A	27.68	28.29	N/A	31.55	27.87	27.64	0.0087	N/A	26.92	26.76	N/A	30.72	28.17	25.33	0.0163
				21	32.98	22.11	23.75	0.0003	25.15	21.35	21.96	0.0647	23.64	23.65	22.74	0.5021	21.32	21.74	22.79	1.7691	29.98	21.54	23.01	0.0004	N/A	21.62	21.51	N/A	N/A	22.82	23.34	N/A
				28	33.30	22.52	24.06	0.0003	26.36	22.68	23.05	0.0642	24.25	24.48	23.52	0.5688	23.35	22.45	23.68	0.7528	31.18	22.40	23.82	0.0003	16.94	22.58	22.44	0.7508	N/A	23.63	24.12	N/A
ET 57	dp	weaner	CSFV "*Roesrath*"	0	38.07	29.13	29.26	0.0006	N/A	30.12	29.86	N/A	32.88	29.43	30.07	0.0485	29.77	30.48	29.59	0.7798	31.75	30.64	31.13	0.1525	N/A	30.85	30.17	N/A	N/A	29.06	30.57	N/A
				1	N/A	32.52	33.36	N/A	N/A	33.47	31.82	N/A	N/A	34.67	33.90	N/A	N/A	38.14	36.68	N/A	42.06	32.83	33.45	0.0001	N/A	18.26	30.95	N/A	26.29	33.97	33.73	0.9218
				2	39.39	28.82	30.14	0.0003	35.02	29.13	28.83	0.0097	30.95	30.55	29.16	0.2820	41.38	30.70	30.89	0.0006	32.95	28.59	29.92	0.0053	N/A	28.84	28.15	N/A	N/A	29.49	30.51	N/A
				3	29.50	17.46	19.64	0.0002	26.79	29.32	28.93	0.4115	35.58	27.87	28.43	0.0024	35.00	29.34	28.60	0.0101	32.25	29.10	29.93	0.0419	40.21	29.00	28.69	0.0002	N/A	28.35	29.50	N/A
				7	30.87	23.16	23.96	0.0037	24.79	23.30	23.61	0.2449	24.85	24.96	24.28	0.5664	23.60	23.03	24.89	1.2660	31.63	24.13	24.58	0.0008	N/A	23.52	24.27	N/A	16.51	24.60	22.66	1.0240
				10	28.91	21.24	22.36	0.0044	24.27	21.81	22.11	0.1289	22.67	23.18	22.69	0.8187	21.72	21.27	23.07	1.3454	29.06	22.48	22.96	0.0018	N/A	21.84	22.69	N/A	N/A	22.94	21.31	N/A
				14	33.92	25.81	26.03	0.0022	25.18	25.67	25.42	0.7649	27.46	27.13	26.15	0.3642	N/A	26.67	27.82	N/A	30.77	26.64	27.17	0.0087	N/A	25.98	26.06	N/A	N/A	27.10	24.78	N/A
				18	30.50	22.91	24.02	0.0025	24.99	22.59	22.86	0.1540	24.24	24.67	23.45	0.5950	23.67	22.79	23.66	0.6740	30.82	23.08	23.94	0.0005	N/A	22.67	22.21	N/A	N/A	23.91	24.11	N/A
ET 58	dp	weaner	CSFV "*Roesrath*"	0	37.24	25.68	26.71	0.0001	32.65	26.95	27.37	0.0015	30.49	26.63	27.40	0.0408	28.85	27.12	27.16	0.2066	30.09	27.09	28.29	0.0581	N/A	27.29	27.33	N/A	35.29	25.97	28.15	0.0003
				1	36.09	27.01	29.67	0.0013	43.08	27.22	28.33	0.0000	31.79	29.44	29.17	0.1067	32.23	28.33	30.68	0.1361	32.61	27.31	29.46	0.0038	N/A	27.59	27.94	N/A	N/A	28.45	29.49	N/A
				2	N/A	30.42	32.32	N/A	N/A	30.51	31.05	N/A	32.62	33.06	31.88	0.5314	N/A	30.69	34.20	N/A	34.36	31.15	32.05	0.0089	N/A	30.60	30.18	N/A	N/A	29.38	29.18	N/A
				3	29.06	16.76	19.47	0.0002	28.43	30.22	29.80	0.2173	35.81	29.54	29.40	0.0050	36.53	30.47	29.26	0.0064	32.60	30.29	30.64	0.0620	N/A	30.32	29.88	N/A	N/A	29.71	30.62	N/A
				7	29.03	21.66	23.00	0.0058	24.41	22.03	22.44	0.1403	24.01	23.76	23.28	0.4826	22.10	21.62	23.60	1.4089	30.33	22.93	23.43	0.0009	N/A	22.06	23.30	N/A	29.32	23.19	21.98	0.0026
				10	27.39	20.20	21.27	0.0061	23.67	20.17	20.82	0.0725	21.92	21.88	21.70	0.6372	19.96	19.93	21.96	1.9613	29.23	21.09	21.92	0.0007	19.67	20.14	21.84	0.6781	29.65	21.37	20.08	0.0006
				14	N/A	30.55	31.05	N/A	29.68	30.60	30.49	0.9806	35.72	32.46	32.83	0.0698	N/A	32.78	38.63	N/A	N/A	31.58	31.95	N/A	N/A	31.15	31.54	N/A	N/A	32.58	29.94	N/A
				17	31.77	24.43	25.45	0.0027	25.76	24.07	24.35	0.2534	26.79	26.30	25.13	0.3075	33.18	24.87	25.38	0.0034	32.02	24.74	25.73	0.0007	N/A	24.55	24.07	N/A	N/A	25.33	25.64	N/A
ET 59	dp	weaner	CSFV "*Roesrath*"	0	34.10	27.74	27.60	0.0034	31.58	27.97	27.64	0.0050	29.98	27.71	28.08	0.1065	29.32	28.82	28.04	0.3570	30.14	28.82	29.14	0.1325	N/A	29.13	28.18	N/A	N/A	28.01	29.16	N/A
				1	35.06	26.10	28.10	0.0011	33.03	26.02	26.95	0.0070	30.25	27.85	27.55	0.1047	26.91	26.40	28.43	1.2865	31.79	25.79	27.96	0.0025	30.20	25.99	26.24	0.0140	N/A	27.05	28.23	N/A
				2	33.57	27.97	28.12	0.0061	31.25	28.02	27.07	0.0524	28.60	29.41	27.64	0.5928	37.71	29.26	28.63	0.0021	30.98	27.94	28.49	0.0117	N/A	27.89	26.75	N/A	N/A	28.45	29.25	N/A
				3	N/A	29.09	30.25	N/A	30.80	30.24	30.07	0.3446	37.19	32.12	30.95	0.0119	N/A	34.30	34.74	N/A	36.06	31.25	31.43	0.0034	N/A	30.10	30.88	N/A	27.81	32.27	29.32	0.0004
				7	35.14	23.91	25.72	0.0004	27.93	24.28	25.24	0.0669	28.44	26.49	26.28	0.1565	27.04	25.24	27.19	0.5570	32.42	25.05	26.37	0.0011	N/A	24.85	26.13	N/A	N/A	25.97	24.70	N/A
				10	30.97	22.84	24.01	0.0031	25.58	22.88	23.30	0.1108	24.27	24.33	24.13	0.6485	23.15	22.88	24.50	1.4430	31.36	23.78	24.33	0.0008	N/A	23.41	24.20	N/A	35.76	24.27	22.67	0.0000
				14	35.05	26.12	26.70	0.0014	27.92	25.95	26.28	0.1684	29.96	27.65	27.04	0.1043	N/A	26.66	28.39	N/A	32.51	27.18	28.17	0.0040	N/A	26.24	26.96	N/A	24.99	27.42	25.50	0.8744
				21	29.52	22.53	23.32	0.0035	25.25	22.23	22.34	0.0942	23.66	24.52	23.12	0.7570	23.59	22.60	23.13	0.5568	28.07	22.31	23.42	0.0028	N/A	22.62	21.79	N/A	N/A	23.56	23.63	N/A
				28	33.26	22.88	24.03	0.0004	26.22	22.28	23.02	0.0611	24.50	24.43	23.58	0.4752	23.81	22.63	24.00	0.6529	30.22	23.04	23.99	0.0008	N/A	21.53	22.21	N/A	N/A	23.34	24.14	N/A
ET 60	dp	weaner	CSFV "*Roesrath*"	0	33.93	25.42	26.18	0.0011	28.37	25.95	26.22	0.0179	28.90	26.27	26.89	0.0940	27.40	27.22	26.66	0.4924	28.91	27.15	27.64	0.1096	N/A	27.27	26.80	N/A	N/A	26.00	27.79	N/A
				2	30.45	24.00	24.86	0.0050	27.75	24.06	24.01	0.0542	25.14	25.13	24.39	0.5124	27.60	24.22	24.85	0.1090	28.21	23.61	25.14	0.0071	N/A	23.75	23.51	N/A	N/A	26.87	27.29	N/A
				3	31.07	21.85	23.01	0.0009	26.17	27.31	27.63	0.2247	33.06	27.55	28.01	0.0112	33.05	28.12	27.66	0.0188	32.52	28.57	28.99	0.0209	43.75	28.33	28.02	0.0000	N/A	27.25	28.82	N/A
				7	30.98	23.85	24.24	0.0047	23.57	23.11	23.84	0.5813	25.71	25.09	24.58	0.3617	24.60	23.43	25.13	0.7919	29.31	24.34	24.99	0.0056	N/A	23.80	24.55	N/A	34.68	24.93	23.22	0.0001
ET 43	dp	weaner	neg.ctr."*Roesrath*"	0	36.49	26.24	27.72	0.0004	31.59	27.00	27.82	0.0040	30.77	27.02	28.12	0.0491	28.99	27.74	27.97	0.3043	30.75	28.14	29.11	0.0681	N/A	28.14	28.11	N/A	N/A	26.95	29.31	N/A
				1	40.47	28.18	30.13	0.0001	40.41	27.53	29.14	0.0001	35.66	29.73	29.39	0.0081	35.42	29.51	30.93	0.0243	33.90	27.62	29.82	0.0018	N/A	27.49	28.07	N/A	N/A	28.22	30.24	N/A
				2	N/A	30.18	29.50	N/A	34.71	28.25	28.76	0.0087	31.06	30.02	29.18	0.2207	29.44	28.92	30.60	1.1155	33.03	27.85	29.50	0.0033	N/A	28.24	27.97	N/A	N/A	29.05	30.13	N/A

				3	37.30	26.19	27.13	0.0003	26.03	26.36	26.93	0.8922	29.41	28.15	27.51	0.2114	40.56	26.60	29.08	0.0001	32.01	27.26	28.05	0.0057	N/A	26.77	27.45	N/A	19.35	27.53	26.08	1.5602
				7	29.04	20.02	20.10	0.0012	19.28	20.15	19.58	1.0007	22.65	21.83	20.37	0.2416	21.45	20.44	21.20	0.6371	26.67	21.00	20.56	0.0028	33.53	20.32	20.32	0.0000	34.07	21.78	19.13	0.0000
				10	34.28	24.56	25.66	0.0010	26.20	24.65	25.25	0.2520	27.78	26.30	26.14	0.2197	26.03	24.09	26.71	0.6383	31.68	25.51	26.33	0.0022	32.43	24.98	25.96	0.0011	N/A	26.05	24.64	N/A
				14	30.63	22.71	24.33	0.0042	22.15	23.62	24.17	2.0596	24.74	24.85	24.58	0.6490	25.83	23.33	25.34	0.3517	27.43	23.92	24.96	0.0201	N/A	23.43	24.61	N/A	N/A	24.69	23.29	N/A
				21	30.43	21.74	23.03	0.0013	24.19	21.58	22.06	0.1440	22.98	23.53	22.48	0.6992	22.25	21.43	22.55	0.7697	28.89	21.30	22.45	0.0008	32.53	21.78	21.19	0.0001	N/A	22.41	23.16	N/A
				28	39.81	26.40	26.95	0.0000	26.73	25.66	25.84	0.3739	27.99	27.62	26.28	0.3070	N/A	26.61	27.18	N/A	31.45	25.64	27.08	0.0023	N/A	26.20	25.31	N/A	N/A	26.65	27.12	N/A
ET 44	dp	weaner	neg.ctr."*Roesrath*"	0	N/A	34.80	35.66	N/A	42.20	30.35	30.55	0.0000	32.80	29.55	30.07	0.0532	29.96	30.47	29.89	0.7538	31.80	30.78	30.98	0.1458	N/A	31.22	31.09	N/A	N/A	28.91	30.95	N/A
				1	N/A	31.73	32.80	N/A	N/A	30.65	31.21	N/A	38.24	33.64	31.53	0.0106	N/A	35.27	36.31	N/A	40.94	30.06	32.34	0.0000	N/A	31.10	30.77	N/A	N/A	32.04	33.92	N/A
				2	N/A	28.02	30.07	N/A	28.61	26.12	27.29	0.1916	29.57	27.74	27.49	0.1606	22.80	26.07	27.97	0.8700	33.04	26.05	27.68	0.0010	N/A	26.38	26.49	N/A	N/A	27.05	28.08	N/A
				3	N/A	27.94	28.32	N/A	28.26	29.22	30.01	0.1947	N/A	29.00	29.72	N/A	36.40	30.64	30.15	0.0100	34.93	30.45	31.17	0.0146	41.90	30.08	29.96	0.0002	43.56	29.55	31.39	0.00
				7	28.40	20.20	20.46	0.0023	19.42	20.09	20.19	1.0901	23.00	22.13	21.08	0.2645	21.85	20.91	21.91	0.7294	26.76	21.14	21.53	0.0039	25.12	20.40	20.90	0.0092	33.24	21.50	19.66	0.0000
				10	31.68	22.22	23.63	0.0013	23.22	22.65	23.48	0.5611	24.79	24.34	24.16	0.4608	23.45	22.36	24.32	0.9165	29.58	23.38	24.15	0.0025	35.10	23.03	23.82	0.0000	N/A	24.05	22.56	N/A
				14	31.09	22.19	23.87	0.0022	21.21	22.09	23.06	1.6280	24.57	24.12	24.12	0.4916	23.78	22.90	24.54	0.9493	28.49	23.24	24.19	0.0054	N/A	22.87	24.05	N/A	27.59	24.24	22.73	0.0172
				21	31.25	22.70	23.86	0.0013	23.44	22.04	22.96	0.3973	24.23	24.31	23.45	0.5307	23.84	22.48	23.53	0.5142	28.19	22.23	23.72	0.0028	N/A	22.30	22.23	N/A	N/A	23.31	24.08	N/A
				28	39.45	28.18	29.12	0.0001	26.17	27.32	28.08	2.1867	30.55	29.47	28.32	0.1934	N/A	30.67	29.32	N/A	33.64	27.45	29.17	0.0016	N/A	27.95	27.39	N/A	N/A	28.29	29.01	N/A
ET 45	dp	weaner	neg.ctr."*Roesrath*"	0	N/A	28.05	28.50	N/A	N/A	28.99	29.38	N/A	33.51	29.10	29.69	0.0243	30.26	29.79	29.38	0.4101	32.45	30.12	30.43	0.0606	39.17	30.87	30.49	0.0018	N/A	29.27	30.94	N/A
				1	39.36	30.36	31.44	0.0007	N/A	29.98	30.67	N/A	37.02	32.83	30.76	0.0146	N/A	33.25	34.22	N/A	36.63	29.83	31.32	0.0008	N/A	29.97	30.01	N/A	N/A	30.55	31.98	N/A
				2	35.04	26.13	28.04	0.0011	30.63	26.62	26.83	0.0459	28.77	28.23	27.44	0.3288	27.87	26.73	28.15	0.6702	31.08	26.37	28.12	0.0056	N/A	26.65	26.33	N/A	N/A	27.02	27.81	N/A
				3	N/A	27.48	28.58	N/A	27.13	27.72	28.31	1.0410	34.18	29.93	29.31	0.0262	N/A	29.55	33.68	N/A	33.21	28.48	29.83	0.0065	N/A	28.36	29.29	N/A	27.69	29.24	27.52	0.4531
				7	27.47	19.45	20.64	0.0036	18.30	19.88	20.19	2.2156	22.36	21.54	21.10	0.3426	20.34	19.94	21.63	1.3490	26.49	20.98	21.27	0.0041	32.72	20.37	21.05	0.0000	33.52	21.42	19.64	0.0000
				10	29.37	20.96	21.90	0.0025	23.98	20.90	21.59	0.0971	22.20	22.39	22.09	0.7114	21.22	20.82	22.40	1.2933	29.99	21.53	22.16	0.0005	N/A	21.11	22.13	N/A	35.29	22.25	20.77	0.0000
				14	N/A	30.18	31.82	N/A	28.49	30.66	31.27	2.9580	37.30	32.79	32.07	0.0201	N/A	34.07	35.90	N/A	N/A	31.94	32.62	N/A	35.26	31.38	31.94	0.0085	N/A	31.88	29.76	N/A
				21	30.16	22.39	23.71	0.0024	20.73	20.03	20.65	0.6039	23.08	22.01	21.08	0.2368	22.21	21.13	21.70	0.5340	25.80	20.32	21.75	0.0046	34.22	20.64	20.31	0.0000	34.71	21.37	21.94	0.0000
				28	41.48	28.30	28.84	0.0000	28.68	28.00	27.77	0.4172	30.93	30.07	28.39	0.1856	N/A	29.82	30.00	N/A	37.69	28.27	29.22	0.0001	N/A	28.48	27.34	N/A	N/A	28.55	28.98	N/A
ET 46	dp	weaner	neg.ctr."*Roesrath*"	0	N/A	30.22	30.22	N/A	N/A	31.44	30.92	N/A	35.84	30.48	31.01	0.0117	30.83	31.58	30.95	0.8599	34.02	31.57	31.71	0.0492	40.76	31.29	31.10	0.0008	N/A	30.82	32.45	N/A
				1	N/A	30.50	31.77	N/A	N/A	30.32	30.84	N/A	42.08	32.69	31.46	0.0005	N/A	33.64	34.64	N/A	40.68	30.15	31.68	0.0000	N/A	29.85	29.86	N/A	N/A	30.82	31.53	N/A
				2	N/A	30.33	31.11	N/A	N/A	30.13	30.14	N/A	32.45	32.37	30.36	0.2770	N/A	33.57	33.68	N/A	35.42	30.54	31.52	0.0027	N/A	30.58	29.69	N/A	N/A	31.27	31.62	N/A
				3	29.48	21.60	21.58	0.0015	22.92	22.32	22.12	0.0807	24.98	22.44	22.29	0.0850	26.66	22.97	22.13	0.0419	26.08	22.77	23.24	0.0429	N/A	23.10	22.24	N/A	21.48	22.12	22.93	0.46
				7	31.28	22.79	23.98	0.0024	22.62	23.01	23.44	0.9445	25.56	24.89	24.56	0.3721	23.42	23.11	25.00	1.5393	28.51	24.04	24.56	0.0080	N/A	23.48	24.38	N/A	19.01	24.53	23.03	0.9677
ET 47	dp	weaner	neg.ctr."*Roesrath*"	0	42.05	28.76	28.71	0.0000	N/A	29.94	29.37	N/A	31.42	29.47	29.41	0.1098	29.75	29.98	29.46	0.6376	31.30	30.56	30.70	0.1757	N/A	30.37	29.38	N/A	N/A	29.13	30.70	N/A
				1	N/A	29.19	30.59	N/A	N/A	29.04	29.27	N/A	32.94	31.32	30.10	0.1246	42.69	31.22	32.25	0.0005	34.42	28.88	30.26	0.0021	N/A	29.09	28.80	N/A	N/A	29.76	30.52	N/A
				2	N/A	32.60	32.62	N/A	N/A	32.13	31.48	N/A	34.62	42.53	32.27	3.7581	N/A	36.08	35.46	N/A	37.04	32.19	33.41	0.0026	37.06	31.68	31.15	0.0035	N/A	37.29	32.63	N/A
				3	27.23	19.35	20.86	0.0045	22.03	19.77	20.69	0.1897	22.07	21.54	21.36	0.4568	21.67	20.28	22.19	0.7292	25.71	21.10	22.11	0.0103	34.64	20.28	21.25	0.0000	31.96	21.40	20.01	0.0001
				7	28.10	20.27	21.51	0.0041	22.72	20.69	21.10	0.1841	22.14	22.22	21.82	0.6377	21.08	20.42	22.25	1.1804	27.51	21.34	21.80	0.0026	31.17	21.02	21.99	0.0002	19.06	22.03	20.53	1.9176
				10	28.27	20.94	22.06	0.0056	24.05	21.30	21.71	0.1103	22.32	22.61	22.21	0.7337	21.22	21.03	22.64	1.5167	29.56	21.76	22.39	0.0008	N/A	21.28	22.39	N/A	N/A	22.40	20.99	N/A
				14	36.05	27.28	28.11	0.0016	27.30	27.62	27.52	0.6900	30.94	29.53	28.30	0.1512	N/A	29.44	30.19	N/A	31.24	28.69	29.06	0.0237	N/A	28.03	28.14	N/A	17.51	28.89	26.70	2.0632
				21	30.35	22.00	23.66	0.0018	27.10	22.09	22.30	0.0237	24.56	24.22	23.35	0.3909	22.63	22.13	23.17	0.9267	30.54	22.92	24.03	0.0006	N/A	22.27	22.03	N/A	N/A	23.19	23.80	N/A
				28	34.75	25.66	26.99	0.0008	27.10	25.02	25.80	0.2266	28.50	27.33	26.45	0.2054	N/A	26.40	27.50	N/A	32.70	25.53	27.24	0.0009	32.54	26.00	25.44	0.0020	N/A	26.43	27.24	N/A
ET 48	dp	weaner	neg.ctr."*Roesrath*"	0	34.37	27.89	27.98	0.0033	33.14	28.16	28.12	0.0020	29.91	27.40	28.19	0.1046	28.51	28.49	28.17	0.5876	30.39	28.58	29.38	0.1113	43.49	28.61	28.09	0.0000	N/A	27.59	29.18	N/A
				1	N/A	28.88	30.22	N/A	N/A	28.11	29.04	N/A	34.23	30.26	29.84	0.0315	38.42	30.08	31.85	0.0051	34.53	28.43	30.04	0.0016	N/A	28.90	28.49	N/A	N/A	29.21	30.53	N/A
				2	35.89	29.15	30.85	0.0044	43.60	28.89	29.51	0.0000	33.36	31.17	30.33	0.0951	39.71	30.73	33.14	0.0041	33.60	29.41	30.84	0.0058	N/A	28.94	28.98	N/A	N/A	29.50	30.86	N/A
				3	30.79	22.82	23.91	0.0034	26.04	22.86	23.41	0.0830	25.18	24.65	24.27	0.4057	23.17	22.46	24.65	1.2913	30.41	23.57	24.26	0.0015	N/A	23.33	24.25	N/A	34.65	24.56	22.96	0.0001
				7	N/A	26.29	27.08	N/A	26.33	27.39	28.17	0.2469	36.84	27.07	28.06	0.0007	29.89	28.06	28.13	0.1922	31.72	28.21	29.28	0.0368	N/A	28.20	28.46	N/A	N/A	26.87	28.86	N/A
				14	38.46	26.65	27.38	0.0002	25.77	27.02	27.21	1.4653	30.58	28.63	27.81	0.1220	36.61	27.62	29.54	0.0037	31.64	27.85	28.38	0.0104	N/A	27.11	27.74	N/A	21.01	28.17	26.14	1.7600
				21	30.51	21.53	23.43	0.0013	25.10	21.79	22.28	0.0877	23.93	24.09	23.18	0.5526	22.35	22.17	23.03	1.0938	29.61	22.07	23.13	0.0007	N/A	21.90	21.68	N/A	N/A	23.38	23.80	N/A
				28	36.12	26.83	28.00	0.0007	26.88	26.07	26.65	0.5131	29.20	28.12	27.22	0.2149	N/A	26.51	28.30	N/A	32.45	26.13	27.80	0.0016	N/A	26.56	26.34	N/A	N/A	27.13	28.07	N/A
ET 09	wb	weaner	neg.ctr."*Armenia*"	0	28.62	17.87	19.84	0.0004	23.38	18.38	20.22	0.0090	24.64	18.44	20.76	0.0167	19.13	18.17	19.70	0.6650	28.92	20.27	22.03	0.0016	29.23	19.11	20.44	0.0010	14.60	17.16	21.26	1.5973
ET 12	wb	weaner	neg.ctr."*Armenia*"	0	30.41	17.65	19.97	0.0001	23.48	18.08	20.10	0.0073	25.29	18.38	20.61	0.0098	19.80	18.37	19.70	0.4480	27.19	19.01	20.89	0.0025	28.34	19.05	20.23	0.0017	32.83	18.11	21.12	0.0000
ET 15	wb	weaner	neg.ctr."*Armenia*"	0	29.55	17.09	19.49	0.0002	23.40	17.52	19.74	0.0058	24.18	17.47	20.08	0.0133	19.13	17.56	19.45	0.4963	27.67	18.22	20.62	0.0012	28.25	18.16	19.92	0.0012	22.12	17.22	21.09	0.0285
ET 22	dp	weaner	ASV"*Armenia*"	28	27.71	17.81	19.53	0.0007	23.55	17.61	20.09	0.0060	23.97	18.73	20.43	0.0264	18.46	18.25	19.25	0.9378	27.99	19.32	20.52	0.0014	30.44	19.10	20.15	0.0004	33.09	18.38	21.09	0.0000
				34	34.07	19.03	20.78	0.0000	24.48	20.06	21.41	0.0101	26.22	20.03	21.59	0.0124	21.86	20.14	21.01	0.3042	30.57	20.95	22.36	0.0007	38.10	20.50	21.29	0.0000	34.52	19.55	22.12	0.0000
ET 23	dp	weaner	ASV"*Armenia*"	28	29.00	18.19	20.05	0.0004	23.55	18.55	20.18	0.0082	23.45	18.60	20.45	0.0368	20.71	20.09	21.15	0.6982	30.26	20.62	22.06	0.0007	30.60	19.15	20.21	0.0004	35.17	18.14	20.97	0.0000
				31	30.95	19.20	20.93	0.0002	23.68	20.17	21.86	0.0220	25.14	20.09	21.68	0.0281	20.38	19.94	20.90	0.7666	29.70	20.82	22.21	0.0012	33.90	20.81	21.72	0.0001	23.52	19.74	22.44	0.0369
				34	33.10	19.90	21.27	0.0001	24.33	20.44	21.54	0.0132	26.74	20.38	21.85	0.0105	21.63	20.97	21.29	0.5209	30.27	21.17	22.43	0.0009	36.74	21.12	21.55	0.0000	36.04	19.94	22.22	0.0000
ET 24	dp	weaner	ASV"*Armenia*"	28	27.91	18.84	19.96	0.0011	24.08	19.33	20.62	0.0082	23.15	19.57	20.82	0.0719	18.84	19.30	19.80	1.2318	28.60	20.09	20.84	0.0013	30.62	20.09	20.26	0.0005	33.01	18.89	21.11	0.0000
				31	31.66	18.26	19.81	0.0001	24.01	19.05	20.46	0.0075	23.34	18.15	19.91	0.0283	19.15	18.75	19.60	0.7719	29.37	19.69	21.19	0.0007	32.68	19.40	20.33	0.0001	30.58	18.55	20.97	0.0001
				34	33.99	18.39	20.07	0.0000	24.76	19.38	20.63	0.0051	24.08	19.00	20.62	0.0285	20.30	19.42	20.24	0.5426	30.33	20.07	21.51	0.0005	39.80	19.94	20.77	0.0000	37.54	19.38	21.68	0.0000
ET 25	dp	weaner	ASV"*Armenia*"	28	32.26	19.73	21.73	0.0001	29.69	20.17	22.36	0.0003	26.12	20.56	22.46	0.0215	21.21	20.51	21.57	0.6547	31.33	21.16	22.80	0.0005	31.63	21.01	22.05	0.0006	N/A	20.06	22.72	N/A

				31	27.62	17.97	19.64	0.0009	23.45	18.64	20.13	0.0090	23.59	18.49	20.27	0.0303	18.55	18.33	19.30	0.9143	28.92	19.63	20.88	0.0009	30.52	19.10	20.03	0.0004	33.62	18.49	21.08	0.0000
				34	29.82	17.52	19.64	0.0002	23.47	18.38	20.29	0.0086	23.55	17.87	20.15	0.0242	19.38	18.46	19.66	0.6093	29.86	19.51	21.13	0.0005	34.55	19.05	20.36	0.0000	34.60	18.66	21.46	0.0000
				36	32.01	17.95	20.07	0.0000	22.29	18.29	20.22	0.0197	24.07	18.14	20.12	0.0182	19.69	18.65	20.21	0.6318	30.35	19.75	21.69	0.0004	37.74	19.13	20.50	0.0000	36.44	18.41	21.68	0.0000
ET 26	dp	weaner	ASV"*Armenia*"	28	26.51	17.40	18.16	0.0010	23.18	18.32	18.69	0.0060	21.73	17.96	18.80	0.0568	17.50	17.96	18.25	1.1709	27.97	19.05	19.57	0.0009	34.47	19.10	19.14	0.0000	34.60	18.59	20.18	0.0000
ET 27	dp	weaner	ASV"*Armenia*"	28	32.07	19.36	21.05	0.0001	25.56	19.75	21.37	0.0040	24.47	19.98	21.36	0.0390	21.23	20.59	21.76	0.7092	31.52	21.51	23.10	0.0005	31.01	20.53	21.39	0.0007	34.86	19.24	21.95	0.0000
				31	28.32	18.38	20.00	0.0007	23.35	19.21	20.27	0.0121	24.57	19.25	20.76	0.0230	19.37	19.25	19.86	0.8576	28.98	20.14	21.09	0.0011	32.19	19.96	20.44	0.0002	35.92	19.28	21.49	0.0000
				34	30.16	18.62	20.04	0.0002	24.52	19.46	20.51	0.0059	25.48	19.46	20.64	0.0125	20.75	19.67	20.28	0.4369	29.46	20.01	21.36	0.0008	35.11	19.82	20.44	0.0000	33.45	18.89	21.14	0.0000
				36	31.05	18.61	20.09	0.0001	23.84	19.21	20.72	0.0098	23.79	18.56	20.40	0.0280	20.07	19.03	20.27	0.5611	29.67	20.10	21.42	0.0007	37.42	19.63	20.59	0.0000	17.44	18.73	21.62	1.9902
ET 28	dp	weaner	ASV"*Armenia*"	0	30.41	18.89	20.71	0.0002	25.46	19.57	21.45	0.0043	25.91	19.70	21.55	0.0136	20.20	19.66	20.69	0.7364	30.55	20.50	22.23	0.0006	29.97	20.46	21.44	0.0014	33.84	19.79	22.61	0.0000
ET 29	dp	weaner	ASV"*Armenia*"	0	38.55	18.92	21.14	0.0000	25.26	18.69	21.14	0.0034	26.70	18.77	21.42	0.0054	21.41	19.24	20.93	0.2986	30.47	19.86	22.22	0.0005	29.61	19.36	21.26	0.0011	32.18	18.61	22.18	0.0000
ET 30	dp	weaner	ASV"*Armenia*"	0	32.26	18.74	21.05	0.0001	24.31	19.28	21.15	0.0082	25.09	19.00	21.13	0.0167	20.15	18.84	20.33	0.5113	30.41	20.09	21.87	0.0005	28.28	19.51	20.77	0.0026	32.15	18.66	21.80	0.0000
ET 13	dp	adult	CSFV "*Koslov*"	0	33.50	23.58	25.41	0.0030	31.26	23.77	25.70	0.0042	29.44	25.15	26.39	0.0486	27.59	24.98	26.01	0.0893	32.74	25.51	26.79	0.0017	34.62	25.77	27.11	0.0131	N/A	25.98	25.98	N/A
				2	35.14	24.19	23.34	0.0007	28.63	22.84	23.31	0.0082	27.50	24.28	24.24	0.0651	28.20	23.95	23.76	0.0198	29.41	23.83	24.21	0.0047	N/A	25.25	25.40	N/A	N/A	25.22	24.04	N/A
				4	26.41	23.24	21.31	0.0590	25.92	23.09	21.51	0.0270	22.40	24.17	22.43	0.7702	20.42	23.31	21.28	1.5452	30.69	23.94	22.06	0.0010	N/A	24.32	22.75	N/A	N/A	25.09	21.59	N/A
				7	25.20	24.03	19.91	0.1017	23.93	23.56	19.97	0.0653	20.87	24.46	20.80	1.2821	19.07	24.06	20.03	3.2779	29.15	25.20	20.45	0.0025	N/A	25.35	21.00	N/A	N/A	25.44	19.94	N/A
				8	24.54	23.31	19.66	0.1143	23.49	22.47	19.36	0.0541	20.33	23.87	20.36	1.3289	18.31	23.55	19.32	3.6944	28.50	23.28	19.91	0.0018	N/A	24.74	20.85	N/A	N/A	25.17	19.89	N/A
ET 14	dp	adult	CSFV "*Koslov*"	0	37.51	23.87	24.91	0.0002	35.10	24.06	25.02	0.0004	29.05	25.54	25.71	0.0557	27.00	24.64	25.41	0.0985	33.12	25.66	26.11	0.0011	34.69	25.64	26.59	0.0101	N/A	26.45	25.54	N/A
				2	32.22	24.01	23.44	0.0041	29.43	23.94	24.40	0.0092	27.81	24.92	24.53	0.0698	28.13	24.11	24.00	0.0236	29.85	24.90	24.82	0.0058	33.84	25.25	25.69	0.0112	N/A	25.64	24.22	N/A
				4	26.81	24.44	21.95	0.0796	26.10	23.41	21.84	0.0289	23.08	25.01	22.62	0.6829	20.70	23.73	21.63	1.6271	31.02	24.18	22.31	0.0009	N/A	24.78	23.15	N/A	N/A	25.74	22.35	N/A
				7	24.57	24.08	19.79	0.1462	24.29	24.11	20.02	0.0610	20.95	25.40	20.76	1.5515	18.87	25.13	19.92	5.0820	29.42	25.96	20.54	0.0027	N/A	25.22	20.77	N/A	N/A	26.21	20.09	N/A
ET 15	dp	adult	CSFV "*Koslov*"	0	N/A	24.80	24.00	N/A	30.15	24.27	24.13	0.0060	27.49	25.32	24.94	0.1063	25.74	25.15	24.50	0.2044	31.32	25.97	25.11	0.0032	34.21	26.09	25.92	0.0123	N/A	26.46	24.72	N/A
				2	39.25	25.76	25.01	0.0002	30.57	25.91	26.24	0.0131	29.25	27.23	26.10	0.0870	29.62	26.77	25.81	0.0361	31.94	26.93	26.38	0.0042	34.50	27.50	26.94	0.0221	N/A	27.53	25.53	N/A
				7	26.41	23.82	20.77	0.0594	24.22	23.54	20.81	0.0698	21.91	24.28	21.60	0.8358	20.10	24.42	20.87	2.3765	30.09	24.91	21.23	0.0015	N/A	25.07	22.04	N/A	N/A	25.53	20.84	N/A
ET 16	dp	adult	CSFV "*Koslov*"	0	34.43	27.75	26.25	0.0074	33.85	26.55	26.71	0.0024	31.95	28.72	27.63	0.0409	28.72	28.04	27.30	0.1671	33.90	28.45	27.84	0.0028	N/A	28.86	28.66	N/A	N/A	29.23	27.77	N/A
				2	33.73	24.08	23.71	0.0018	30.19	24.14	24.36	0.0060	28.69	25.14	24.99	0.0502	26.64	24.56	24.43	0.0891	29.75	25.21	25.06	0.0076	34.41	25.66	25.72	0.0090	N/A	26.09	24.52	N/A
				4	30.28	26.35	24.56	0.0371	28.59	25.40	24.56	0.0235	25.56	27.16	25.20	0.5928	23.46	26.21	24.37	1.3323	33.14	26.69	25.12	0.0011	N/A	27.89	25.99	N/A	N/A	28.33	25.09	N/A
				7	26.71	24.35	21.20	0.0656	25.12	23.47	21.18	0.0439	22.26	24.73	22.07	0.8777	20.47	25.23	21.22	2.6887	30.56	25.25	21.62	0.0014	N/A	25.79	22.34	N/A	N/A	26.23	21.50	N/A
ET 17	dp	adult	CSFV "*Koslov*"	0	36.93	29.16	27.76	0.0039	37.90	28.20	27.70	0.0004	33.24	30.22	28.81	0.0400	32.84	29.38	28.36	0.0210	38.08	30.14	28.76	0.0003	N/A	30.55	29.70	N/A	N/A	30.81	28.28	N/A
				4	27.67	24.01	22.30	0.0462	26.48	23.24	22.05	0.0234	23.31	24.90	23.13	0.6735	21.39	23.90	22.23	1.3023	30.73	25.04	22.50	0.0015	N/A	25.50	23.55	N/A	N/A	25.42	22.40	N/A
				7	27.22	24.65	21.52	0.0578	25.46	24.15	21.31	0.0443	22.61	25.60	22.31	0.9631	20.81	25.29	21.41	2.3078	30.75	25.08	22.02	0.0013	N/A	25.98	22.65	N/A	N/A	26.17	21.73	N/A
ET 18	dp	adult	CSFV "*Koslov*"	0	36.99	25.81	23.64	0.0004	27.64	25.39	23.63	0.0318	28.37	27.03	24.78	0.0943	28.09	26.28	24.33	0.0542	29.05	26.66	24.69	0.0174	37.14	27.64	25.80	0.0031	N/A	27.86	24.41	N/A
				2	41.47	23.85	23.17	0.0000	30.86	23.94	23.83	0.0032	28.22	25.15	24.26	0.0535	27.29	24.62	23.99	0.0499	30.29	25.10	24.60	0.0043	N/A	25.17	25.31	N/A	37.27	25.91	24.06	0.0000
				4	29.95	23.04	23.89	0.0140	28.17	23.09	23.49	0.0122	24.71	24.44	24.39	0.3744	22.81	23.58	23.50	0.6792	32.08	24.37	24.10	0.0008	30.76	25.09	25.17	0.0582	N/A	25.16	23.84	N/A
				7	28.25	25.89	22.76	0.0648	26.03	25.10	22.68	0.0595	23.73	26.57	23.68	0.9607	21.94	25.98	22.71	2.0225	31.75	26.46	23.32	0.0015	N/A	27.28	24.07	N/A	N/A	28.38	23.07	N/A
				8	28.65	25.88	23.28	0.0591	26.68	25.30	23.05	0.0470	23.67	26.98	23.74	1.1285	22.01	26.38	22.88	2.3215	31.86	26.64	23.87	0.0018	26.72	27.14	24.38	0.1256	22.77	27.53	23.30	0.0004
ET 44	wb	weaner	Vacc. "*C-Strain*"	0	23.52	24.42	19.24	0.2599	24.03	23.65	19.25	0.0506	20.33	25.82	20.17	2.1083	17.88	26.32	19.19	11.4453	27.93	25.69	19.88	0.0058	32.00	26.50	20.23	0.0077	N/A	26.56	19.40	N/A
				0.5	25.12	25.06	20.41	0.1676	24.42	24.70	20.28	0.0708	21.22	26.25	21.07	1.8192	19.13	26.57	20.16	7.2950	28.92	26.17	20.69	0.0043	N/A	26.73	21.42	N/A	N/A	27.32	20.47	N/A
				2	22.47	24.35	18.15	0.3483	24.21	24.09	17.57	0.0308	19.27	26.09	18.93	2.9653	16.75	25.92	18.19	15.7690	26.71	24.68	18.71	0.0068	N/A	26.86	19.22	N/A	22.52	27.97	18.41	0.0002
				3	27.89	25.63	20.65	0.0786	26.55	24.76	23.90	0.5601	22.44	26.04	21.03	0.7750	20.77	24.22	22.10	4.8192	27.42	26.15	22.56	0.0244	37.89	26.02	21.30	0.0001	N/A	26.46	21.83	N/A
				7	31.08	25.94	22.79	0.0246	21.96	24.06	21.84	3.9322	24.58	26.25	23.23	0.3902	23.49	25.11	24.13	1.9854	31.33	26.67	24.71	0.0036	34.99	26.10	23.48	0.0016	N/A	26.30	23.36	N/A
				14	27.09	26.31	20.07	0.1299	23.27	24.36	20.99	1.3993	21.26	26.32	20.40	1.5605	19.97	25.43	21.18	9.3636	29.24	27.27	21.68	0.0069	44.19	26.09	20.33	0.0000	N/A	27.57	20.62	N/A
				21	28.77	28.28	21.23	0.1237	25.27	26.66	22.30	1.2936	22.01	28.50	21.44	2.6308	21.90	28.48	22.56	11.1812	30.52	28.61	23.02	0.0066	27.65	27.89	21.38	0.1058	23.85	29.22	22.34	0.9038
				28	33.27	28.82	25.21	0.0336	25.99	27.05	25.99	3.4152	27.35	29.67	26.13	0.4469	25.50	28.32	26.77	3.7266	32.59	29.95	27.12	0.0095	34.24	29.76	25.81	0.0139	22.16	31.13	26.28	0.9987
ET 45	wb	weaner	Vacc. "*C-Strain*"	0	27.46	22.23	21.16	0.0220	23.16	21.26	21.03	0.0796	23.15	22.61	21.97	0.2830	21.82	22.06	21.38	0.4041	26.59	22.38	22.03	0.0106	33.28	22.95	22.86	0.0031	33.50	24.25	22.02	0.0000
				0.5	23.42	23.83	19.00	0.2163	23.05	23.46	18.45	0.0695	19.50	24.28	19.53	1.8985	17.03	24.06	18.29	7.4364	26.08	24.57	18.98	0.0112	N/A	25.48	20.04	N/A	N/A	25.24	18.88	N/A
				3	34.24	27.66	24.33	0.0101	25.03	24.03	22.72	0.7673	34.18	27.94	24.74	0.3757	25.62	26.53	26.03	1.4119	32.87	28.12	26.33	0.0033	N/A	28.36	25.16	N/A	N/A	28.77	25.34	N/A
				7	29.77	24.72	21.65	0.0263	27.11	26.23	25.47	1.0840	22.36	25.35	22.04	0.5418	22.28	23.88	23.00	2.0325	30.72	25.81	23.57	0.0029	36.61	25.06	22.08	0.0003	N/A	25.13	22.17	N/A
				14	27.22	26.23	18.61	0.0749	24.90	24.17	19.61	0.2911	37.30	26.25	18.96	2.5836	18.47	27.52	19.68	12.7585	28.36	26.26	20.34	0.0059	37.48	25.73	19.00	0.0001	N/A	26.21	19.39	N/A
				21	26.95	26.72	19.77	0.1465	23.92	25.54	20.79	1.2540	23.08	27.46	20.48	2.2500	19.70	27.15	20.83	18.1704	29.72	28.77	21.60	0.0075	36.80	28.05	20.38	0.0003	35.27	27.57	20.45	0.0009
				28	31.47	28.84	23.56	0.0597	25.05	27.14	24.62	3.9051	30.07	29.32	24.30	0.8583	23.58	27.93	24.98	6.6792	32.10	30.22	25.55	0.0085	N/A	29.63	24.15	N/A	22.22	31.40	24.29	1.0023
ET 46	wb	weaner	Vacc. "*C-Strain*"	0	27.42	26.66	22.22	0.1144	24.37	25.44	22.05	0.1492	23.06	27.42	22.91	1.4271	20.94	26.76	21.83	3.8575	30.66	27.22	22.43	0.0031	N/A	28.46	23.52	N/A	N/A	28.71	22.32	N/A
				0.5	25.12	25.69	20.33	0.1974	24.85	25.14	20.20	0.0593	21.37	27.06	21.24	2.1787	19.18	27.47	20.23	9.6023	28.72	26.98	20.93	0.0070	N/A	27.51	21.52	N/A	N/A	28.74	20.40	N/A
				2	N/A	26.86	24.19	N/A	26.60	25.77	24.22	0.8337	32.45	26.84	24.28	0.0538	27.18	25.47	26.06	0.3277	34.48	27.09	26.65	0.0008	40.05	27.57	25.44	0.0002	N/A	27.63	25.62	N/A
				3	25.42	26.03	20.83	0.2117	24.39	25.59	20.79	0.1051	21.79	27.15	21.56	1.9017	19.23	27.98	20.65	12.5157	28.59	27.36	21.15	0.0093	N/A	28.21	22.05	N/A	22.78	29.38	21.07	0.0004
				7	31.22	26.85	23.16	0.0335	25.21	25.22	25.40	2.5642	28.56	27.45	23.41	0.6020	23.59	25.85	24.31	2.5503	32.03	28.15	25.08	0.0040	37.94	27.36	23.79	0.0004	N/A	28.14	23.95	N/A
				14	26.04	25.61	18.52	0.1229	23.08	24.13	19.66	0.9198	21.05	25.62	19.18	2.2854	18.30	25.46	19.65	17.8865	28.24	26.56	20.41	0.0072	35.11	24.70	18.84	0.0002	N/A	25.04	19.50	N/A
				21	28.06	26.10	20.14	0.0701	24.85	24.99	21.09	0.6515	25.48	26.81	20.76	1.7086	20.32	25.70	21.22	8.1214	29.91	27.39	21.97	0.0049	N/A	27.31	20.64	N/A	N/A	28.14	21.02	N/A
				28	N/A	33.22	30.21	N/A	29.09	32.57	31.25	7.2345	38.50	33.82	30.79	N/A	32.68	33.37	33.25	1.3454	39.63	33.67	33.22	0.0016	N/A	33.19	31.07	N/A	22.90	36.37	31.86	1.0294

ET 51	wb	weaner	Vacc. "*C-Strain*"	0	23.18	23.48	18.21	0.1776	22.83	23.87	18.33	0.0848	19.24	24.44	18.94	1.9408	17.07	25.58	18.16	11.2767	27.82	26.15	18.91	0.0052	N/A	25.34	19.26	N/A	N/A	26.85	18.19	N/A
				0.5	24.73	24.78	19.74	0.1608	23.76	24.15	19.49	0.0728	20.76	25.74	20.50	1.7684	18.33	26.50	19.52	9.9517	27.94	25.56	20.10	0.0059	N/A	26.18	20.98	N/A	N/A	28.07	19.90	N/A
				1	24.04	23.65	19.18	0.1479	23.36	24.34	18.74	0.0779	19.83	25.29	19.70	2.1508	17.32	26.19	18.67	13.7166	27.15	25.18	19.11	0.0066	25.31	26.58	20.30	0.4666	23.58	25.72	19.21	0.0000
				2	23.78	25.32	19.16	0.2823	N/A	24.54	18.79	N/A	20.23	27.31	20.09	3.2668	17.83	26.35	19.21	12.0766	27.49	26.51	19.93	0.0104	43.28	26.49	19.86	0.0000	21.33	29.04	19.31	0.0009
				3	30.75	25.99	22.08	0.0245	26.28	24.82	23.06	0.5035	23.74	26.56	22.59	0.6242	23.05	25.40	23.39	2.2968	31.42	26.75	23.90	0.0026	27.61	27.25	22.61	0.1352	N/A	26.64	22.63	N/A
				14	27.45	26.42	19.11	0.0811	26.25	24.41	20.15	0.1611	20.75	26.10	19.65	1.6046	19.12	26.90	20.36	21.2182	29.26	26.80	21.09	0.0048	36.59	25.96	19.59	0.0002	N/A	26.77	20.05	N/A
				21	25.98	25.16	18.55	0.1122	25.80	22.87	19.38	0.1010	19.87	25.13	19.01	1.7633	18.06	24.87	19.73	17.6710	27.70	25.70	20.02	0.0072	37.88	25.59	18.89	0.0001	N/A	26.48	19.36	N/A
				28	N/A	29.54	27.05	N/A	27.26	28.54	28.32	5.5553	31.50	30.24	27.56	0.0486	29.36	29.28	29.26	0.8218	34.87	31.29	29.82	0.0072	37.39	30.40	27.80	0.0047	22.64	31.50	28.47	0.8679
ET 47	wb	weaner	Vacc. "*CP7_E2alf*"	0	28.29	26.66	23.01	0.0855	24.51	25.75	22.69	0.1786	23.76	27.83	23.55	1.2690	22.02	27.18	22.63	2.7461	31.29	27.06	23.25	0.0025	N/A	28.54	24.15	N/A	N/A	29.41	23.12	N/A
				0.5	23.78	23.09	19.14	0.1457	23.77	23.69	19.13	0.0580	19.88	25.92	19.73	2.4827	17.85	25.51	18.94	8.3716	27.05	25.72	19.52	0.0097	24.25	25.83	20.28	0.7097	N/A	27.61	19.16	N/A
				2	23.61	24.55	18.75	0.2194	23.35	24.49	18.41	0.0743	19.50	26.03	19.39	2.9098	17.15	27.14	18.37	18.7624	26.69	25.91	19.00	0.0112	N/A	26.88	19.58	N/A	N/A	25.82	19.22	N/A
				3	38.44	28.11	24.90	0.0011	24.52	24.97	21.40	0.8884	26.49	27.53	25.00	0.2827	27.53	26.78	26.19	0.4260	33.19	27.86	26.50	0.0026	N/A	28.32	25.42	N/A	36.22	28.70	25.56	0.0030
				7	28.39	26.23	20.46	0.0658	26.64	26.06	26.03	1.6842	22.25	26.31	21.23	1.0250	21.06	25.12	21.68	4.6281	30.57	26.25	22.42	0.0025	N/A	25.72	21.11	N/A	35.06	26.24	21.30	0.0010
				14	27.56	25.78	20.19	0.0867	23.18	24.72	21.09	1.7217	22.02	26.74	21.10	1.3300	20.59	25.93	21.63	8.3698	30.03	26.91	22.00	0.0039	35.52	27.20	20.75	0.0007	N/A	26.67	20.80	N/A
				21	26.21	27.44	18.21	0.1762	27.70	25.13	19.08	0.0554	19.72	27.61	18.80	4.0263	18.04	34.15	19.25	17.7440	28.55	28.10	20.00	0.0081	35.89	26.89	18.52	0.0002	21.87	29.41	18.42	0.8637
				28	35.80	29.79	26.72	0.0159	26.06	27.86	27.48	7.1240	28.75	29.80	27.00	0.2358	27.22	29.36	28.10	2.5501	33.99	30.52	29.11	0.0083	N/A	29.66	27.05	N/A	22.88	31.27	27.77	0.6743
ET 50	wb	weaner	Vacc. "*CP7_E2alf*"	0	24.40	26.51	19.11	0.2702	23.12	24.67	18.89	0.1025	19.61	26.22	19.56	3.0314	17.18	27.21	18.25	18.1047	26.89	25.20	18.97	0.0077	N/A	27.01	20.12	N/A	22.83	27.97	19.03	0.0001
				0.5	24.05	25.09	19.39	0.2394	24.61	25.10	19.45	0.0545	20.46	26.84	20.34	2.7093	18.13	27.30	19.42	14.3073	27.82	27.24	20.13	0.0112	25.32	26.47	20.58	0.4954	22.77	29.04	19.67	0.0002
				2	24.48	26.12	19.60	0.2666	24.53	24.92	19.53	0.0561	20.27	26.50	20.19	2.6491	17.93	27.43	19.17	15.7433	26.77	26.64	19.85	0.0177	22.24	27.03	20.77	0.0138	22.80	27.93	19.54	0.0002
				3	30.30	26.43	21.73	0.0330	25.03	24.62	21.44	0.5854	23.77	27.59	22.52	0.8303	22.68	26.05	23.21	3.5110	30.30	27.74	23.48	0.0069	37.47	26.88	22.52	0.0003	N/A	27.64	22.65	N/A
				7	28.56	26.52	20.37	0.0633	25.69	25.41	22.93	0.8398	22.11	26.66	21.13	1.2272	21.03	26.34	21.71	7.3205	30.21	27.92	22.37	0.0053	35.56	26.62	20.92	0.0006	N/A	27.48	21.35	N/A
				14	27.14	27.08	19.45	0.1324	24.46	25.13	20.39	0.6809	21.16	26.42	20.11	1.5630	19.56	26.07	20.81	13.6570	28.84	27.70	21.26	0.0090	32.55	26.69	20.05	0.0027	N/A	27.75	20.20	N/A
				21	28.01	26.54	20.41	0.0894	25.05	25.28	21.44	0.7125	22.06	27.07	21.11	1.4367	21.08	25.64	21.91	5.9464	29.46	27.73	22.28	0.0083	36.30	27.12	21.04	0.0004	N/A	27.96	20.95	N/A
				28	29.73	28.04	22.62	0.0994	24.39	27.41	23.78	4.8006	23.99	29.42	23.16	1.5814	21.68	24.23	23.42	4.0976	31.33	29.81	24.66	0.0096	N/A	29.54	23.09	N/A	22.77	31.03	23.51	0.9634
ET 52	wb	weaner	Vacc. "*CP7_E2alf*"	0	23.02	24.11	18.30	0.2422	21.79	23.38	18.13	0.1334	19.42	25.13	19.16	2.2414	17.16	25.90	18.25	12.1276	27.82	26.07	18.93	0.0052	N/A	25.16	19.32	N/A	N/A	27.17	18.39	N/A
				0.5	25.17	24.91	19.63	0.1233	23.59	24.71	19.46	0.0924	20.66	25.87	20.38	1.8668	18.24	26.97	19.40	11.8327	28.34	25.66	19.90	0.0043	N/A	26.74	20.91	N/A	N/A	27.50	19.80	N/A
				1	23.81	24.69	19.70	0.2702	23.19	24.18	19.61	0.1080	20.59	25.83	20.62	2.0729	18.26	26.13	20.06	11.1781	27.56	25.72	20.46	0.0092	N/A	26.03	20.56	N/A	22.48	27.83	20.03	0.0002
				2	24.52	24.27	19.56	0.1486	N/A	23.57	19.80	N/A	20.38	25.62	20.28	2.0059	18.15	27.00	19.31	12.3355	27.26	26.11	20.02	0.0111	N/A	26.39	20.55	N/A	22.05	28.40	19.45	0.0004
				3	29.32	28.12	22.10	0.1106	24.67	26.28	23.11	2.2327	23.93	28.54	22.81	1.1064	22.02	27.56	23.29	9.7052	32.27	29.55	24.13	0.0037	32.81	28.54	22.44	0.0081	N/A	28.66	22.69	N/A
				14	26.40	25.80	18.76	0.1127	26.32	24.09	19.88	0.1276	20.29	25.79	19.29	1.7847	19.05	25.79	20.11	13.8748	29.15	26.61	20.67	0.0042	N/A	26.04	19.14	N/A	N/A	26.61	19.55	N/A
				21	25.93	25.24	19.08	0.1382	24.32	23.67	20.14	0.4295	20.57	25.38	19.72	1.4832	18.80	26.08	20.38	16.1332	28.75	26.19	20.96	0.0054	37.42	25.74	19.56	0.0001	N/A	26.66	19.72	N/A
				28	N/A	30.60	27.85	N/A	27.18	28.56	29.08	7.6693	33.86	30.94	28.30	0.0152	30.14	30.39	29.68	0.8093	35.11	32.07	30.77	0.0108	27.93	31.35	28.66	0.1985	22.73	32.64	29.63	0.5792
ET 41	dp	weaner	Vacc. "*CP7_E2alf*"	0	23.95	23.36	19.43	0.1548	22.83	23.16	19.47	0.0998	20.58	24.57	20.38	1.3900	18.08	24.15	19.31	5.2386	26.36	24.85	20.31	0.0157	N/A	24.44	20.26	N/A	N/A	28.60	19.37	N/A
				0.5	25.93	22.85	20.17	0.0499	22.38	22.03	19.98	0.1149	21.18	23.31	20.95	0.8215	19.62	23.23	20.05	1.7286	25.10	24.22	20.87	0.0380	36.66	23.86	21.44	0.0003	N/A	25.43	20.26	N/A
				1	24.52	23.71	18.84	0.1015	23.20	23.24	18.50	0.0609	19.78	24.33	19.43	1.5850	17.15	24.75	18.33	8.7080	26.04	24.46	19.01	0.0113	N/A	24.41	19.93	N/A	N/A	24.82	19.13	N/A
				2	23.55	23.77	18.95	0.1934	22.75	22.75	19.11	0.0846	20.06	25.22	19.81	1.9030	17.71	24.57	18.86	6.6134	25.51	24.62	19.40	0.0197	25.84	25.12	20.06	0.2048	22.41	25.05	19.03	0.0002
				3	32.27	25.46	22.37	0.0092	25.19	25.20	24.28	1.7329	24.01	26.45	22.89	0.5513	23.52	25.14	23.71	1.6921	30.84	26.14	24.42	0.0040	34.06	26.36	23.06	0.0026	N/A	25.65	23.27	N/A
				7	33.08	26.94	23.29	0.0118	25.46	24.13	23.42	0.7739	25.19	27.32	24.00	0.4630	24.44	26.21	24.72	1.8269	31.66	27.80	25.36	0.0051	36.31	27.60	23.91	0.0012	N/A	27.42	23.77	N/A
				14	27.42	25.63	20.00	0.0852	22.89	23.73	21.00	1.4729	21.47	25.27	20.52	0.9997	19.84	24.47	21.15	7.2642	28.51	25.80	21.81	0.0077	35.10	25.84	20.40	0.0005	N/A	25.86	20.38	N/A
				21	34.80	28.18	25.54	0.0123	29.81	26.08	26.62	0.2830	27.39	27.84	26.15	0.2443	26.36	27.15	27.14	1.5499	34.23	29.02	27.47	0.0025	35.50	28.22	26.40	0.0052	N/A	29.00	26.18	N/A
				28	35.19	29.40	26.14	0.0170	23.36	26.72	26.90	7.8256	28.09	29.95	26.82	0.3689	26.60	28.23	27.95	2.5370	31.98	30.64	28.66	0.0313	32.87	29.60	26.74	0.0407	N/A	30.27	27.20	N/A
ET 42	dp	weaner	Vacc. "*C-Strain*"	0	27.31	25.27	21.49	0.0654	23.15	24.39	21.61	0.2116	23.68	26.49	22.55	0.6927	20.96	25.58	21.70	2.5142	27.04	25.89	22.29	0.0260	N/A	26.15	22.71	N/A	N/A	27.30	21.85	N/A
				0.5	29.04	24.61	20.67	0.0146	24.21	23.27	20.59	0.0613	21.92	24.96	21.37	0.9273	20.19	23.90	20.66	1.7688	25.95	24.37	20.91	0.0218	N/A	25.95	22.27	N/A	N/A	27.18	20.83	N/A
				2	26.27	25.17	20.49	0.0882	25.22	24.52	20.63	0.0460	21.76	26.10	21.38	1.3894	19.91	25.93	20.55	3.9513	27.50	25.74	20.91	0.0112	N/A	26.22	21.81	N/A	22.42	25.61	20.89	0.0004
				3	32.33	26.22	23.20	0.0144	27.02	25.70	24.52	0.6965	25.00	27.06	23.91	0.4734	23.90	25.43	24.42	1.8403	32.88	27.09	25.34	0.0017	35.16	27.05	24.05	0.0022	35.85	27.49	24.53	0.0021
				7	32.94	27.82	23.49	0.0178	25.73	25.25	24.31	1.2687	25.46	27.55	24.20	0.4421	24.65	26.59	25.02	2.0054	31.47	27.61	25.38	0.0056	34.26	27.50	24.05	0.0043	N/A	28.20	24.15	N/A
				14	27.73	26.11	19.87	0.0786	24.95	25.10	20.94	0.6002	21.48	26.52	20.47	1.4605	20.05	25.47	21.07	8.6030	28.77	26.85	21.56	0.0081	36.81	27.25	20.29	0.0003	N/A	27.29	20.53	N/A
				21	29.61	26.10	21.10	0.0372	26.36	25.05	22.06	0.3627	22.17	26.64	21.43	1.2925	22.03	24.68	22.30	2.4905	30.55	27.70	22.79	0.0045	N/A	27.09	21.34	N/A	22.83	27.43	21.65	0.8749
				28	34.26	29.30	25.64	0.0245	26.16	28.15	26.47	5.1248	27.33	29.50	26.29	0.4545	25.92	28.90	27.11	3.8105	33.44	29.81	27.68	0.0060	38.25	29.98	25.96	0.0014	22.90	31.01	27.14	0.9756
ET 43	dp	weaner	Vacc. "*C-Strain*"	0	25.72	23.16	20.42	0.0668	21.94	23.06	20.61	0.2354	21.58	24.19	21.26	0.8966	19.53	24.04	20.56	2.8208	26.60	24.21	21.04	0.0137	N/A	25.14	21.65	N/A	35.59	28.85	20.63	0.0000
				0.5	25.70	24.18	19.30	0.0651	23.07	23.10	19.15	0.0769	20.20	24.30	20.00	1.4428	18.32	24.57	19.12	4.7553	25.01	24.52	19.64	0.0295	30.78	24.73	20.50	0.0106	N/A	25.17	19.51	N/A
				2	25.47	23.53	20.28	0.0830	22.52	22.41	20.06	0.1184	21.16	23.70	21.03	0.9463	19.35	23.58	20.24	2.4734	25.41	23.65	20.53	0.0225	N/A	23.99	21.38	N/A	22.86	25.01	20.41	0.0001
				3	35.93	26.01	24.03	0.0020	26.79	24.96	24.91	0.7301	26.65	26.04	24.39	0.1281	27.12	24.75	25.63	0.2291	30.78	26.34	26.50	0.0091	N/A	26.21	25.19	N/A	N/A	26.23	25.15	N/A
				7	33.83	27.18	23.71	0.0092	24.92	24.28	25.19	2.1213	25.39	26.71	24.28	0.3667	25.09	25.67	25.13	1.1136	32.05	27.69	25.72	0.0042	N/A	27.38	24.36	N/A	N/A	27.36	24.49	N/A
				14	27.35	25.33	19.82	0.0767	22.60	24.07	20.75	1.7947	21.45	25.61	20.40	1.0859	19.85	23.01	21.07	4.2242	28.48	25.72	21.51	0.0069	N/A	25.28	20.28	N/A	34.87	26.37	20.61	0.0009
				21	26.64	27.39	17.82	0.1190	24.38	25.33	18.85	0.4406	19.25	27.35	18.32	4.3719	17.90	32.60	19.05	12.7000	28.35	27.75	19.57	0.0072	27.90	27.66	18.17	0.0310	N/A	27.21	18.75	N/A
				28	28.02	27.42	19.66	0.0925	22.63	26.11	20.72	3.2919	21.40	28.24	20.51	2.7136	19.99	29.04	20.73	17.3316	28.35	28.34	21.60	0.0178	N/A	28.11	20.34	N/A	22.88	28.98	20.68	0.8901
ET 53	dp	weaner	Vacc. "*C-Strain*"	0	24.61	23.29	19.50	0.1038	22.50	23.62	19.64	0.1445	20.75	24.64	20.38	1.2801	18.80	24.91	19.47	4.2864	27.28	24.41	20.11	0.0066	38.18	25.27	20.85	0.0002	N/A	25.68	19.56	N/A

				0.5	25.64	23.39	19.15	0.0514	23.04	23.14	19.02	0.0763	20.08	24.07	19.74	1.3486	17.66	23.74	18.71	5.0040	24.92	24.29	19.33	0.0264	N/A	24.79	20.17	N/A	N/A	25.91	19.20	N/A
				1	24.10	24.75	19.38	0.2089	24.10	24.01	19.45	0.0563	20.55	25.94	20.28	1.9796	18.29	26.03	19.40	8.4830	26.33	26.46	19.99	0.0239	26.90	25.74	20.44	0.3987	35.56	25.14	19.36	0.0000
				2	26.35	25.31	20.52	0.0885	N/A	23.81	18.78	N/A	21.97	25.85	21.56	1.2090	19.53	25.39	20.39	4.0932	26.35	24.84	21.10	0.0204	N/A	25.94	21.75	N/A	22.27	28.55	20.53	0.0004
				3	38.95	26.73	25.32	0.0006	27.09	25.32	25.49	0.8332	27.89	26.45	25.53	0.0904	28.26	25.43	26.87	0.2011	31.09	27.13	27.48	0.0132	36.34	27.41	26.10	0.0023	N/A	27.11	26.19	N/A
				7	35.85	26.49	24.32	0.0027	25.87	24.41	26.01	1.6315	26.56	26.59	25.09	0.2046	26.21	25.25	25.94	0.5779	30.83	26.94	26.44	0.0104	37.62	26.37	25.04	0.0006	N/A	27.38	25.10	N/A
				14	36.94	25.44	20.49	0.0003	24.18	24.03	21.77	0.9402	22.61	25.54	21.12	0.6048	20.88	23.74	21.80	3.4049	28.75	25.91	22.46	0.0084	N/A	25.21	21.16	N/A	N/A	26.31	21.23	N/A
				21	26.71	25.80	18.95	0.0996	26.23	25.04	19.99	0.1885	20.06	26.59	19.35	2.7552	19.22	27.13	20.10	19.5731	29.55	26.71	20.70	0.0033	44.46	25.97	19.23	0.0000	35.26	27.14	19.84	0.0007
				28	32.66	29.29	24.63	0.0467	26.42	28.40	25.76	3.6501	26.56	30.40	25.65	0.8293	24.80	29.00	26.13	6.1493	33.89	30.95	26.88	0.0047	44.49	30.74	25.47	0.0000	21.72	32.48	26.32	0.7924
ET 54	dp	weaner	Vacc. "*C-Strain*"	0	24.79	25.35	18.98	0.1460	21.31	23.98	18.96	0.2426	20.04	25.51	19.73	2.0313	17.64	25.52	18.63	8.7617	26.47	24.93	19.36	0.0109	39.23	26.74	19.97	0.0001	36.38	28.38	18.84	0.0000
				0.5	25.02	23.57	19.08	0.0772	23.48	24.39	20.24	0.0708	19.99	25.49	19.83	2.1530	17.89	24.96	18.72	6.3303	25.52	24.98	19.37	0.0217	N/A	26.22	20.20	N/A	N/A	27.06	19.15	N/A
				1	25.79	25.34	20.24	0.1153	24.01	24.92	21.48	0.0832	21.55	26.36	21.16	1.5801	19.64	26.44	20.39	5.3049	26.24	26.43	20.86	0.0338	36.81	26.54	21.44	0.0006	35.63	25.69	20.37	0.0000
				2	27.16	24.75	21.76	0.0666	24.62	22.46	20.79	0.0943	22.58	26.30	22.43	1.2267	20.43	26.14	21.49	4.0225	26.73	26.42	22.13	0.0361	37.04	26.61	22.93	0.0009	22.28	28.57	22.01	0.0006
				3	39.37	27.88	25.45	0.0007	25.66	25.22	25.20	1.7912	27.31	28.03	25.91	0.2532	27.63	27.03	27.07	0.5900	31.07	28.16	27.66	0.0197	35.56	28.44	26.13	0.0049	N/A	29.11	26.16	N/A
				7	35.19	27.32	24.15	0.0049	25.08	25.97	26.31	4.8583	26.62	27.11	24.86	0.2145	26.04	25.59	25.72	0.6771	30.98	27.27	26.22	0.0096	42.38	26.72	24.73	0.0000	N/A	27.81	24.83	N/A
				14	35.13	26.42	24.45	0.0042	26.43	24.44	25.32	0.9038	25.64	25.91	24.85	0.2875	25.50	24.91	26.05	0.8895	29.48	25.90	26.54	0.0206	35.42	26.28	25.03	0.0021	N/A	25.98	24.95	N/A
				21	28.33	28.63	19.24	0.0971	26.18	26.02	20.43	0.3087	20.66	28.02	19.84	3.3602	19.48	30.48	20.41	19.4897	30.30	28.96	21.12	0.0045	37.09	27.78	19.59	0.0002	22.72	28.82	20.23	0.8205
				28	37.40	30.20	26.94	0.0074	26.82	29.11	28.11	8.1161	29.63	30.54	27.74	0.2081	27.39	28.91	28.44	2.1793	36.21	30.89	29.06	0.0018	27.10	30.58	27.52	0.4532	21.68	33.17	28.63	0.9962
ET 55	dp	weaner	Vacc. "*C-Strain*"	0	26.68	22.91	20.89	0.0398	23.08	22.00	20.13	0.1059	22.25	23.89	21.72	0.6347	20.33	23.49	20.81	1.4827	27.44	23.55	21.27	0.0066	N/A	24.26	22.20	N/A	N/A	28.67	21.23	N/A
				0.5	25.71	22.63	20.28	0.0551	23.96	23.80	20.05	0.0451	20.53	23.17	20.88	1.1401	19.23	22.95	19.97	2.0073	25.88	22.37	20.75	0.0116	37.80	24.09	21.66	0.0002	22.40	25.25	20.41	0.0001
				1	25.69	24.78	20.39	0.1089	24.30	22.77	21.38	0.0565	21.09	25.57	21.04	1.6244	19.16	25.27	20.12	4.6323	26.99	25.26	20.59	0.0125	N/A	25.27	21.28	N/A	22.71	23.81	20.42	0.0002
				2	27.24	24.33	21.48	0.0516	23.82	24.13	20.38	0.0866	22.53	24.07	22.16	0.6421	20.88	23.99	21.37	1.4328	26.81	23.55	21.83	0.0124	24.19	25.29	22.81	1.4875	22.35	27.67	21.75	0.0003
				3	37.72	25.41	24.44	0.0007	26.07	23.85	24.83	0.7932	28.89	25.30	24.41	0.0216	28.88	25.00	26.41	0.0952	31.49	25.97	27.07	0.0060	43.71	26.12	25.45	0.0000	N/A	26.04	25.69	N/A
				7	N/A	25.50	23.90	N/A	26.07	23.65	25.21	0.8542	27.23	24.87	23.90	0.0502	26.10	24.15	25.47	0.3631	30.44	25.61	26.17	0.0083	35.73	25.47	24.67	0.0013	34.17	25.60	24.79	0.0037
				14	27.71	25.81	20.18	0.0800	24.10	23.65	21.03	0.6751	21.23	25.32	20.47	1.1815	20.27	25.03	21.40	7.1388	28.63	25.61	22.07	0.0072	31.79	25.21	20.42	0.0032	N/A	24.75	20.75	N/A
				21	27.21	25.36	19.47	0.0753	25.01	24.14	20.59	0.3783	21.22	26.05	20.57	1.5505	20.16	24.70	20.94	5.8441	30.23	26.53	21.52	0.0025	35.90	26.38	20.13	0.0003	22.79	27.63	20.41	0.6516
				28	32.87	28.41	25.03	0.0356	25.78	27.65	26.11	4.9122	26.91	28.94	25.76	0.4265	25.22	27.63	26.42	3.1617	33.43	29.38	27.09	0.0043	N/A	29.09	25.62	N/A	35.80	30.95	26.34	0.0075
ET 56	dp	weaner	Vacc. "*CP7_E2alf*"	0	28.78	24.59	20.59	0.0166	22.30	22.78	20.20	0.2318	22.01	25.45	21.30	0.9852	19.41	24.87	20.33	3.6856	26.82	25.17	20.96	0.0155	34.78	26.12	21.76	0.0022	N/A	26.49	20.70	N/A
				0.5	24.77	22.85	20.03	0.0970	23.19	24.42	20.03	0.0898	21.16	23.58	21.20	0.9565	19.30	23.82	20.30	2.8138	25.77	24.30	20.78	0.0233	16.87	23.89	21.32	0.8256	22.98	26.54	20.09	0.0001
				1	25.33	24.56	20.12	0.1171	24.14	24.35	20.31	0.0726	21.20	26.00	20.90	1.6381	19.42	25.90	20.15	4.7838	26.57	26.13	20.51	0.0215	N/A	27.14	21.23	N/A	39.97	25.70	20.32	0.0000
				2	25.56	25.01	20.44	0.1281	24.17	23.78	19.98	0.0760	21.20	26.23	21.11	1.8500	19.45	25.84	20.34	4.9165	27.01	25.51	20.76	0.0141	N/A	26.10	21.59	N/A	N/A	28.11	20.58	N/A
				3	37.42	25.94	25.49	0.0013	25.74	25.01	24.40	1.2040	28.22	26.06	25.43	0.0614	29.37	25.61	27.45	0.1206	31.97	27.24	27.95	0.0085	33.79	26.52	26.24	0.0088	N/A	27.38	26.80	N/A
				7	32.78	26.91	23.52	0.0150	25.91	24.95	26.29	2.0654	25.11	26.63	23.96	0.3893	24.32	25.38	24.83	1.5504	31.49	27.35	25.31	0.0049	34.22	27.13	24.15	0.0041	34.98	26.94	23.76	0.0024
				14	28.70	26.12	19.61	0.0410	23.96	25.35	21.03	1.2518	21.07	26.28	20.15	1.6085	19.58	26.29	20.95	15.3127	28.79	26.76	21.39	0.0073	28.31	25.88	20.10	0.0277	22.45	26.10	20.47	0.5774
				21	28.05	26.66	20.21	0.0855	25.35	24.57	21.30	0.4497	21.58	27.01	20.84	1.7942	20.43	26.48	21.32	10.2411	30.25	27.32	21.98	0.0037	35.61	27.21	20.74	0.0006	22.92	28.93	21.02	0.9468
				28	N/A	27.84	21.50	N/A	27.33	29.61	30.55	8.2845	32.76	31.19	29.41	0.0512	31.35	30.09	30.86	0.4747	41.99	31.72	31.56	0.0001	40.15	30.94	29.81	0.0020	22.44	33.16	30.47	0.5687
ET 57	dp	weaner	Vacc. "*CP7_E2alf*"	0	26.03	24.03	20.16	0.0661	22.71	23.77	19.51	0.1457	21.15	25.41	20.88	1.4301	18.99	24.76	19.94	4.1669	26.03	24.55	20.42	0.0186	N/A	25.42	21.35	N/A	N/A	26.55	20.36	N/A
				0.5	24.72	24.25	19.38	0.1242	23.08	24.26	20.32	0.1009	20.37	25.40	20.34	1.9417	18.80	25.02	19.47	4.4171	25.04	25.51	20.06	0.0457	27.65	25.03	20.59	0.0792	N/A	26.02	19.58	N/A
				1	26.29	24.93	20.63	0.0849	24.03	23.59	20.45	0.0814	21.25	25.78	21.21	1.6523	19.53	25.45	20.42	4.2321	26.24	25.76	20.94	0.0281	N/A	26.18	21.80	N/A	22.99	26.85	20.77	0.0002
				2	25.81	24.29	20.57	0.0927	24.10	23.11	20.47	0.0682	21.34	25.21	21.31	1.3840	19.47	25.14	20.36	3.9047	26.91	24.35	20.78	0.0105	N/A	26.05	21.97	N/A	22.48	27.62	20.88	0.0002
				7	27.08	25.02	19.15	0.0668	24.14	23.29	20.15	0.4309	20.12	25.27	19.33	1.7198	19.33	24.93	20.05	8.3013	29.04	25.73	20.93	0.0038	34.14	24.60	19.38	0.0005	N/A	25.15	20.00	N/A
				14	29.20	25.59	20.92	0.0387	23.31	24.37	22.05	1.9921	22.67	25.95	21.93	0.8685	20.40	24.01	22.10	5.8575	28.82	26.58	22.72	0.0108	35.81	26.05	21.63	0.0005	29.73	26.82	21.97	0.0222
				28	N/A	27.86	19.89	N/A	26.13	24.45	21.16	0.2509	21.78	26.24	21.07	1.3186	21.01	25.09	21.44	4.3762	30.25	27.06	22.02	0.0035	37.79	26.53	20.67	0.0001	22.78	28.29	21.06	0.9051
				21	28.91	25.73	20.19	0.0382	25.12	26.73	24.63	3.3188	25.27	28.85	24.31	0.7954	23.36	27.63	24.82	6.6678	32.07	29.20	25.56	0.0064	24.24	29.03	24.16	0.6795	22.41	30.38	24.47	0.8956
ET 58	dp	weaner	Vacc. "*CP7_E2alf*"	0	25.90	22.86	20.61	0.0582	23.95	24.52	19.83	0.0667	21.30	24.19	21.36	1.0889	19.33	24.28	20.21	3.1092	27.45	23.91	20.99	0.0067	N/A	25.02	21.88	N/A	23.05	27.01	20.62	0.0001
				0.5	24.99	24.41	19.64	0.1190	25.05	25.92	22.91	0.0401	20.57	25.50	20.59	1.8985	18.78	26.02	19.72	6.7031	27.50	25.52	20.08	0.0080	N/A	25.85	20.84	N/A	N/A	25.24	19.90	N/A
				1	28.43	26.35	23.08	0.0731	26.43	24.05	20.17	0.0616	23.90	27.31	23.72	1.0677	22.38	27.24	23.05	2.5074	31.08	27.35	23.18	0.0031	N/A	27.35	24.27	N/A	22.84	27.85	23.16	0.0005
				2	25.15	23.36	20.38	0.0994	23.87	25.71	21.46	0.0813	20.95	24.18	21.06	1.2233	18.80	23.97	20.05	3.8638	26.92	23.67	20.41	0.0074	38.24	25.03	21.62	0.0002	22.68	28.83	20.47	0.0001
				3	35.49	25.85	24.79	0.0032	24.95	24.52	19.42	0.2938	26.47	26.00	24.99	0.1748	26.48	25.11	25.96	0.4592	32.86	25.82	26.87	0.0020	N/A	25.91	25.23	N/A	N/A	27.21	25.95	N/A
				7	26.49	26.18	18.54	0.1122	27.64	24.86	25.77	0.5655	19.40	26.26	18.67	3.1220	18.44	27.56	19.42	30.6636	29.16	27.04	20.29	0.0042	35.42	25.67	18.49	0.0002	N/A	27.29	19.39	N/A
				14	28.19	24.75	20.05	0.0416	23.43	23.14	20.95	0.8561	21.30	25.18	20.44	1.0665	20.05	23.57	21.19	4.6402	29.84	25.33	22.04	0.0028	34.23	24.74	20.35	0.0006	35.45	26.57	20.82	0.0007
				21	27.48	26.02	19.02	0.0685	27.77	24.93	20.08	0.0710	20.56	26.93	19.73	2.4504	19.80	27.55	20.28	15.9397	29.97	27.47	20.94	0.0033	N/A	26.83	19.62	N/A	22.70	28.84	20.03	0.7858
				3	35.49	25.85	24.79	0.0032	24.95	24.52	19.42	0.2938	26.47	26.00	24.99	0.1748	26.48	25.11	25.96	0.4592	32.86	25.82	26.87	0.0020	N/A	25.91	25.23	N/A	N/A	27.21	25.95	N/A
ET 59	dp	weaner	Vacc. "*CP7_E2alf*"	0	26.52	25.11	21.29	0.0949	25.11	23.92	18.44	0.0851	22.59	26.72	22.26	1.2900	21.10	27.06	21.55	3.4569	28.76	27.66	22.00	0.0121	32.42	26.37	22.47	0.0122	N/A	24.39	21.17	N/A
				0.5	23.80	24.41	18.87	0.1957	23.99	23.99	19.82	0.0438	20.03	25.32	19.77	1.9694	17.72	26.69	18.87	12.9838	26.42	24.88	19.24	0.0106	N/A	26.14	20.05	N/A	N/A	28.23	19.10	N/A
				3	33.58	25.91	23.48	0.0068	24.86	25.26	18.98	0.3353	25.73	26.07	24.27	0.2348	25.67	24.59	25.11	0.5037	31.23	26.00	25.57	0.0043	N/A	26.04	24.27	N/A	N/A	27.04	24.30	N/A
				7	26.75	26.66	18.32	0.1038	26.41	24.13	24.53	0.6284	20.23	27.33	19.18	2.9335	18.51	30.56	19.64	16.2498	29.08	27.47	20.45	0.0053	38.21	27.76	19.17	0.0001	22.78	28.00	19.44	0.5341
				14	38.91	26.92	20.30	0.0001	24.58	25.34	21.40	0.9604	22.37	26.56	21.10	0.9811	20.36	26.49	21.78	12.6841	29.26	27.34	22.24	0.0084	35.91	26.71	20.75	0.0005	N/A	28.08	21.03	N/A
				21	29.45	28.06	17.45	0.0242	26.59	26.41	18.58	0.1398	19.57	28.24	18.25	4.5280	17.85	36.63	18.99	9.3400	28.74	28.73	19.44	0.0071	35.36	27.93	17.96	0.0004	N/A	30.08	18.59	N/A

				28	33.18	28.86	25.19	0.0356	26.56	27.89	26.33	3.5001	26.67	29.19	25.66	0.5275	25.19	27.70	26.51	3.4156	33.90	29.34	27.30	0.0032	23.37	29.30	25.61	0.2167	22.69	31.56	26.71	0.5867
ET 60	dp	weaner	Vacc. "*CP7_E2alf*"	0	26.39	23.51	19.96	0.0431	21.93	23.04	19.02	0.2361	21.02	25.27	20.63	1.3822	18.95	24.03	19.97	3.4437	25.07	25.20	20.31	0.0440	32.31	25.06	20.96	0.0054	N/A	28.02	19.96	N/A
				0.5	24.73	23.01	19.21	0.0816	23.50	24.02	19.29	0.0561	20.39	23.68	19.91	1.0630	17.87	24.18	18.81	5.1676	25.19	23.18	19.35	0.0154	N/A	24.56	20.26	N/A	N/A	26.19	19.31	N/A
				1	24.76	24.33	19.94	0.1469	23.85	24.02	19.29	0.0628	20.02	24.53	20.27	1.8566	17.86	25.20	19.21	8.2244	25.33	24.16	19.88	0.0228	N/A	25.59	20.90	N/A	23.00	25.93	19.86	0.0001
				3	38.02	26.23	24.53	0.0007	22.36	22.76	20.44	1.2440	27.55	26.49	24.87	0.0928	28.28	25.82	26.39	0.1919	29.83	25.92	26.58	0.0164	38.34	26.21	25.23	0.0004	N/A	26.66	25.25	N/A
				7	28.80	25.08	19.57	0.0276	24.27	23.97	25.45	3.1971	21.46	25.09	20.21	0.8611	19.97	23.54	20.94	4.4626	28.07	25.06	21.33	0.0071	34.78	24.84	20.02	0.0004	N/A	25.74	20.37	N/A
				14	35.73	25.65	20.88	0.0008	23.28	24.30	21.82	1.8353	23.16	25.64	21.35	0.4602	21.41	23.38	22.09	2.2919	29.56	25.57	22.64	0.0045	35.36	25.40	21.29	0.0005	35.16	27.05	21.35	0.0011
				21	27.99	27.20	19.20	0.0764	27.42	25.32	20.25	0.1062	20.64	27.47	19.70	2.7292	20.12	29.09	20.47	18.3327	30.05	28.24	21.09	0.0042	36.56	26.49	19.45	0.0002	22.33	27.61	20.16	0.7693
				28	N/A	29.54	28.07	N/A	27.94	28.35	29.01	4.3277	32.97	30.09	28.26	0.0213	30.98	28.95	30.03	0.3070	37.14	30.38	30.59	0.0014	39.24	30.28	28.86	0.0021	22.63	32.42	30.21	0.9834
				2	42.31	25.67	24.19	0.0000	24.67	24.47	25.25	2.7072	N/A	29.87	29.52	N/A	28.37	25.21	26.49	0.1511	30.04	26.12	26.68	0.0155	N/A	25.94	25.02	N/A	N/A	26.20	25.25	N/A
ET 285	dp	weaner	Vacc. "*CP7_E2alf*"	0	24.21	23.39	19.00	0.1172	23.17	23.64	19.05	0.0808	20.16	24.85	19.79	1.6164	18.01	25.03	18.90	6.3616	26.23	24.83	19.50	0.0130	N/A	25.73	20.33	N/A	N/A	25.64	19.04	N/A
				0.5	25.52	23.98	20.39	0.0957	24.50	23.50	20.20	0.0486	21.23	24.90	21.09	1.2669	19.37	24.79	20.16	3.5046	27.19	24.89	20.65	0.0098	N/A	25.02	21.53	N/A	N/A	26.40	20.48	N/A
				1	23.62	23.15	19.47	0.1802	23.73	22.88	19.01	0.0464	20.69	24.72	20.58	1.4378	18.12	24.56	19.67	6.5260	27.00	25.20	20.31	0.0111	20.43	24.54	20.29	0.4982	22.47	27.31	19.59	0.0002
